# Design of ionic liquids containing glucose and choline as drug carriers, finding the link between QM and MD studies

**DOI:** 10.1038/s41598-022-25963-z

**Published:** 2022-12-19

**Authors:** Sepideh Kalhor, Alireza Fattahi

**Affiliations:** Department of Chemistry, Sharif University of Technology, Tehran, Iran

**Keywords:** Ionic liquids, Computational chemistry, Molecular dynamics

## Abstract

Designing drug delivery systems for therapeutic compounds whose receptors are located in the cytosol of cells is challenging as a bilayer cell membrane is negatively charged. The newly designed drug delivery systems should assist the mentioned drugs in passing the membrane barriers and achieving their targets. This study concentrated on developing novel ionic liquids (ILs) that interact effectively with cell membranes. These ILs are based on glucose-containing choline and are expected to be non-toxic. The binding energies of the known pharmaceutically active ionic liquids were calculated at the B3LYP/6-311++G(d,p) level in the gas phase and compared with those of our newly designed carbohydrate-based ionic liquids. Subsequently, we employed MD simulations to obtain information about the interactions of these known and designed ILs with the cell membrane. In our approach, we adopted QM and MD studies and illustrated that there could be a link between the QM and MD results.

## Introduction

Ionic liquids (ILs) are molten salts, mainly organic cations and inorganic anions^1^. The first study of these chemicals with the properties such as moisture stability at room temperature was reported in 1992^2^. In the history of ILs, the focus has been on their chemical synthesis and their applications as catalysts^3,4^. Furthermore, ionic liquids have been utilized in electrochemistry as electrolytes. In particular, ionic liquids based on asymmetric ammonium structures alongside bis (trifluoromethyl sulfonyl) imide (Tf_2_N) have purported significant electrochemical stability, making them ideal candidates for designing transistors-like systems^5^. The capability of ionic liquids as electrolytes to affect the stability of the electrochemical window has been confirmed by theoretical studies^6^.

Moreover, the applications of ILs in biotransformation^7^, biotechnology^8^ as well as extractive solvents have been vastly studied^9^. Recently, the extraction of polyphenols from Chestnut shells by combining natural deep eutectic solvents and bio-ionic liquids has been reported^9^.

Fractionation and depolymerization are two pivotal steps in the biomass valorization procedure. In these types of operations, the effectiveness of ionic liquids has been documented. For example, the pretreatment of lignocellulosic and algal biomass by ionic liquids can complete the two mentioned steps^10^.

The last decades have seen a renewed importance of ionic liquids as green solvents due to their spectacular physical and chemical properties, such as high thermal stability^11^. Ferdeghini et al. have recently designed six N-morpholinium di-cationic ionic liquids representing high thermal stability^12^. Recently, Clarke and co-workers have reported the synthesis of di-cationic ionic liquids bearing pyridine functional groups expected to be applicable as efficient ligands for metals for catalyzing specific reactions at raised temperatures and other tasks^13^. Lack of inflammability, low volatility, chemical stability, and good solubility are other characteristics of ionic liquids which make them preferable to many organic solvents^11^. Also, the low volatility quality of ionic liquids has been enhanced by incorporating phosphonium as cations in their structures^14^.

Moreover, the mentioned features of ILs have enabled them to be considered a suitable medium for both proteins and DNA^15^. For instance, Chevrot et al., by applying molecular dynamics studies, have illustrated that ionic liquids with amino acid-based anions as persevering solvents in combination with water can prevent the denaturation of proteins at elevated temperatures^16^. Following that, this research group, with the aid of molecular dynamic simulations, has found that the aqueous solutions of amino acid-based ionic liquids such as five mol% aqueous amino acid-based ILs mixtures can increase the stability of mini-protein structures up to 30–40 K^17^.

There are many studies on the anticancer effect of ILs on 60 cell lines, such as breast cancer, melanoma, cervical and ovarian cancers, and hepatocarcinoma^18–20^. The ionic liquids also have many uses in internal and external drug delivery^21,22^. Suksaeree et al. have developed lidocaine–diclofenac ionic liquid drug, which can have a developing effect on controlled drug release^23^. Notably, many choline-based ionic liquids have been designed as anti-inflammatory drugs to improve the interaction of anti-inflammatory and antipyretic drugs with biological membranes. The choline-based ILs have increased the solubility of these drugs^24^. *Choline* is a molecule playing a crucial role in various critical functions in the human body^25^. For example, it is one of the components of Cytidine 5'-diphosphocholine (CDP-choline), an invaluable intermediate in the biosynthetic pathway of the structural phospholipids of cell membranes. The role of CDP-choline in activating phospholipid biosynthesis in neuronal membranes cannot be ignored^26^. Also, the application of choline in synthesizing vaccine carriers such as n-hexadecyl choline phosphate (C16-CP) for anticancer immunotherapy has been investigated. Accordingly, using n-hexadecyl choline phosphate in the vaccine formulations can thrive antigen-specific immune responses, which consist of humoral and cellular immune responses and immune memory^27^.

The presence of hydrogen bond networks in the cation or anion of ILs can help delocalize the positive charge in the cation and the negative charge in the anion. This charge delocalization decreases the interactions between anion and cation of ILs, decreasing the melting points and viscosities^28^. Designing new ILs with choline and hydrogen-bond network in their structures can be pursued by combining choline and carbohydrates to synthesize ILs^29^. Some imidazolium-based ionic liquids, such as 1-octyl-3-methylimidazolium (M8OI), have been found as an initiator of the liver disease primary biliary cholangitis (PBC)^30^. Thus, designing ILs with low toxicity and biodegradability capacity has attracted much attention^29,30^.

Many of the choline-based ILs derivates, such as 2-hydroxyethyl-trimethylammonium-L-phenylalaninate [Cho][Phe] and the 2-hydroxyethyl-trimethylammonium-L-glutaminate [Cho][Glu], enhance solubility for both poorly soluble ferulic acid and rutin. At the same time, they do not influence the antioxidant activity of both drugs (ferulic acid and rutin)^31^. Moreover, it is illustrated that choline-based ILs lower toxicity more than imidazolium-based ILs^32^. The other approaches for the combination of drugs and ILs are as follows: (1) using active pharmaceutical materials as anions or cations in the structures of ILs, (2) covalently adding drugs to the cations of ILs (this kind of ILs have been designed and synthesized based on imidazolium)^33^. In this study, we designed the ILs in which the cationic part is the *N,N,N*-trimethyl-2-(((2*R*,3*R*,4*S*,5*S*,6*R*)-3,4,5-trihydroxy-6-(hydroxymethyl)tetrahydro-2H-pyran-2-yl)oxy)ethan-1-aminium (including glucose and choline) along with various pharmaceutically active materials as the anionic part of the designed ILs. The synthesis of the cationic part of these ILs has been reported^29^. The use of carbohydrates in the drug structures is expected to increase water solubility and the affinity of drugs toward their receptors and decrease toxicity^34–36^. Besides, many cells, such as viruses, bacteria, and eukaryotic cells, bear glycans on their surfaces, playing essential roles in recognition, communication, and invasion. These aforementioned interactions can turn the mentioned carbohydrate-based molecules into the prospective target for therapy or diagnostic procedures^37–39^. Around 54 carbohydrate-based compounds have been identified for therapeutic or diagnostic purposes from 2000 to 2021^40^.

Imidazolium-based ionic liquids have been modified by the covalent addition of Glucono-δ-lactone to the imidazolium cation, and the presence of sugar moiety has been illustrated to reduce the toxicity of the mentioned ionic liquids towards human erythrocytes and zebrafish embryos compared to the traditional ILs. These discoveries strengthen our approach in the design of pharmaceutically active ILs with the implementation of *N,N,N*-trimethyl-2-(((2*R*,3*R*,4*S*,5*S*,6*R*)-3,4,5-trihydroxy-6-(hydroxymethyl)tetrahydro-2H-pyran-2-yl)oxy)ethan-1-aminium instead of 1-Butyl-3-methylimidazolium as the cation^41^. So far, many applications of ionic liquids containing cations or anions based on carbohydrates have been investigated in the course of known chemical reactions^42^. For example, the presence of glucose-based ionic liquids has been associated with an increase in the rate of Diels–Alder reactions^43^. Alternatively, the d-glucuronate anion can be used with quaternary ammonium cations such as tetrabutylammonium to assist the selective hydrogenation of cyclooctadiene^44^.

This study reports the computed binding energies between the cation and anion (obtained at the B3LYP/6-311++G(d, p) level^45^) for our designed pharmaceutically active ionic liquids in which the cationic part is based on *N,N,N*-trimethyl-2-(((2*R*,3*R*,4*S*,5*S*,6*R*)-3,4,5-trihydroxy-6-(hydroxymethyl)tetrahydro-2H-pyran-2-yl)oxy)ethan-1-aminium. These binding energies were compared with those of the pharmaceutically active ionic liquids in which the cationic part is based on imidazolium. The related parameters of binding energies of these ILs were also calculated via molecular dynamic simulation using GROMACS 5.2.^46^. In this study, QM stands for quantum mechanics and refers to the calculations performed at the B3LYP/6-311++G (d, p) level. Moreover, MD stands for molecular dynamics and denotes molecular dynamics simulations.

## Computational methods

The initial conformer search was performed at the relative energy range of 0–10 kcal mol^−1^ to obtain the most stable structures of ILs, employing the MMFF force field (molecular mechanics)^45^. The 6-311++G(d,p) basis set was applied to optimize the geometries and frequency calculations for most newly designed structures and traditional ILs. This basis set contains the diffuse functions essential to illustrate and analyze the hydrogen bonds in the newly designed ILs and the traditional ILs^47^.

The absence of imaginary frequencies illustrated that the optimized structures were actual minima. Equations (1) and (2) were used to calculate the interaction energy (ΔE_int_) and cohesive energy of the crystals (CEC),

1 $${\Delta E}_{int }=E(ion \, pair) -E(cation)-E(anion)$$

2 $${\Delta E}_{CEC}=-{\Delta E}_{int}$$

Furthermore, to compare the QM and MD results, we applied MD simulations to calculate the binding energies of the ILs of this study using the GROMACS 5.2^46^. The topology files for cations and anions of ILs were constructed based on the Gromos 43 a1 force field using the PRODRG 2. x online server. Since the cation’s structure used in our suggested pharmaceutically active ILs is based on biomolecules^48^, we used the Gromos force field because its parameters have been successfully implemented in the simulations of biomolecules.

## Results and discussion

### Geometrical analysis of pharmaceutically active ionic liquids based on 1-butyl-3-methylimidazolium

The compounds forming hydrogen bonds with their targets are very significant in drug discovery. For example, the molecule with H-bond donors and acceptors can show various behaviors depending on the environment. The closed form of this molecule, as the drug, is expected to be more lipophilic and has better permeability through the cell membrane^49^. Furthermore, the intramolecular hydrogen bonds are likely to restrict the number of molecular conformations of ligands, which can be crucial variables in the interactions between ligands and their receptors^49^.

**Figure 1:**
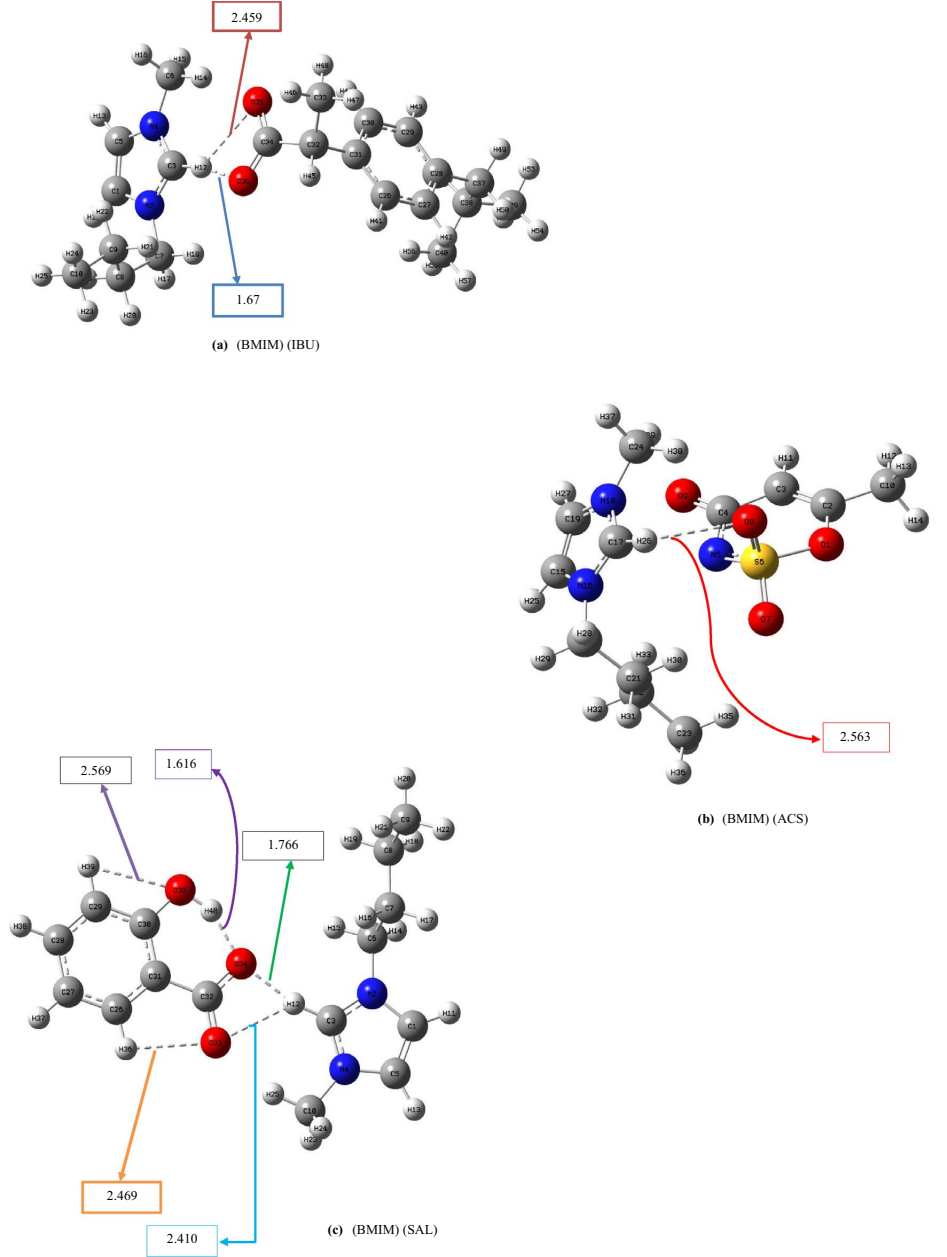

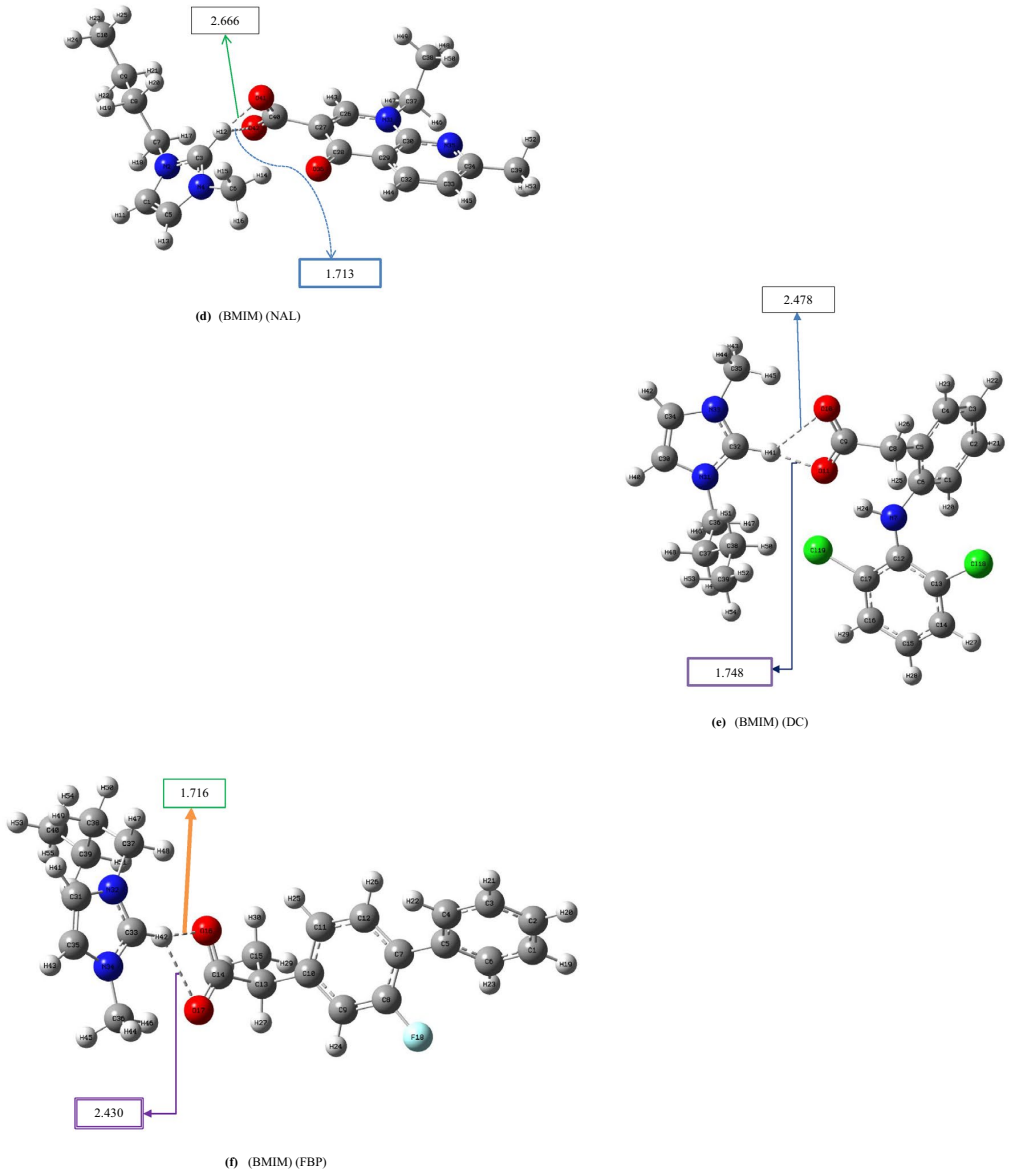

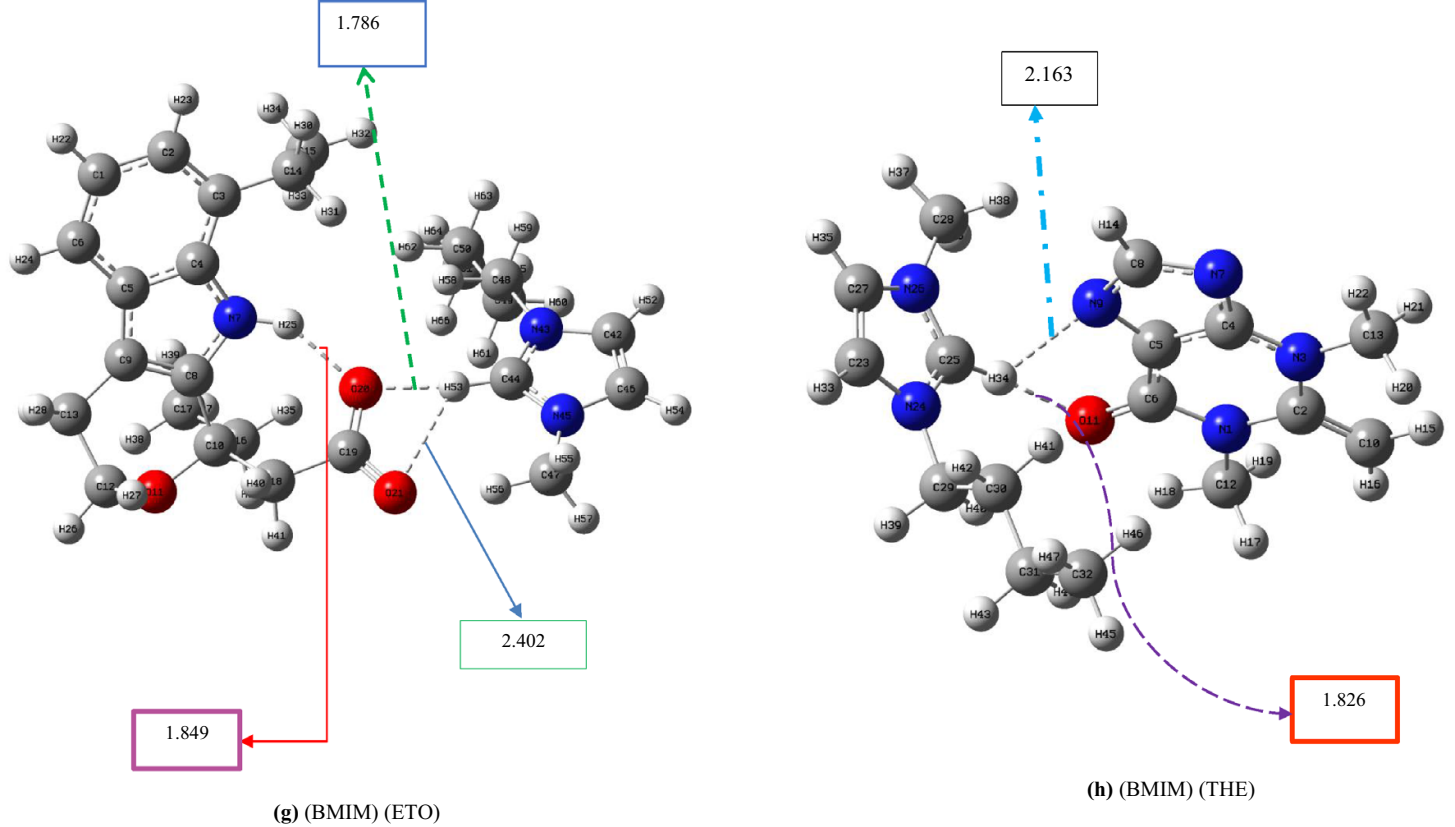
Optimized geometries of the lowest-energy conformers of BMIM-based pharmaceautically active ILs calculated at the B3LYP/6-311++G(d,p) level.

We have reported the influence of intramolecular hydrogen bonding to enhance the acidity of polyols^50^ and the binding energy between the cation and anion of ILs^51^. To explore the effects of intramolecular hydrogen-bonding networks on the binding energies between the cation and anion of the ILs, we designed a new class of pharmaceutically active ionic liquids based on *N,N,N*-trimethyl-2-(((2*R*,3*R*,4*S*,5*S*,6*R*)-3,4,5-trihydroxy-6-(hydroxymethyl)tetrahydro-2H-pyran-2-yl)oxy)ethan-1-aminium. The binding energies of the traditional pharmaceutically active ionic liquids, which are based on 1-butyl-3-methylimidazolium, were compared with those of the designed ILs. We believe that our newly designed ILs can have improved drug release rates as the binding energies of these novel ILs are expected to be lower than those of the known ILs. In this study, first, we considered the traditional pharmaceutical ILs based on 1-butyl-3-methylimidazolium. The optimized structures of the anion and cation of the known pharmaceutical ILs are illustrated in Fig. 1. Figure 1 shows the lowest-energy conformers of known pharmaceutically active ionic liquids based on 1-butyl-3-methylimidazolium; other less stable conformers can be seen in Figs. S1–S8 in Supporting Information. Table 1 includes the binding energy values between the anion and cation of ILs obtained at the B3LYP/6-311++G (d, p) level and codes assigned for each structure. For example, (BMIM) (IBU) stands for 1-Butyl-3-methylimidazolium-2-(4-isobutylphenyl) propanoate. Moreover, to see the effects of the length of the alkyl chain attached to the imidazolium ring on the inter- and intramolecular hydrogen bonds in the ionic liquids and the interactions between cation and anions, we performed some calculations for 3-methyl-1-octyl-1H-imidazol-3-ium- 2-hydroxybenzoate (M8OI) (SAL) (Table 1, entries 18 and 19). The optimized structure of (M8OI) (SAL) ionic liquid and its other conformers can be seen in Figures S9 and S10. Also, some of the structural features of (M8OI) (SAL) can be found in Table S1.

**Table 1:** ΔE_int_ and ΔE_CEC_ (kcal.mol^-1^) for the ionic liquids based on BMIM, calculated at the B3LYP/6-311++G(d,p) level.

Entry	Name of structures	Structure	Name of drug	Code of structure	E_SCF_ (Hartree per particle)	ΔE_int_ (kcal mol^−1^)	ΔE_CEC_ (kcal mol^−1^)
1	1-Butyl-3-methylimidazolium			(BMIM)	−423.0516		
2	2-(4-isobutylphenyl) propanoate		Ibuprofen (anion)	(IBU)	−656.0566		
3	6-methyl-4-oxo-4H-1,2,3-oxathiazin-3-ide 2,2-dioxide		Acesulfame (anion)	(ACS)	−908.7562		
4	2-hydroxybenzoate		Salicylate (anion)	(SAL)	−495.5596		
5	1-ethyl-7-methyl-4-oxo-1,4-dihydro-1,8-naphthyridine-3-carboxylate		Nalidixic acid anion	(NAL)	−799.1611		
6	2-(2-((2,6-dichlorophenyl) amino) phenyl) acetate		Diclofenac (anion)	(DC)	−1665.2288		
7	2-(2-fluoro-[1,1'-biphenyl]-4-yl) propanoate		Flurbiprofen (anion)	(FBP)	−829.1811		
8	2-(1,8-diethyl-1,3,4,9-tetrahydropyrano[3,4-b] indol-1-yl) acetate		Etodolac	(ETO)	−940.2838		
9	1,3-dimethyl-2-methylene-6-oxo-1,2,3,6-tetrahydropurin-7-ide		Theophylline (anion)	THE	−604.5418		
10	1-Butyl-3-methylimidazolium -2-(4-isobutylphenyl) propanoate		Ibuprofen (anion)	(BMIM) (IBU)	−1079.2548	−91.9	91.9
11	1-Butyl-3-methylimidazolium-6-methyl-4-oxo-4H-1,2,3-oxathiazin-3-ide 2,2-dioxide		Acesulfame (anion)	(BMIM)(ACS)	−1331.9332	−78.7	78.7
12	1-Butyl-3-methylimidazolium-2-hydroxybenzoate		Salicylate (anion)	(BMIM) (SAL)	−918.7451	−84.0	84.0
13	1-Butyl-3-methylimidazolium-1-ethyl-7-methyl-4-oxo-1,4-dihydro-1,8-naphthyridine-3-carboxylate		Nalidixic acid anion	(BMIM) (NAL)	−1222.3636	−94.7	94.7
14	1-Butyl-3-methylimidazolium- 2-(2-((2,6-dichlorophenyl) amino) phenyl) acetate		Diclofenac(anion)	(BMIM) (DC)	−2088.4174	−85.9	85.9
15	1-Butyl-3-methylimidazolium-2-(2-fluoro-[1,1'-biphenyl]-4-yl) propanoate		Flurbiprofen (anion)	(BMIM) (FBP)	−1252.3731	−88.2	88.2
16	1-Butyl-3-methylimidazolium-2-(1,8-diethyl-1,3,4,9-tetrahydropyrano[3,4-b] indol-1-yl) acetate		Etodolac	(BMIM) (ETO)	−1363.4652	−81.5	81.5
17	1-Butyl-3-methylimidazolium-1,3-dimethyl-2-methylene-6-oxo-1,2,3,6-tetrahydropurin-7-ide		Theophylline (anion)	(BMIM) (THE)	−1027.7263	−83.4	83.4
18^a^	3-methyl-1-octyl-1H-imidazol-3-ium			(M8OI)	−580.2311		
19^a^	3-methyl-1-octyl-1H-imidazol-3-ium- 2-hydroxybenzoate		Salicylate (anion)	(M8OI) (SAL)	−1075.9226	−82.8	82.8

^(a)^ Entries 18 and 19 were added to Table 1, to compare the effects of the alkyl chain length in the cation structure of IL on the formation of intramolecular and intermolecular hydrogen bonds.

Based on Fig. 1c, the average distance between cation and anion in (BMIM) (SAL) is 2.088 Å; whereas, in (M8OI) (SAL), the similar distance is 2.108 Å (Figure S9). These observations were in agreement with the calculated cohesive energies (ΔE_CEC_) for (BMIM) (SAL) and (M8OI) (SAL). As the distance between cation and anion increases, cohesive energy for ionic liquid decreases ( ΔE_CEC_ of (M8OI) (SAL) < ΔE_CEC_ of (BMIM) (SAL)). The intramolecular hydrogen bond distances in the salicylate structures in both ionic liquids were the same (1.616 Å) (Table S1).

As seen in Figs. 1a–h, the average length of the intermolecular distances varies between 2.065 and 2.563 Å. As the intermolecular distances between cation and anion increase, the cohesive energy of the ionic liquids decreases (see Fig. 1 and Table 1). The distances illustrated in Fig. 1 have been summarised in Table S1.

### Geometric analysis of the newly designed pharmaceutical ionic liquids based on *N*-[2-(d-glucopyranosyl) ethyl]-*N, N, N*-trimethylammonium

**Figure 2:**
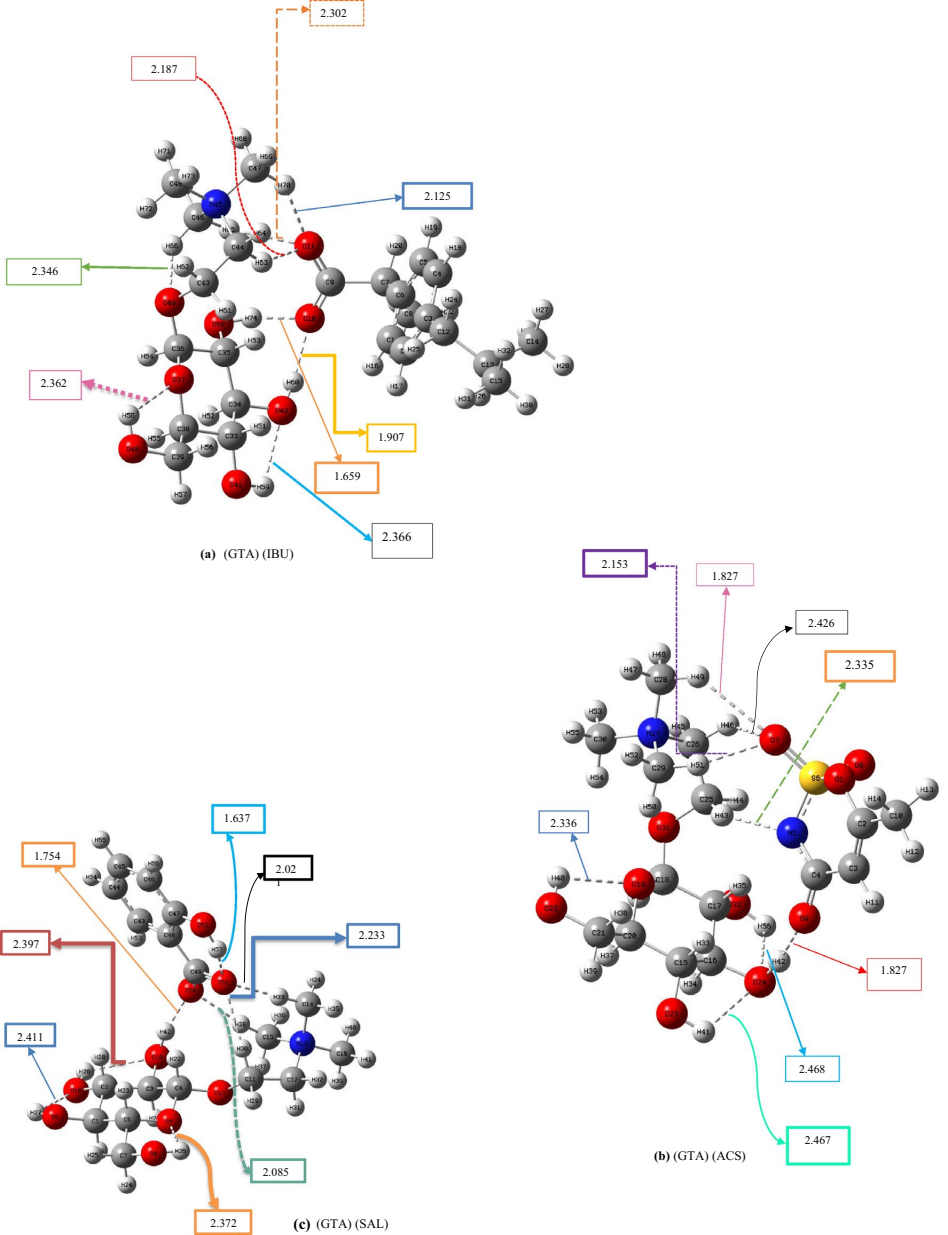

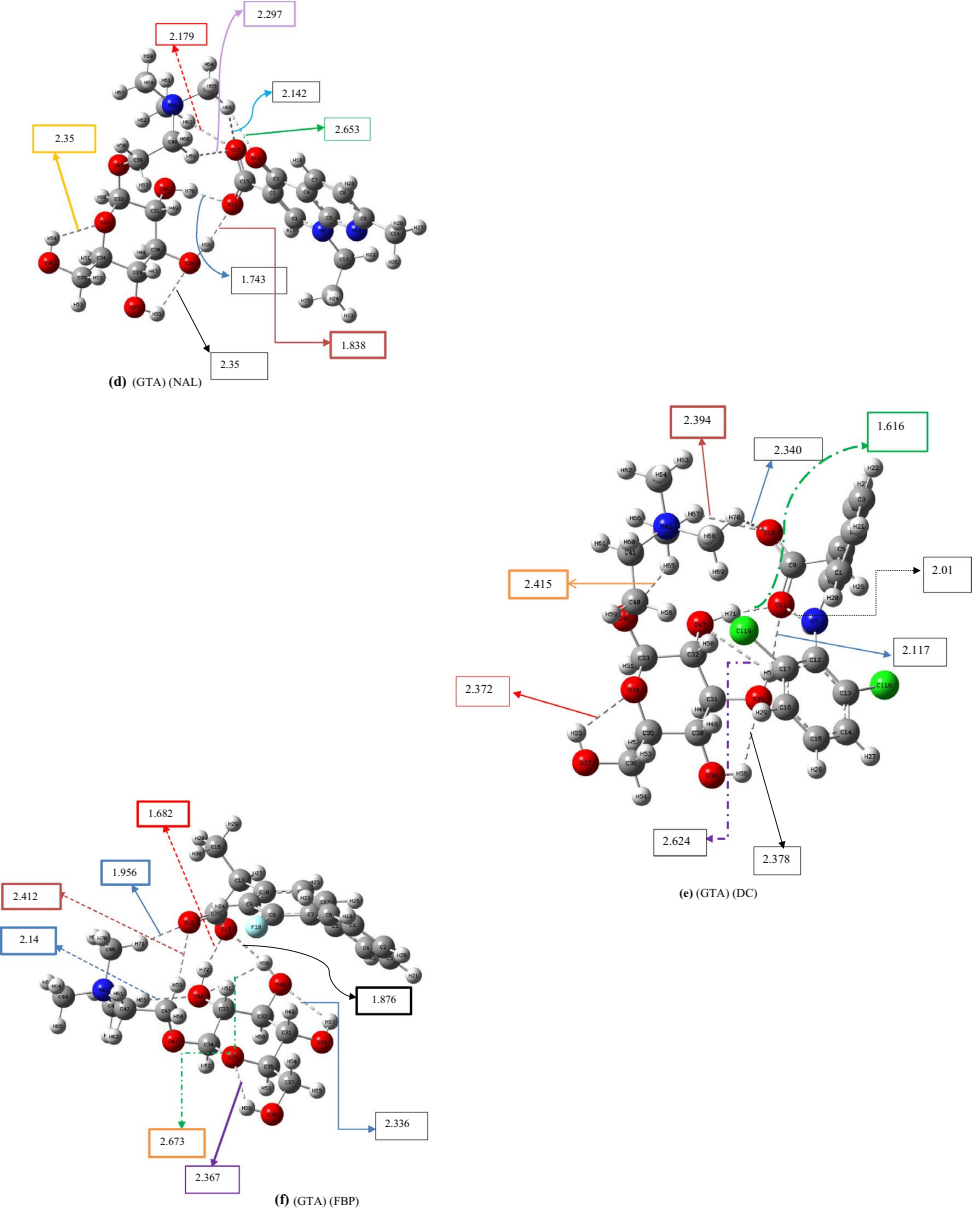

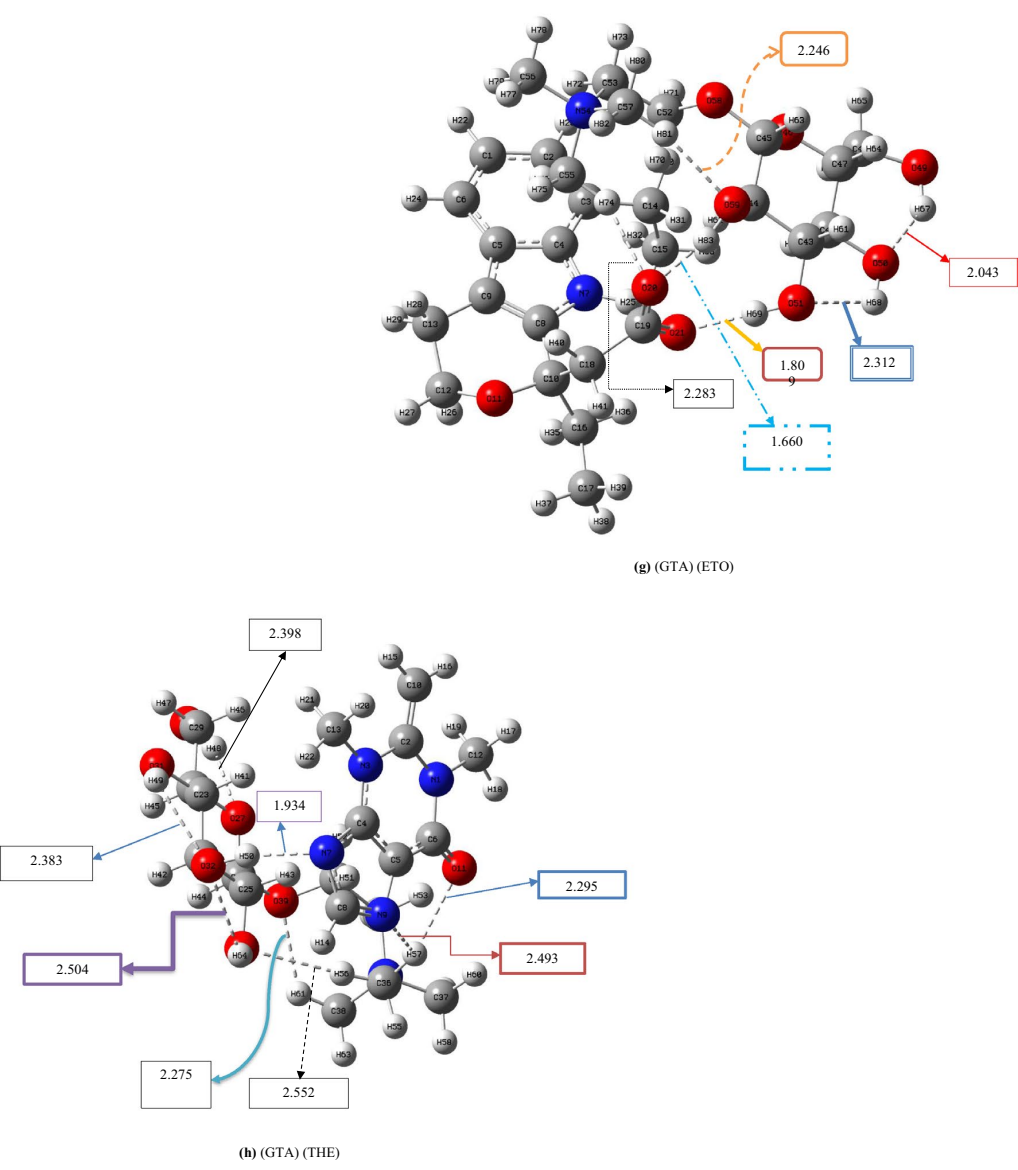
Optimized geometries of the lowest-energy conformers of *N,N,N*-trimethyl-2-(((2*R*,3*R*,4*S*,5*S*,6*R*)-3,4,5-trihydroxy-6-(hydroxymethyl)tetrahydro-2H-pyran-2-yl)oxy)ethan-1-aminium-based ILs including: (**a)** (GTA) (IBU), (**b)** (GTA) (ACS), (**c)** (GTA)(SAL), (**d)** (GTA)(NAL), (**e)** (GTA)(DC), (**f)** (GTA)(FBP), (**g)** (GTA)(ETO) and (**h)** (GTA)(THE)at the B3LYP/6-311++G(d,p) level.

In this section, the cations of the pharmaceutically active ionic liquids based on 1-butyl-3-methyl imidazolium ionic liquids were replaced by *N,N,N*-trimethyl-2-(((2*R*,3*R*,4*S*,5*S*,6*R*)-3,4,5-trihydroxy-6-(hydroxymethyl)tetrahydro-2H-pyran-2-yl)oxy)ethan-1-aminium to explore the effect of the intramolecular hydrogen-bonding network on the binding energy between the cation and anion of the IL. The codes and ΔE_CEC_ values of these newly designed ILs (calculated at the 6-311++G** level of theory) and their nomenclatures are listed in Table 2. The optimized structures of their lowest-energy conformers, including (GTA) (IBU), (GTA) (ACS), (GTA) (SAL), (GTA) (NAL), (GTA) (DC), (GTA) (FBP), (GTA) (ETO) and (GTA) (THE) are illustrated in Fig. 2. Other conformers of these designed ILs are given in Figs. S11–S18 in Supporting Information. The distances shown in Fig. 2 are summarized in Table S2.

**Table 2:** ΔE_int_ and ΔE_CEC_ (kcal mol^−1^) of ionic liquids based on *N,N,N*-trimethyl-2-(((2*R*,3*R*,4*S*,5*S*,6*R*)-3,4,5-trihydroxy-6- (hydroxymethyl)tetrahydro-2H-pyran-2-yl)oxy)ethan-1-aminium (GTA), calculated at the B3LYP/6-311++G(d,p) level.

Entry	Name of structures	Structure	Name of drugs	Codes of structures	E_SCF_ (Hartree per particle)	ΔE_int_ (kcal mol^−1^)	ΔE_CEC_ (kcal mol^−1^)
1	*N,N,N*-trimethyl-2-(((2*R*,3*R*,4*S*,5*S*,6*R*)-3,4,5-trihydroxy-6-(hydroxymethyl)tetrahydro-2H-pyran-2-yl)oxy)ethan-1-aminium			(GTA)	−939.3519		
2	*N,N,N*-trimethyl-2-(((2*R*,3*R*,4*S*,5*S*,6*R*)-3,4,5-trihydroxy-6-(hydroxymethyl)tetrahydro-2H-pyran-2-yl)oxy)ethan-1-aminium -2-(4-isobutylphenyl) propanoate		Ibuprofen (anion)	(GTA) (IBU)	−1595.5586	−94.2	94.2
3	*N,N,N*-trimethyl-2-(((2*R*,3*R*,4*S*,5*S*,6*R*)-3,4,5-trihydroxy-6-(hydroxymethyl)tetrahydro-2H-pyran-2-yl)oxy)ethan-1-aminium-6-methyl-4-oxo-4H-1,2,3-oxathiazin-3-ide 2,2-dioxide		Acesulfame (anion)	(GTA) (ACS)	−1848.2346	−79.4	79.4
4	*N,N,N*-trimethyl-2-(((2*R*,3*R*,4*S*,5*S*,6*R*)-3,4,5-trihydroxy-6-(hydroxymethyl)tetrahydro-2H-pyran-2-yl)oxy)ethan-1-aminium -2-hydroxybenzoate		Salicylate (anion)	(GTA) (SAL)	−1435.0439	−83.1	83.1
5	*N,N,N*-trimethyl-2-(((2*R*,3*R*,4*S*,5*S*,6*R*)-3,4,5-trihydroxy-6-(hydroxymethyl)tetrahydro-2H-pyran-2-yl)oxy)ethan-1-aminium -1-ethyl-7-methyl-4-oxo-1,4-dihydro-1,8-naphthyridine-3-carboxylate		Nalidixic acid anion	(GTA) (NAL)	−1738.6722	−99.9	99.9
6	*N,N,N*-trimethyl-2-(((2*R*,3*R*,4*S*,5*S*,6*R*)-3,4,5-trihydroxy-6-(hydroxymethyl)tetrahydro-2H-pyran-2-yl)oxy)ethan-1-aminium-2-(2-((2,6-dichlorophenyl) amino) phenyl) acetate		Diclofenac (anion)	(GTA) (DC)	−2604.7083	−80.1	80.1
7	*N,N,N*-trimethyl-2-(((2*R*,3*R*,4*S*,5*S*,6*R*)-3,4,5-trihydroxy-6-(hydroxymethyl)tetrahydro-2H-pyran-2-yl)oxy)ethan-1-aminium -2-(2-fluoro-[1,1'-biphenyl]-4-yl) propanoate		Flurbiprofen (anion)	(GTA) (FBP)	−1768.6741	−88.6	88.6
8	*N,N,N*-trimethyl-2-(((2*R*,3*R*,4*S*,5*S*,6*R*)-3,4,5-trihydroxy-6-(hydroxymethyl)tetrahydro-2H-pyran-2-yl)oxy)ethan-1-aminium -2-(1,8-diethyl-1,3,4,9-tetrahydropyrano[3,4-b] indol-1-yl) acetate		Etodolac	(GTA) (ETO)	−1879.7635 −1879.763502	−80.2	80.2
9	*N,N,N*-trimethyl-2-(((2*R*,3*R*,4*S*,5*S*,6*R*)-3,4,5-trihydroxy-6-(hydroxymethyl)tetrahydro-2H-pyran-2-yl)oxy)ethan-1-aminium -1,3-dimethyl-2-methylene-6-oxo-1,2,3,6-tetrahydropurin-7-ide		Theophylline (anion)	(GTA) (THE)	−1544.0196	−79.0	79.0

**Figure 3:**
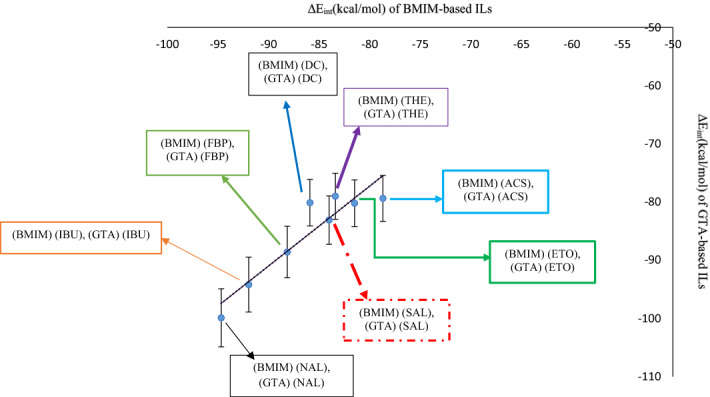
ΔE_int_ values (kcal mol^−1^) of GTA-based ILs versus ΔE_int_ values of BMIM-based Ils (error bar% = 5).

The ΔE_int_ values of GTA-based ILs were plotted against ΔE_int_ values of 1-butyl-3-methylimidazolium (Fig. 3, $y=1.372x+32.471$, R^2^ = 0.8647). This plot indicates that GTA can be as effective as BMIM in binding with the pharmaceutically active anion of the IL. This plot confirms that GTA can be a suitable replacement for 1-butyl-3-methylimidazolium in drug delivery systems.

### Calculating the ΔE_int_ of the ILs by use of molecular dynamics simulations

To compare the ΔE_int_ values obtained by B3LYP/6-311++G(d,p) level with those from molecular dynamics methodology, the MD simulations were employed to calculate the binding energies of both groups of pharmaceutical ionic liquids (ΔG_binding_), including the known and designed ILs. 10 ionic liquids were chosen randomly and studied by the GROMACS 5.2^46^. The topology files for the chosen ILs were prepared according to the Gromos 43 a1 force field using the PRODRG 2.x online server. To measure ΔG_binding_ between cations and anions in each of the chosen ILs, cubic boxes were considered with faces at least 1 to 2 nm far from the closest atom of our systems. MD simulation for the minimization of each system was conducted in three steps. The minimization step was initiated by applying the steepest descent method. The conjugate gradient methods were manipulated for the next step. Then, the steepest descent method with an emtol value of 100.0 kJ mol^−1^. nm^−1^ was employed for the third step. After the steps mentioned earlier, the overall systems were equilibrated in the NVT ensemble (where N, V, and T stand for the number of particles, volume, and temperature, respectively) for 500 ps (1 ps = 10^–12^ s) at 100 K. In the NVT ensemble, the temperature-coupling times were 0.1 ps. This step was followed by the NPT ensemble (where N, P, and T stand for the number of particles, pressure, and temperature). The NPT equilibration was achieved by adopting an MD integrator for 1000 ps at 298 K and 1 bar. The Berendsen algorithm was picked for both barostat and thermostat coupling algorithms in this step for all systems^52^. Within the NPT ensemble, for raising the temperature of ionic liquids from 100 to 298 K, the velocities were raised based on the Maxwell–Boltzmann distribution^53^. Subsequently, the MD production runs for all of the ILs, which were under molecular dynamics studies, were initiated for nearly 20 ns (1 ns = 10^–9^ s) at 298 K with a time step of 1 fs (1 fs = 10^–15^ s) with around twenty million steps in total. The Parrinello–Rahman barostat^54^ and Nose–Hoover thermostat algorithms^55^ were utilized in the MD simulation.

### Analysis of molecular dynamics studies on the selected BMIM-based pharmaceutically active ILs

#### 1-Butyl-3-methylimidazolium -2-(4-isobutylphenyl) propanoate (BMIM) (IBU)

**Figure 4:**
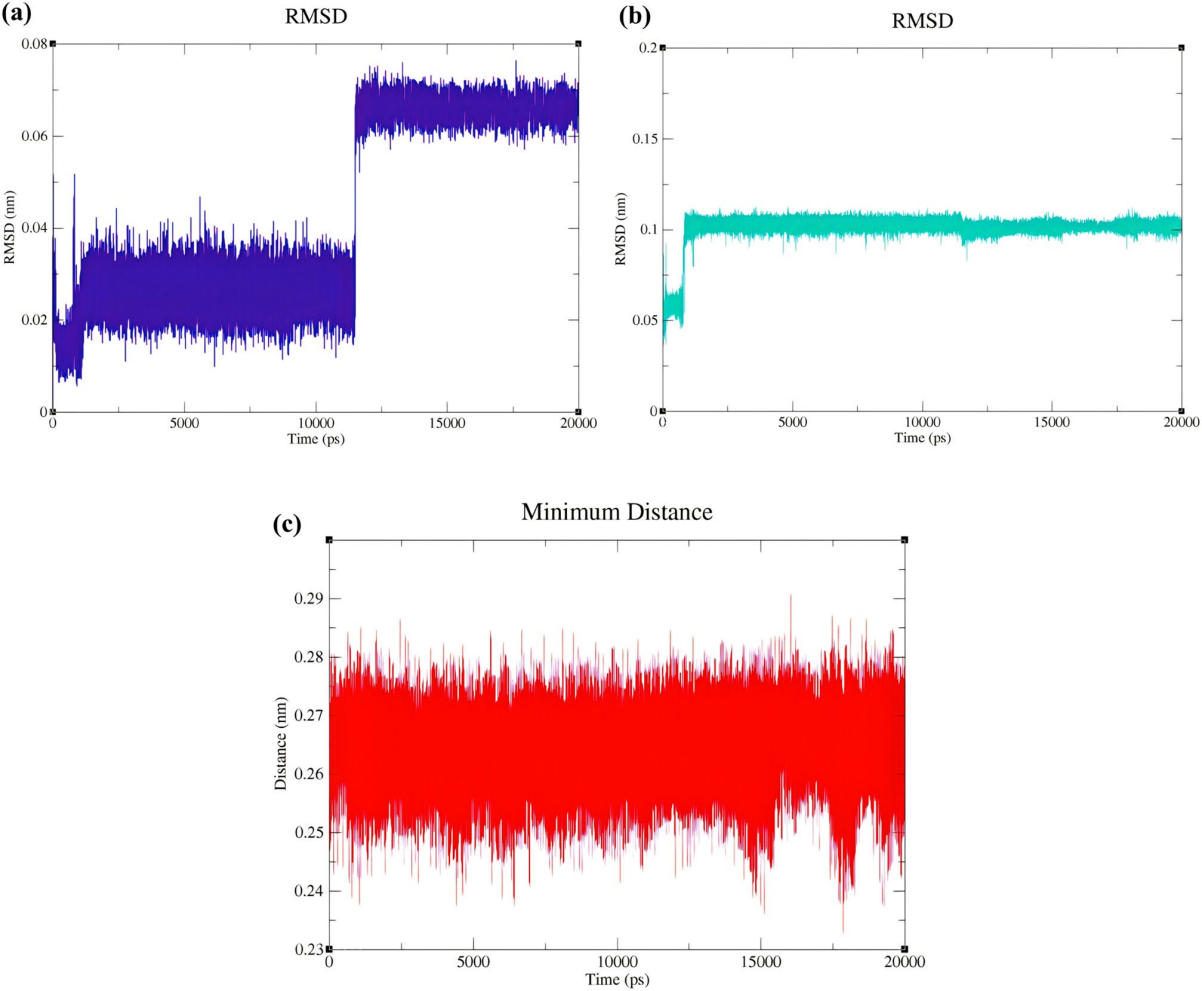
BMIM backbone RMSD **(a)** and IBU backbone RMSD **(b)** in the structure of [(BMIM) (IBU)] IL. **(c)** The plot of average of the minimum distance (nm) between BMIM and IBU against time (ps) during 20 ns of molecular dynamics simulation.

After MD simulation, the RMSD values of 1-butyl-3-methylimidazolium and 2-(4-isobutylphenyl) propanoate were evaluated with respect to the optimized geometry of (BMIM) (IBU) obtained at the B3LYP/6-311++G(d,p) level. As shown in Fig. 4a, RMSD values for 1-butyl-3-methylimidazolium during 20 ns of simulation fluctuate between 0.1 Å and 0.7 Å compared to the cation of the optimized IL (BMIM) (IBU). Moreover, RMSD values for IBU ranged from 0.5 to 1 Å (Fig. 4b) compared to the anion of the optimized IL (BMIM) (IBU). These RMSD plots indicate that the (BMIM) (IBU) IL was stable during MD simulation. RMSD values for BMIM during 20 ns of MD simulation were processed. The average RMSD value and its standard deviation for BMIM were 0.0426 nm and 0.0207 nm, respectively. Additionally, due to the similar measurements for RMSD values of IBU, the average RMSD value and its standard deviation value for anion were 0.101 nm and 0.00922 nm, respectively.

Also, the average minimum distance between the cation and anion of the (BMIM) (IBU) was calculated during 20 ns of MD simulation and found to be between 2.4 and 2.85 Å (Fig. 4c). Furthermore, the average value and the standard deviation of minimum distances between cation and anion during MD simulation were found to be 2.7 Å and 0.064 Å.

Comparison of this average minimum distance between cation and anion obtained from MD with that obtained from QM (Fig. 1a, ($\frac{1.671+2.459}{2}=2.065$Å) indicates that these MD and QM values are comparable. The higher MD average minimum distance can be attributed to the dynamics of the cation and anion of the IL.

The various energy terms for the ILs calculated by the gmmpbsa package are listed in Table 3. As seen in Table 3, the binding energy between the cation BMIM and the anion IBU obtained by the molecular dynamics method and MM/PBSA calculations is −275.0 kJ. mol^−1^ (−65.7 kcal.mol^−1^). According to Table 1, the ΔE_int_ between the cation BMIM and the anion IBU calculated at the B3LYP/6-311++G(d,p) level is −91.9 kcal mol^−1^. The difference between parameters calculated by MD simulation and QM studies is due to the dynamics of the IL and the contribution of its various conformers.

**Table 3:** Various energy terms obtained through MM/PBSA calculations of ΔE_int_ of the GTA-based ILs and the BMIM-based ILs.

					MM/PBSA					
Entry	Name of structures	Structure	Name of drugs	Codes of structures	van der Waals energy (kJ mol^−1^)	Electrostatic energy (kJ mol^−1^)	Polar solvation energy (kJ mol^−1^)	Non-polar solvation energy (kJ mol^−1^)	Binding energy (kJ mol^−1^)	Binding energy (kcal mol^−1^)
1	1-Butyl-3-methylimidazolium -2-(4-isobutylphenyl) propanoate		Ibuprofen (anion)	(BMIM) (IBU)	−6.475	−343.071	78.327	−3.803	−275.022 ± 35.868	−65.73
2	1-Butyl-3-methylimidazolium-2-hydroxybenzoate		Salicylate (anion)	(BMIM) (SAL)	15.464	−749.614	302.855	−2.444	−433.740 ± 79.001	−103.66
3	1-Butyl-3-methylimidazolium-2-(2-fluoro-[1,1'-biphenyl]-4-yl) propanoate		Flurbiprofen (anion)	(BMIM) (FBP)	−6.337	−305.353	27.759	−2.086	−286.018 ± 7.411	−68.36
4	1-Butyl-3-methylimidazolium-1-ethyl-7-methyl-4-oxo-1,4-dihydro-1,8-naphthyridine-3-carboxylate		Nalidixic acid anion	(BMIM) (NAL)	-3.318	-400.089	34.169	−1.088	−370.327 ± 70.063	−88.4
5	1-Butyl-3-methylimidazolium-6-methyl-4-oxo-4H-1,2,3-oxathiazin-3-ide 2,2-dioxide		Acesulfame (anion)	(BMIM) (ACS)	−8.420	−147.108	118.796	−2.156	−38.889 ** ± **16.212 kJ/mol	−9.29
6	N,N,N-trimethyl-2-(((2R,3R,4S,5S,6R)-3,4,5-trihydroxy-6-(hydroxymethyl)tetrahydro-2H-pyran-2-yl)oxy)ethan-1-aminium -2-(4-isobutylphenyl) propanoate		Ibuprofen (anion)	(GTA) (IBU)	−18.024	−484.619	258.622	−4.889	−248.910 ± 7.535	−59.3
7	N,N,N-trimethyl-2-(((2R,3R,4S,5S,6R)-3,4,5-trihydroxy-6-(hydroxymethyl)tetrahydro-2H-pyran-2-yl)oxy)ethan-1-aminium—6-methyl-4-oxo-4H-1,2,3-oxathiazin-3-ide 2,2-dioxide		Acesulfame (anion)	(GTA) (ACS)	−6.711	−20.513	11.501	−1.133	−16.856 ** ± **12.316	-4.03
8	N,N,N-trimethyl-2-(((2R,3R,4S,5S,6R)-3,4,5-trihydroxy-6-(hydroxymethyl)tetrahydro-2H-pyran-2-yl)oxy)ethan-1-aminium -2-hydroxybenzoate		Salicylate (anion)	(GTA) (SAL)	−14.176	−480.735	249.651	−4.603	−249.863 ± 4.575	−59.72
9	N,N,N-trimethyl-2-(((2R,3R,4S,5S,6R)-3,4,5-trihydroxy-6-(hydroxymethyl)tetrahydro-2H-pyran-2-yl)oxy)ethan-1-aminium -1-ethyl-7-methyl-4-oxo-1,4-dihydro-1,8-naphthyridine-3-carboxylate		Nalidixic acid anion	(GTA) (NAL)	−28.101	−670.360	408.664	−5.480	−295.276 ± 13.094	−70.57
10	N,N,N-trimethyl-2-(((2R,3R,4S,5S,6R)-3,4,5-trihydroxy-6-(hydroxymethyl)tetrahydro-2H-pyran-2-yl)oxy)ethan-1-aminium -2-(2-fluoro-[1,1'-biphenyl]-4-yl) propanoate		Flurbiprofen (anion)	(GTA) (FBP)	−24.825	−455.774	245.841	−5.063	−239.821 ± 8.333	−57.32

### Analysis of molecular dynamics studies on the selected pharmaceutically active IL (GTA) (IBU)

**Figure 5:**
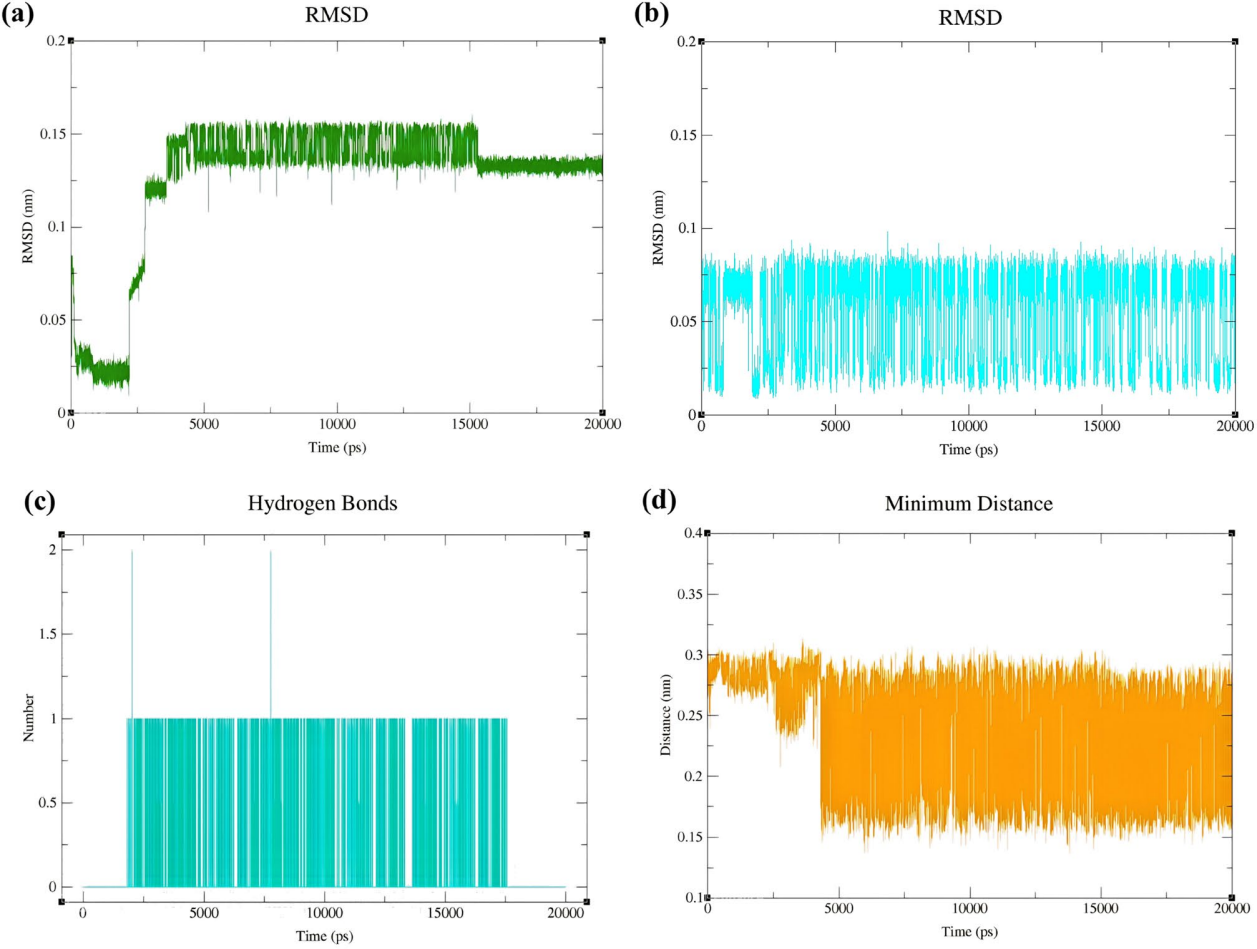
GTA backbone RMSD **(a)** and IBU backbone RMSD **(b)** in the structure of [(GTA) (IBU)] IL. **(c)** Intermolecular hydrogen bonds between GTA and IBU during 20 ns of MD simulation, **(d)** the patterns of the changes in the distance values between GTA and IBU during 20 ns of MD simulation.

The RMSD values of the cation and anion of [(GTA) (IBU)] (refer to Table 2 for nomenclature) were investigated with respect to the optimized geometry of the IL (GTA) (IBU) obtained by QM calculations at the B3LYP/6-311++G(d,p) level. As shown in Fig. 5a, the RMSD values of the cation GTA during 20 ns of simulation fluctuate between 0.025 and 0.15 nm during 20 ns of MD simulation. The corresponding average and the standard deviation of the RMSD values of GTA were found to be 0.125 nm and 0.0377 nm, respectively.

Moreover, RMSD values of the anion IBU ranged from 0.0125 to 0.075 nm (Fig. 5b) with respect to the anion of the optimized IL (GTA) (IBU). The average value and the standard deviation of the RMSD values of IBU were 0.0536 nm and 0.0242 nm, respectively. These RMSD plots indicate that the [(GTA) (IBU)] IL was stable during MD simulation.

Moreover, according to Fig. 2a, resulting from QM calculations, the number of intramolecular hydrogen bonds in the cation GTA was predicted to be 3. In contrast, the number of intramolecular hydrogen bonds in the cation GTA fluctuates between 0 and 2 during 20 ns of the MD simulation (refer to Fig. 5c). In Fig. 5d, the average and the standard deviation of the minimum distances between cation (GTA) and anion (IBU) during 20 ns of molecular dynamics simulation can be displayed as 2.38  ± 0.43 Å; whereas, in Fig. 2a, this average between cation and anion is 2.036 Å. The higher MD distance is due to the dynamics of the ILs.

Furthermore, the average intermolecular distances of the rest of the designed ILs including (BMIM) (IBU), (BMIM) (SAL), (BMIM) (FBP), (BMIM) (NAL), (BMIM) (ACS), (GTA) (IBU), (GTA) (SAL), (GTA) (FBP), (GTA) (NAL), and (GTA) (ACS), which were measured in QM and MD studies, are listed in Table S3.

### Finding a link between QM results and MD results

The MD ΔG_binding_ values between the cations and anions of both groups of ionic liquids, including BMIM-based ILs and GTA-based ILs, were calculated by considering the Molecular Mechanics/Poisson–Boltzmann Surface Area (MM/PBSA) method^56^ during the first ten ns of the MD trajectory by 50 snapshots. The binding energies between cations and anions were obtained by the molecular dynamic method and MM/PBSA calculations. The various energy terms calculated by the gmmpbsa package are listed in Table 3.

Additionally, we computed the precise values of ΔG_binding_ (kcal mol^−1^) for all of the ionic liquids in Table 3 at the B3LYP/6-311++G(d,p) level. We applied Eq. (3) to obtain ΔG_binding_ for the ILs.

3 $${\Delta G}_{binding }=G(ion \, pair) -G(cation)-G(anion)$$

As seen in Table S4, the molecular dynamics simulations indicate that most BMIM-based ILs have more negative ΔG_binding_ than GTA-based ILs. Whereas the results from QM studies show the opposite trend (ΔG_binding_ of GTA-based ILs < ΔG_binding_ of BMIM-based ILs). The difference between the MD and QM results can be attribiuted to more conformational changes of the GTA cation during MD studies compared to the BMIM cation. 1-Butyl-3-methylimidazolium in BMIM has an aromatic imidazolium ring that has less flexibility than D-glucopyranosyl in GTA.

**Figure 6:**
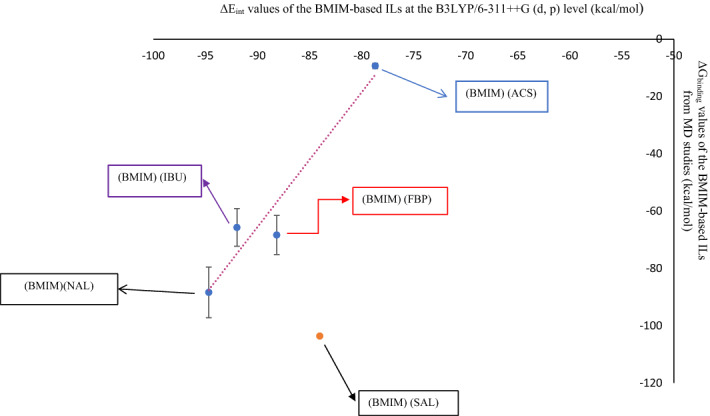
Correlation between ΔG_binding_ (kcal mol^−1^) from 20 ns of MD simulation and ΔE_int_ (kcal mol^−1^) from QM studies in: (BMIM) (ACS), (BMIM) (FBP), (BMIM) (IBU), and (BMIM)(NAL), (error bar % = 10).

Also, a comparison of Table 3 and Table S4 indicates that the calculated ΔG_binding_ values between the cations and anions of some of the BMIM-based ILs, such as (BMIM) (NAL), are similar in both QM and MD studies (compare Table 3, entry 4 with Table S4, entry 3). To find the relationship between the binding energy values of BMIM-based ILs obtained by QM and MD methods, their MD ΔG_binding_ values (during the 20 ns of MD simulation) were plotted against their QM ΔE_int_ values (Fig. 6), resulting in the linear equation;$y=4.702x+357.57,$ R^2^ = 0.9352.

**Figure 7:**
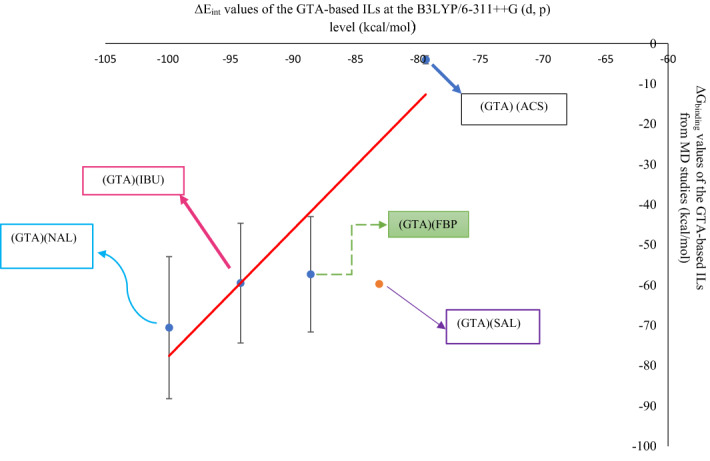
Correlation between ΔG_binding_ (kcal mol^−1^) from 20 ns of MD simulation and ΔE_int_ (kcal mol^−1^) from QM studies in: (GTA) (ACS), (GTA) (FBP), (GTA) (IBU), and (GTA)(NAL), (error bar % = 25).

For the GTA-based ILs, the plot of the QM ΔE_int_ values versus the MD ΔG_binding_ values (during the 20 ns of MD simulation) leads to the linear equation $y=3.163 x+238.42,$ R^2^ = 0.8627 (Fig. 7).

**Figure 8:**
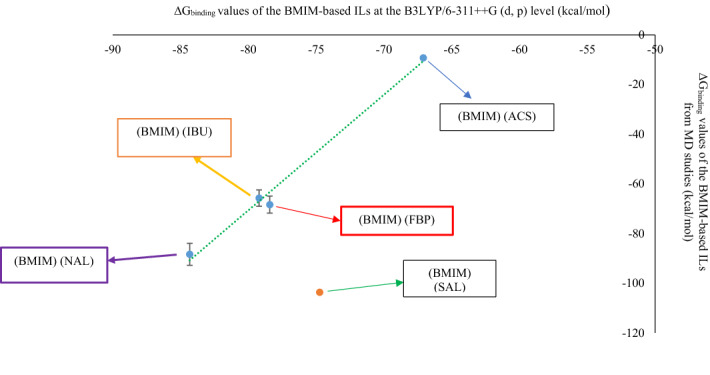
The relationship between the QM ΔG_binding_ (kcal mol^−1^) and MD ΔG_binding_ (kcal mol^−1^) for following BMIM-based ILs: (BMIM) (ACS), (BMIM) (FBP), (BMIM) (IBU), and (BMIM) (NAL), (error bar % = 5).

Plotting the MD ΔG_binding_ values of BMIM-based ILs (during the 20 ns of MD simulation) against their QM ΔG_binding_ values leads to the linear equation (Fig. 8, $y=4.654x+301.64,$ R^2^ = 0.9901).

**Figure 9:**
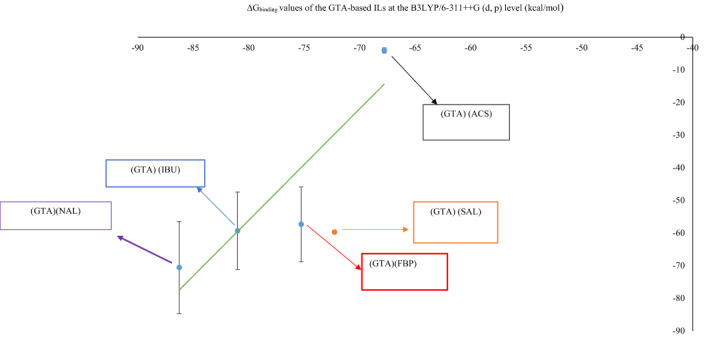
Correlation between ΔG_binding_ (kcal mol^−1^) from 20 ns of MD simulation and ΔE_int_ (kcal mol^−1^) from QM studies in: (GTA) (ACS), (GTA) (FBP), (GTA) (IBU), and (GTA)(NAL), (error bar % = 20).

For the GTA-based ILs, the linear equation y = 3.417 x + 217.29, R^2^ = 0.8283 is obtained from the plot of the QM ΔG_binding_ values against the MD ΔG_binding_ values (during the 20 ns of MD simulation) (Fig. 9).

### Comparison of the solvation energies obtained from QM and MD calculations

The MD solvation energy outputs of MM-PB/SA calculation for our systems were calculated using the Eq. 4 by adding Polar solvation energy (kJ.mol^-1^) and Non-polar solvation energy (kJ.mol^-1^) given in Table (3)^57,58^. To compare the QM and MD solvation energies, we chose the ionic liquids shown in Table 3 and calculated their QM solvation energies using the Eq. 5.

4 $${{G}_{\mathrm{solvation }}=G}_{\mathrm{polar \; solvation }}+{G}_{\mathrm{nonpolar \; solvation}}$$

5 $${\mathrm{\Delta G}}_{\mathrm{solvation}}^{\mathrm{binding}} ={G}_{\mathrm{solvation \; of \; ILs \; as \; ion \; pairs }}-({G}_{\mathrm{solvation \; of \; cation }}+{G}_{\mathrm{solvation \; of \; anion }})$$

For each of the ionic liquids, three terms including ΔG_solvation_ _of_ _cation_, ΔG_solvation_ _of_ _anion_ and ΔG_solvation_ _of_ _ILs_ _as_ _ion_ _pairs_ were calculated by combinatorial use of density functional theory (DFT) alongside a solvation model based on density (SMD) at B3LYP/6-311++G(d,p) level (please refer to Table 4)^59^. We calculated ${\mathrm{\Delta G}}_{\mathrm{solvation}}^{\mathrm{binding}}$ in QM studies by employing Eq. (4). As seen in Table 4, the ${\mathrm{\Delta G}}_{\mathrm{solvation}}^{\mathrm{binding}}$ values obtained from QM studies are larger than those obtained from MD simulation. These differences can be explained by the fact that the distances between cations and anions in the ionic liquid structures are fluctuating during MD simulation affecting the interactions between cations and anions as well as their interactions with their surroundings. Whereas, in QM studies, the distances between cations and anions in the ionic liquid structures are considered to be constant. The QM and MD ${\mathrm{\Delta G}}_{\mathrm{solvation}}^{\mathrm{binding}}$ values for the IL (GTA)(NAL) are comparable (Table 4, entry 4), indicating that this IL has less fluctuation and dynamics than other ILs during 20 ns of MD simulations.

**Table 4:** ΔG_solvation_ _of_ _cation_, ΔG_solvation_ _of_ _anion_ and ΔG_solvation_ _of_ _ILs_ _as_ _ion_ _pairs_ (kcal/mol) of ILs calculated at B3LYP/6-311++G(d,p) level and comparison between ${\mathrm{\Delta G}}_{\mathrm{solvation}}^{\mathrm{binding}}$ (kcal/mol) from QM studies and ${\mathrm{\Delta G}}_{\mathrm{solvation}}^{\mathrm{binding}}$ (kcal/mol) from MD studies.

Entry	Cation	Anion	Codes of structures (ILs)	ΔG_solvation_ _of_ _cation_ (kcal mol^−1^) (QM)	ΔG_solvation_ _of_ _anion_ (kcal mol^−1^) (QM)	ΔG_solvation_ _of_ _ILs_ _as_ _ion_ _pairs_ (kcal mol^−1^) (QM)	${\mathrm{\Delta G}}_{\mathrm{solvation}}^{\mathrm{binding}}$(kcal mol^−1^) (QM)	The average values of ${\mathrm{\Delta G}}_{\mathrm{solvation}}^{\mathrm{binding}}$ (kcal mol^−1^) (MD)
1	BMIM	FBP	(BMIM)(FBP)	−50.3	−60.8	−19.5	91.6	6.14
2	GTA	FBP	(GTA)(FBP)	−64.5	−60.8	−39.1	86.2	57.55
3	BMIM	NAL	(BMIM)(NAL)	−50.3	−75.4	−27.3	98.4	7.91
4	GTA	NAL	(GTA)(NAL)	−64.5	−75.4	−40.6	99.3	96.36
5	BMIM	SAL	(BMIM)(SAL)	−50.3	−57.0	−18.7	88.6	71.80
6	GTA	SAL	(GTA)(SAL)	−64.5	−57.0	−39.3	82.2	58.57
7	BMIM	ACS	(BMIM)(ACS)	−50.3	−59.2	−24.5	85.0	27.88
8	GTA	ACS	(GTA)(ACS)	−64.5	−59.2	−40.5	83.2	2.48
9	BMIM	IBU	(BMIM)(IBU)	−50.3	−62.7	−17.8	95.2	17.81
10	GTA	IBU	(GTA)(IBU)	−64.5	−62.7	−33.6	93.6	60.64

### Comparison between the interactions of GTA-based ILs and BMIM-based ILs with the cell membrane via docking and MD simulations

**Figure 10:**
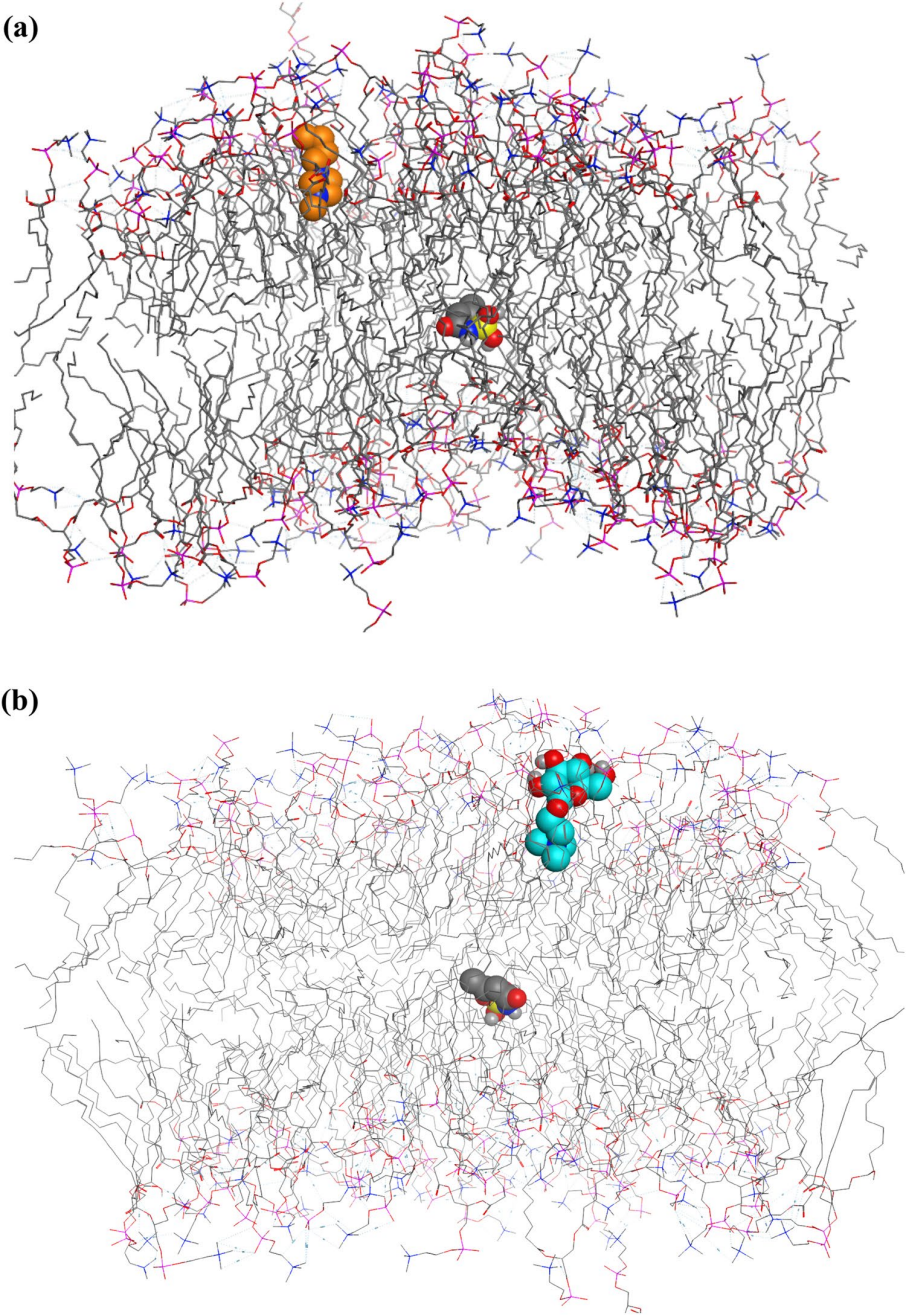
1000 docking runs were performed for each docking of the ILs on the membrane structure. (**a**) The selected initial structure of (BMIM)(ACS) in the MD simulation was the most populated conformation in each cluster with the best pose and most negative binding energy (the molecule shown in orange color is BMIM and the molecule shown in gray color is ACS); both anion and cation are shown in VDW format and membrane is shown in line style. (**b**) (GTA)(ACS) interacts with membrane and its most populated conformation was chosen as the input for MD simulation (the molecule shown in blue color is GTA and the molecule shown in gray color is ACS). Both anion and cation are shown in VDW format and membrane is shown in line style.

In this part of study, we ran two molecular dynamics simulations for the complexes of a membrane with two examples of ionic liquids, including (BMIM)(ACS) and (GTA)(ACS). First, we obtained a PDB structure of one of the membrane models, including 128 molecules of POPC (phosphatidylcholine)^60–62^. Before initiation of MD simulation, we performed the grid box searching process using Auto Dock 4.2.2 software^63^. Before finding the grid box, water molecules in the primary structure of POPC were removed using Discovery studio 4.5.^64^ to find the best positions of (BMIM)(ACS) and (GTA)(ACS) in the structure of the membrane. Then, we conducted two separate docking procedures for both ILs in the presence of the membrane. The spacing between grid points for both docking processes was considered to be 0.375 Å, and the grid box size was set at $126\times 126\times 126$ Å^3^. The (BMIM)(ACS) and (GTA)(ACS) as ligands were deemed to be flexible, and the grid searching was carried out at local search genetic algorithm (LGA)^65–67^. 1000 docking runs were performed for each docking of ionic liquids in the membrane structure. Then the most populated conformation in each cluster and the best pose with the most negative binding energy for each IL including (BMIM)(ACS) and (GTA)(ACS) (Fig. 10a,b) were chosen for MD simulations.

Figures 10a,b indicate that both ionic liquids are dissociated in the membrane. In this part of the study for MD simulations, we employed force field parameters for the lipids, provided by Berger and et al.^68^. Also, some of the parameters for these simulations were obtained from the Tieleman website. The topology files for (BMIM)(ACS) and (GTA)(ACS) were constructed based on the Gromos 53a6 force field using the PRODRG 2. X online server. Two complexes, including (1) membrane and (BMIM)(ACS) and (2) membrane and (GTA)(ACS) were solvated by a layer of SPC water in all directions^69^. The simulation box in both cases was considered a triclinic box with faces at least 1.0 nm apart from the closest atom from each system. For the first system, including Membrane and (BMIM)(ACS), 50,371 water molecules were considered as solvent, and to neutralize this system, 2 Cl ions were added. For other systems, including Membrane and (GTA)(ACS), 50,407 water molecules were considered solvent, and one Cl ion was added to neutralize this system. We performed the minimization process for both mentioned systems in three steps. The steepest descent and then conjugate gradient methods were used for the primary two steps. The steepest descent was used in the third step. The emtol value was 100.0 kJ mol^−1^. nm. in both systems. After equilibration of both systems in NVT and NPT ensembles and reaching to 300 K and 1 bar^52,53^, the MD production steps for two systems were initiated at 300 K with a time step of 1 fs and both MD procedures were performed in 7 ns. It should be mentioned that the Parrinello–Rahman barostat^54^ and Nose–Hoover thermostat algorithms^55^ were considered in the MD simulation. For both ILs, the binding free energies on the membrane were calculated by consideration of molecular Mechanics/Poisson–Boltzmann Surface Area (MM/PBSA) method by taking 70 snapshots during 7 ns of MD simulation^56^. The calculated energy terms can be seen in Table 5, where both ionic liquids, (BMIM)(ACS) and (GTA)(ACS), have negative binding energy values towards the membrane (−1645.878 kJ mol^−1^ and −1117.214 kJ mol^−1^, respectively). The results in Table 5 indicate that the interactions of both of the ILs with the membrane are thermodynamically very favorable; but the less binding energy of (GTA)(ACS) toward the membrane reveals that (GTA)(ACS) can be released from the membrane to the cytosol much easier than (BMIM)(ACS). This result suggests that the (GTA)(ACS) IL can be more effective in drug delivery than the (BMIM)(ACS) IL.

**Table 5:** Various energy terms obtained through MM/PBSA calculations.

MM/PBSA	The membrane and (BMIM)(ACS)	The membrane and (GTA)(ACS)
van der Waals energy (kJ/mol)	−97.806 ± 21.917	−206.959 ± 21.012
Electrostatic energy(kJ/mol)	−1292.754 ± 456.727	−565.538 ± 162.632
Polar solvation energy(kJ/mol)	−245.766 ± 410.125	−328.446 ± 278.702
Non-polar solvation energy (kJ/mol)	−9.551 ± 2.010	−16.271 ± 2.401
Binding energy (kJ/mol)	−1645.878 ± 134.122	−1117.214 ± 137.111

Also, one snapshot of each of the systems through MD simulation was taken to show the conditions of these systems during the molecular dynamics simulations. As seen in Figs. S19 and S20, chloride ions are shown in yellow color, the membrane structure is shown by line model, and point model in red displays solvent (water molecules). Both BMIM and ACS are shown by the ball and stick model in Fig. S19 and BMIM is displayed in pink colour. In Fig. S20, GTA ion is shown by the ball and stick model in green colour.

We also obtained the equilibrium constants for the formation of ionic liquids listed in Table 3 using the Eq. 6 (Please refer to Table S5).

6 $$ \Delta {\text{G}}_{{{\text{binding}}}} = \, - {\text{RTLn K}}_{{\text{formation of ionic liquids}}} $$

The formation constants in Table S5 indicate that the ionic liquids, particularly those based on GTA, remain undissociated until they achieve their target cells. After being delivered to their target cells, the transportation of drugs in the structures of GTA-based ILs can happen in various ways. As GTA consists of fragments such as glucose and choline, we assume that the cation (GTA) finds its way through the cells with similar mechanisms as glucose and choline do. Cell types can differ in how glucose is transported through their membranes, but some cells employ glucose transporters to do their tasks^70,71^. Moreover, choline is expected to travel through the cells utilizing sodium-coupled choline transporter 1 (CHT1 or SLC5A7), sodium-independent choline transporter-like (CTL) proteins, as well as organic cations^72^. As the introduction of GTA into the cells is complete, we expect the drugs (the anions in the designed ILs) will travel into the cells to conserve the neutrality of the target cells.

### The validation of the QM methodology employed in this study

**Figure 11:**
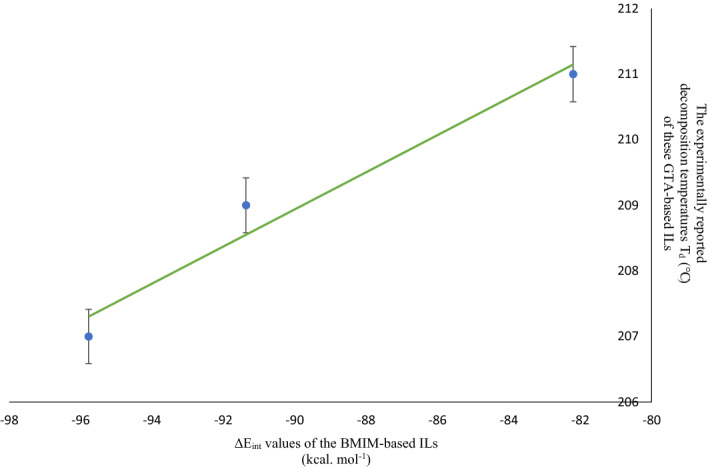
The experimentally reported decomposition temperatures of ionic liquids including (GTA)(Trp), (GTA)(His) and (GTA)(Tyr) (℃) against their calculated ΔE_int_ values (kcal. mol^-1^) obtained at the B3LYP/6-311++G(d,p) level, (error bar % = 0.2).

As explained above, the two groups of ionic liquids BMIM-based and GTA-based ILs were studied at B3LYP/6-311++G(d,p) level. In our previous paper^51^, we demonstrated that this level of theory is an efficient method to obtain the binding energies of the designed ILs and predicting properties such as melting point. To validate the calculation level for BMIM-based ILs, the well-known ionic liquids such as (BMIM) Cl, (BMIM) Br, (BMIM) (TF), (BMIM) (PF_6_), (BMIM) (TFSI), and (BMIM) (BF_4_) were chosen, and their ΔE_int_ and ∆E_CEC_ values were calculated at B3LYP/6-311++G(d,p) level. Then we achieved the linear line with y = 6.047x − 446.08, R^2^ = 0.9937 (Fig. S21) by plotting the experimentally reported melting points of these known ILs^73,74^ versus their ∆E_CEC_ values. Table S6 includes the melting points of the mentioned ILs. To validate the calculation level for GTA-based ILs, we chose three known GTA-based ILs containing the conjugate base of an amino acid as the anion of the IL, including (GTA)(Trp), (GTA)(His), and (GTA)(Tyr)^29^. As the decomposition temperatures of these ionic liquids have been reported, we first calculated the ΔE_int_ and ∆E_CEC_ of these ionic liquids at the B3LYP/6-311++G(d,p) level. Then the decomposition temperatures (℃) of these GTA-based ILs were plotted against their ΔE_int_ values (kcal mol^−1^). The resulted linear line is $y=0.283x+234.42,$ R^2^ = 0.9608, where y indicates the decomposition temperatures (℃) and *x* is ΔE_int_ values (Fig. 11).

The reason we chose (GTA)(Trp), (GTA)(His) and (GTA)(Tyr) was that the anions of all the designed pharmaceutically active GTA-based ILs have the aromatic ring or at least a heterocycle ring. The optimized structures of ionic liquids including (GTA)(Trp), (GTA)(His) and (GTA)(Tyr) at the B3LYP/6-311++G(d,p) level can be seen in Fig. S22, and the ΔE_int_ and ∆E_CEC_ of these ionic liquids are listed in Table S7.

#### Toxicity evaluation of BMIM-based ILs and GTA-based ILs using theoretical studies

One of the critical steps in designing new structures with the potential function as drugs is the evaluation of their toxicity. As these structures can influence human health, many strategies have been developed to measure their toxicities^75^; and there might be a possibility of drug failures at the final stage of the drug discovery process^76^. Computational methods have been devised to help researchers prioritize the choice of their newly designed structures, reducing the time needed in the drug discovery procedure. In this research, we decided to computationally measure and compare the toxicity of BMIM-based ILs and GTA-based ILs. We used the Toxicity Estimation Software Tool (TEST) to achieve our goal^77^. As the anions in this study were known drugs and identical for both groups of ionic liquids, we calculated the toxicity of cations including (BMIM) and (GTA). The method used to predict the toxicities of the aforementioned compounds was hierarchical clustering. This methodology is based on a variation of Ward’s Method^78^. To estimate the toxicity using TEST, the Oral rat LD_50_ was calculated for (BMIM) and (GTA). The Oral rat LD_50_ manifests the amount of the chemical compound entered the rat's body through oral injection and can be lethal to half of the population of the rats under study. The Oral rat LD_50_ can be explained by the mass of the chemical substance in milligrams per kilogramme of the rat's body weight^79^. For each structure, the similar compounds with the experimentally measured values of the Oral rat LD_50_ were chosen and their Oral rat LD_50_ values were calculated by TEST. Figures S23 and S24 demonstrate the validity of the TEST method by comparing the experimental Log Toxicity (mol/kg) values with the calculated Log Toxicity (mol/kg) values (obtained by TEST method) for the known structures.

The Oral rat LD_50_ for (BMIM) was determined to be 1499.22 mg/kg according to Tables S8 and S9 and Figure S23, where $y=0.85x+0.28, {R}^{2}=0.8823$. Considering the Tables S10, S11 along with Figure S24, where $y=0.79x+0.67, {R}^{2}=0.8668$, the oral rat LD_50_ value for (GTA) was found to be 4955.34 mg/kg. These results suggest that (GTA) might have fewer adverse effects than (BMIM).

## Conclusion

In this study, we investigated two groups of ILs-based drug delivery systems. The first group of ionic liquids was based on BMIM (see Table 1 for nomenclatures), including (BMIM) (IBU), (BMIM) (ACS), (BMIM) (NAL) and (BMIM) (SAL); the second group of ionic liquids (designed in this study) was based on GTA (for nomenclatures, refer to Table 2), including (GTA) (IBU), (GTA) (ACS), (GTA) (SAL), (GTA) (NAL), and (GTA) (FBP). The B3LYP/6-311++G(d,p) level of theory was first used to calculate the binding energy between the anion and cation of these ILs. Furthermore, we used MD studies based on the Gromos 43 a1 force field to obtain binding energies of some of the BMIM-based and GTA-based ILs. Twenty nanoseconds of MD simulation were performed for each of the mentioned ILs. Then binding energies of ionic liquids through MD simulation were calculated using the Molecular Mechanics/Poisson–Boltzmann Surface Area (MM/PBSA) method. Then, the MD ΔG_binding_ values were plotted against the QM ΔE_int_ values for the BMIM-based ILs, resulting in a linear graph. The same linear graph was obtained for the GTA-based ILs by plotting their MD ΔE_int_ values, and their QM ΔE_int_ values. These linear graphs illustrated the existence of the link between MD and QM studies. Finally, we chose (BMIM) (ACS) and (GTA) (ACS) to study their interactions with the cell membrane. In this part of the study, Auto Dock 4.2.2 software, GROMACS software, and Gromos 53a6 force field were used for the MD simulations of the mentioned ionic liquids with the membrane. The ILs of (BMIM)(ACS) and (GTA)(ACS) have negative binding energy values towards the membrane (−1645.878 kJ mol^−1^ and −1117.214 kJ mol^−1^, respectively), indicating that the interactions of both of the ILs with the membrane are thermodynamically very favourable. The binding energy of the (GTA)(ACS) IL toward the membrane reveals that this IL can be released from the membrane to the cytosol much easier than the (BMIM)(ACS) IL. The above observations suggest that the (GTA)(ACS) IL might be more effective in drug delivery than the (BMIM)(ACS) IL. The information from this project might open new windows toward the synthesis of new, more effective, and less toxic drug delivery systems compared to the traditional ones.

## Supplementary Information

Supplementary Figures.

Supplementary Figures.

Supplementary Figures.

Supplementary Figures.

Supplementary Information 1.

Supplementary Information 2.

## Data Availability

All data generated or analysed during this study are included in this published article [and its supplementary information files].
