# Supplementary material for: Design of ionic liquids containing glucose and choline as drug carriers, finding the link between QM and MD studies
Source: Sci Rep. 2022 Dec 19;12:21941. doi: 10.1038/s41598-022-25963-z (PMC9763358; doi:10.1038/s41598-022-25963-z)
Supplement: Supplementary file 1 — Supplementary Figures. [file 41598_2022_25963_MOESM1_ESM.pdf]

Design of ionic liquids containing Glucose and Choline as drug carriers, finding the link between QM and MD studies

Sepideh Kalhor, Alireza Fattahi*
Department of Chemistry, Sharif University of Technology, Tehran, Iran
* Corresponding Author: fattahi@sharif.edu

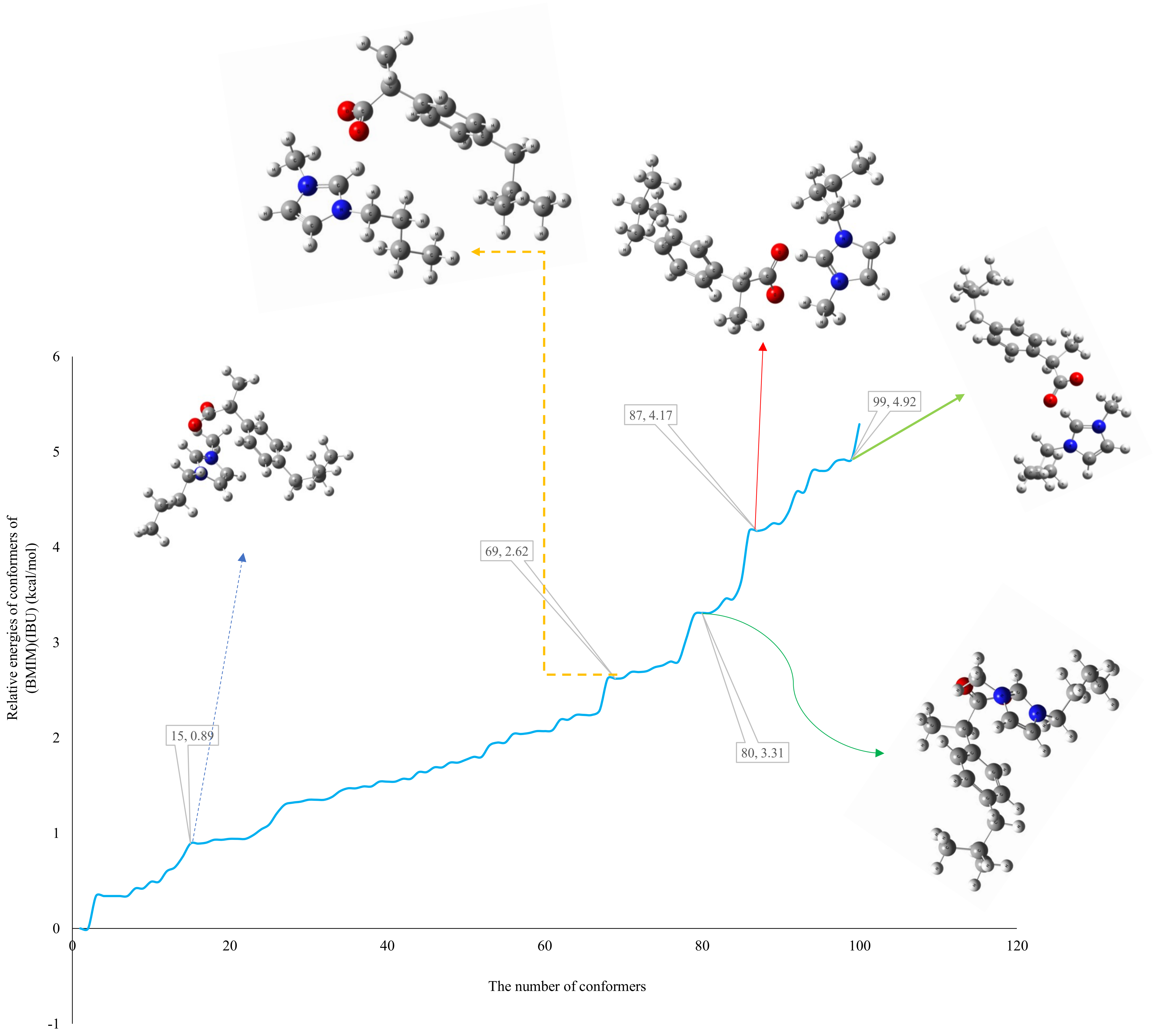

Figure S1. Relative energies of the conformers of (BMIM) (IBU) with respect to the most stable conformer

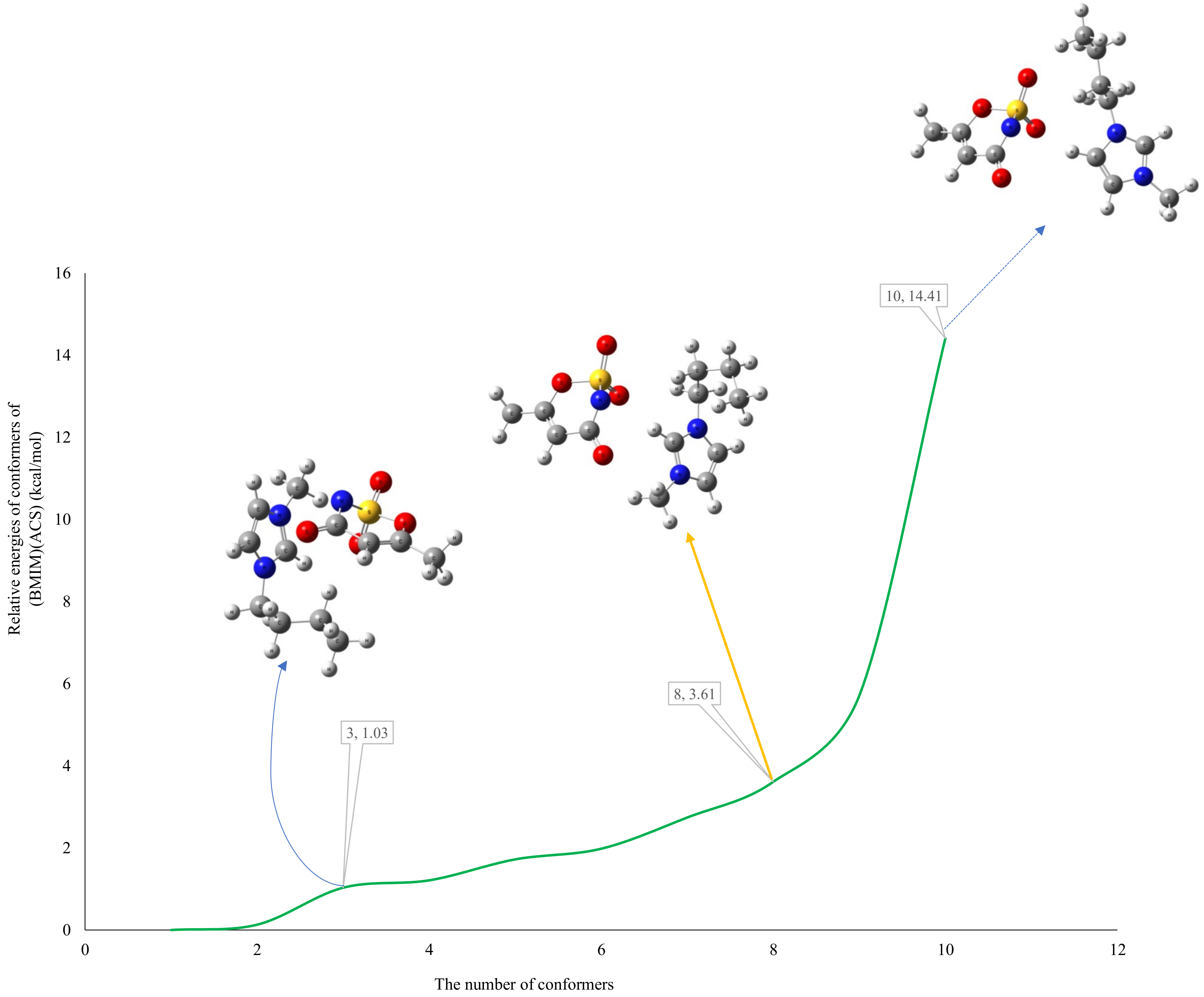

Figure S2. Relative energies of the conformers of (BMIM) (ACS) with respect to the most stable conformer

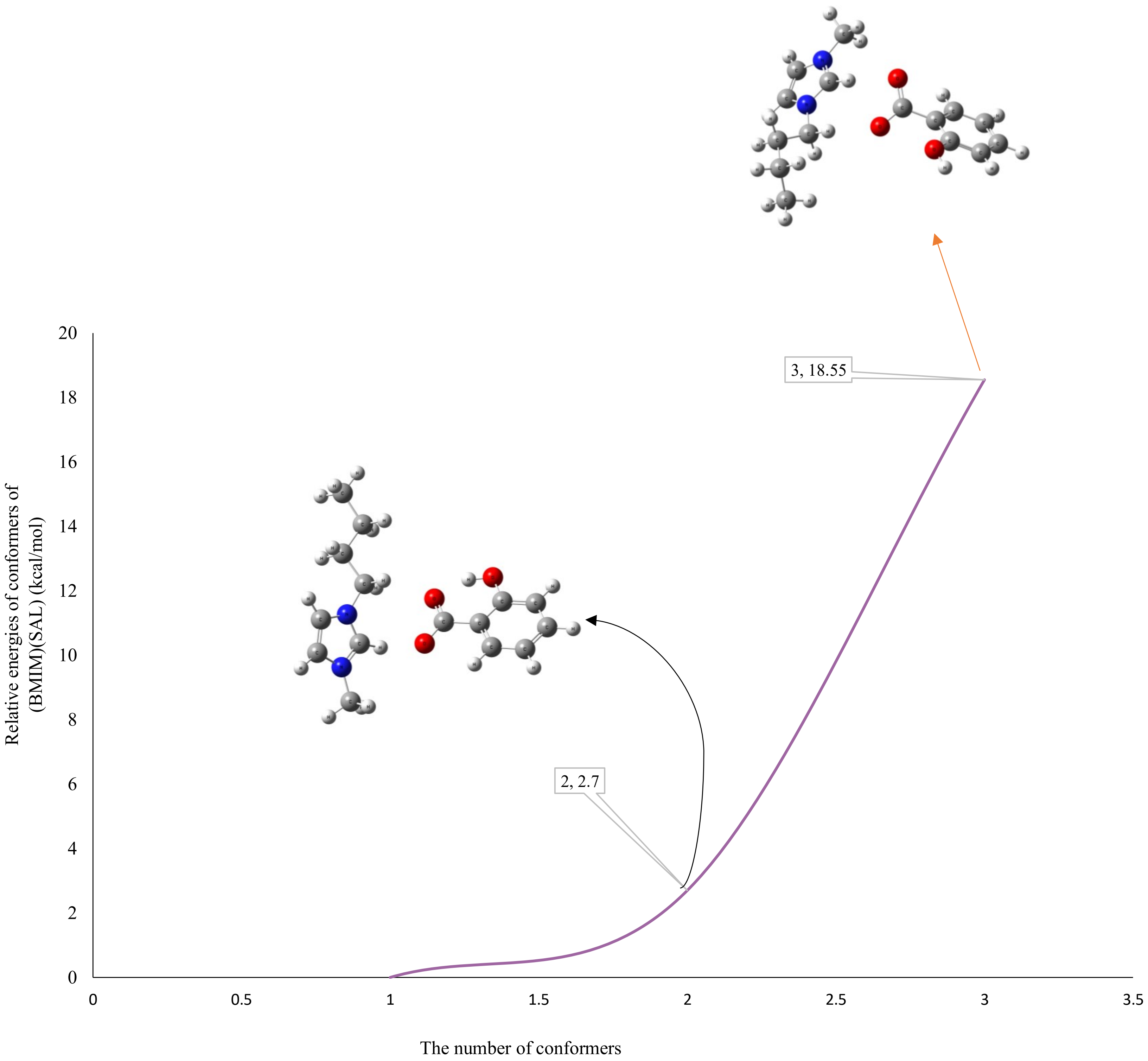

Figure S3. Relative energies of the conformers of (BMIM) (SAL) with respect to the most stable conformer

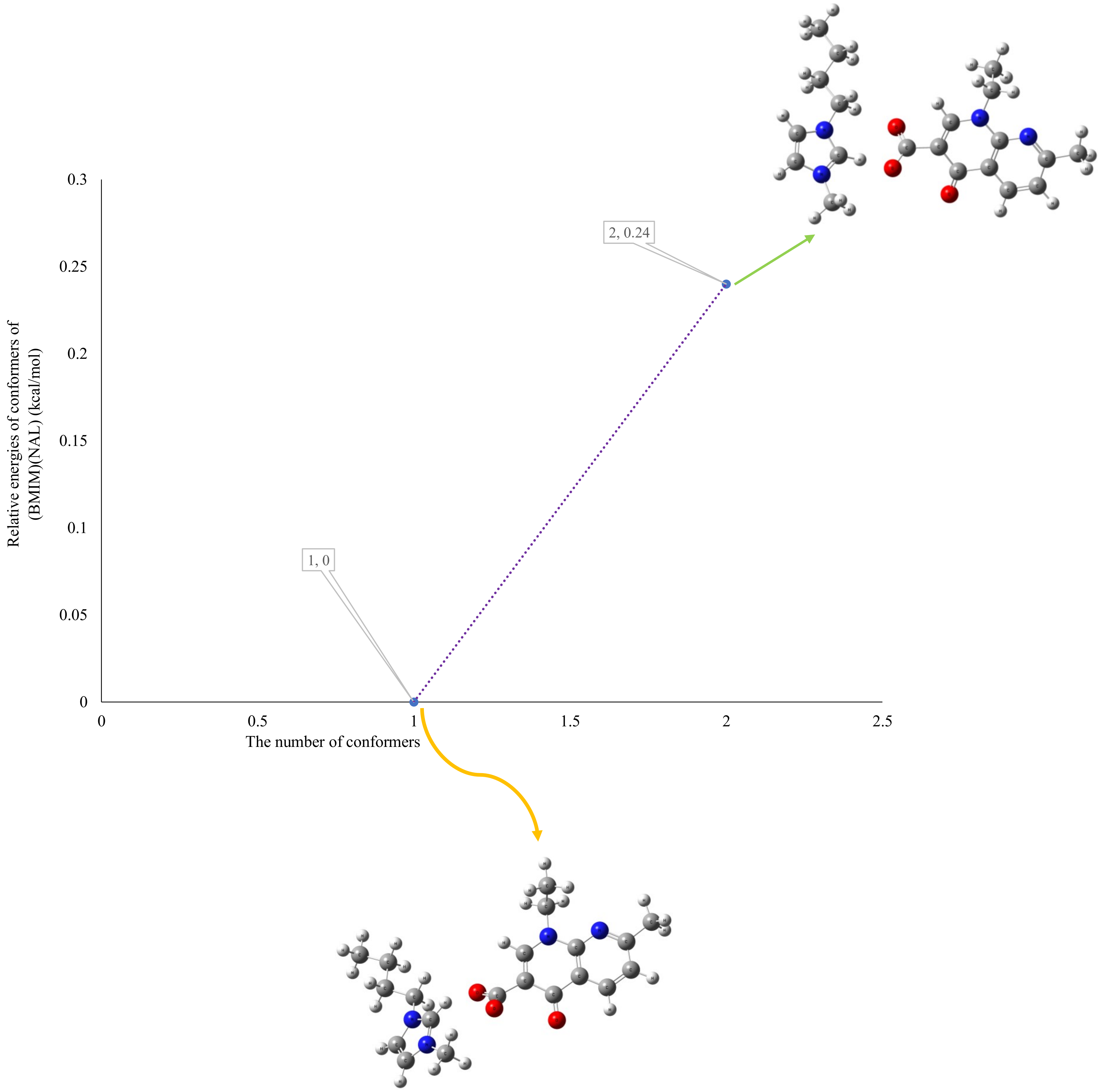

Figure S4. Relative energies of the conformers of (BMIM) (NAL) with respect to the most stable conformer

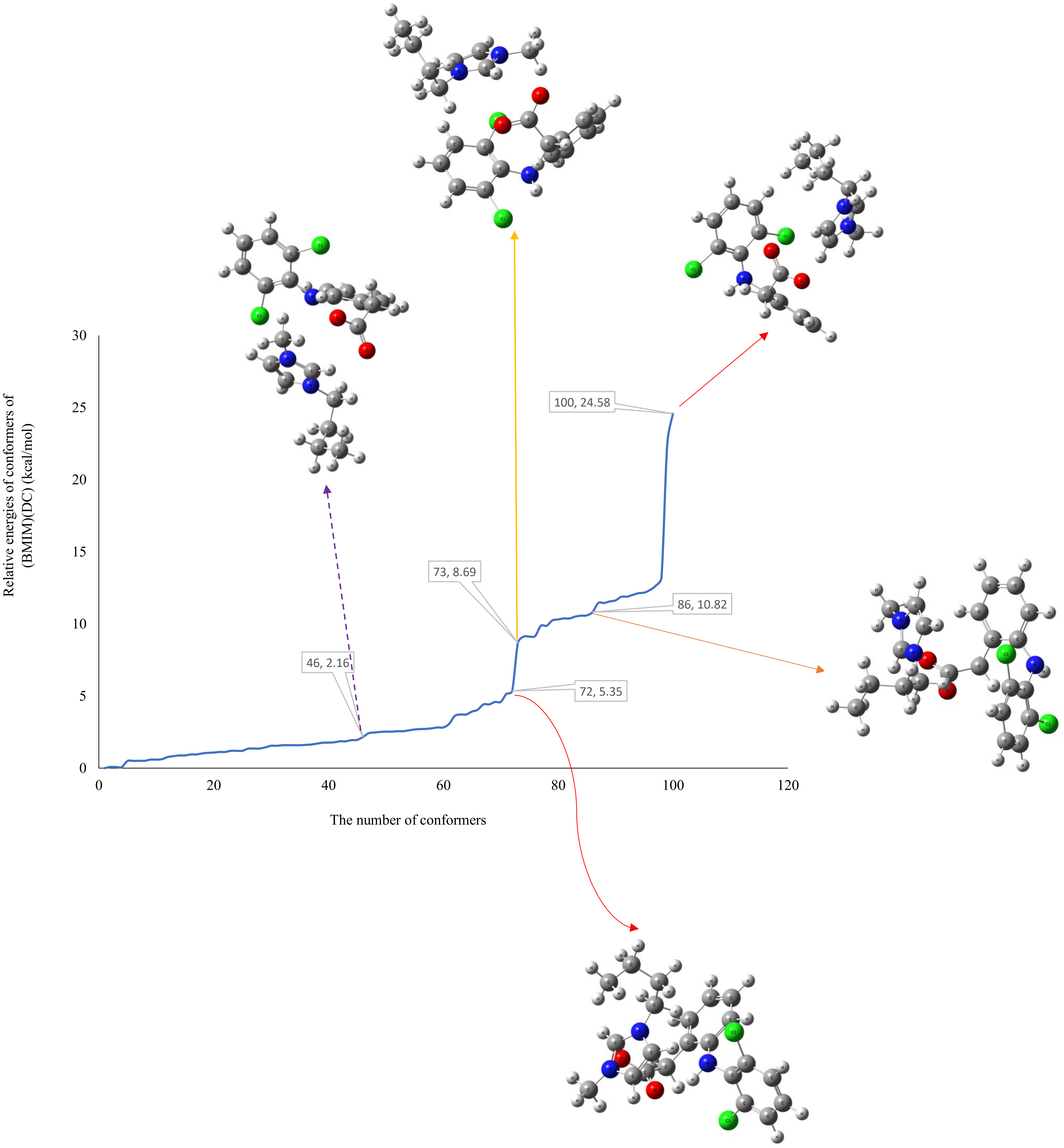

Figure S5. Relative energies of the conformers of (BMIM) (DC) with respect to the most stable conformer

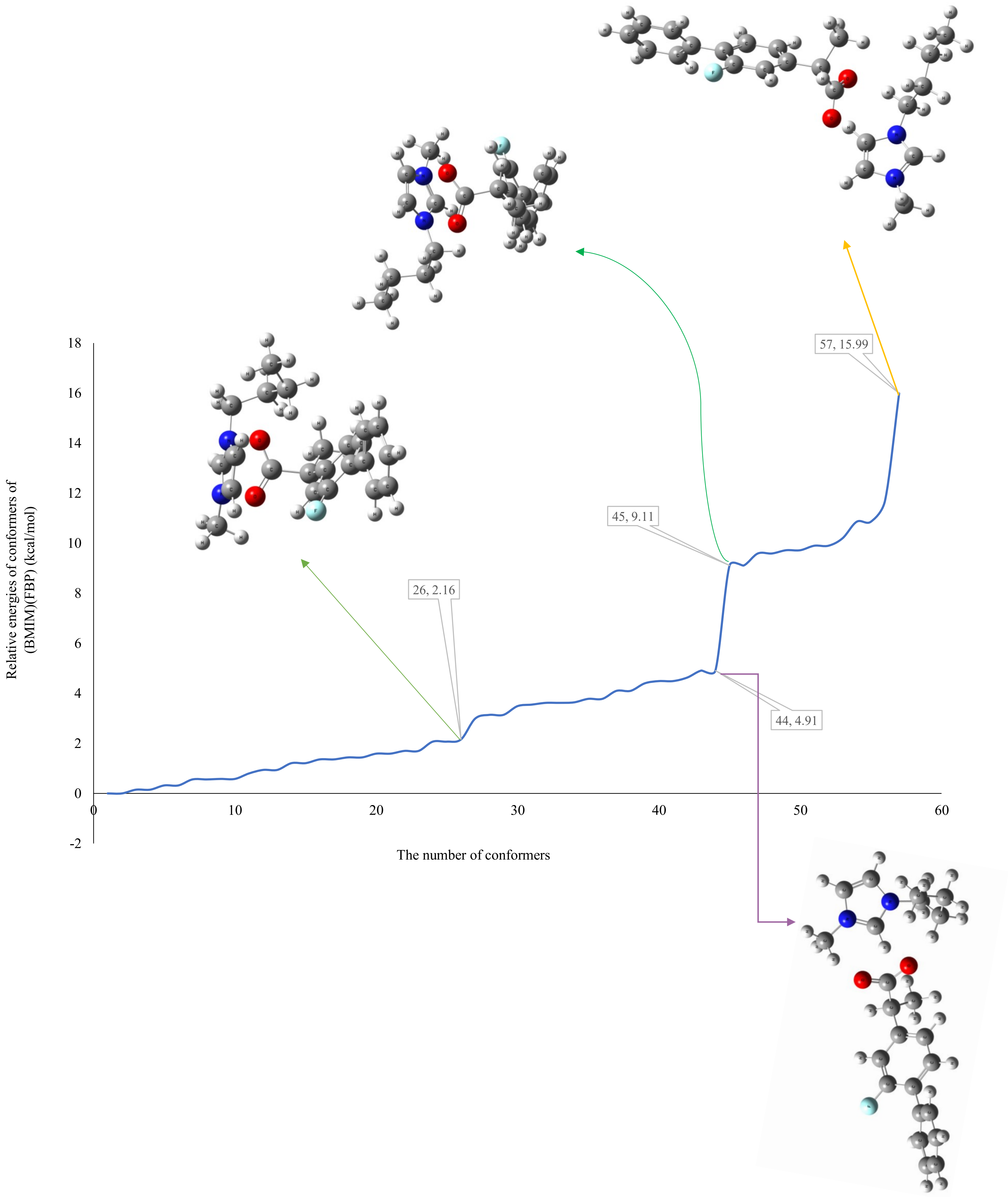

Figure S6. Relative energies of the conformers of (BMIM) (FBP) with respect to the most stable conformer

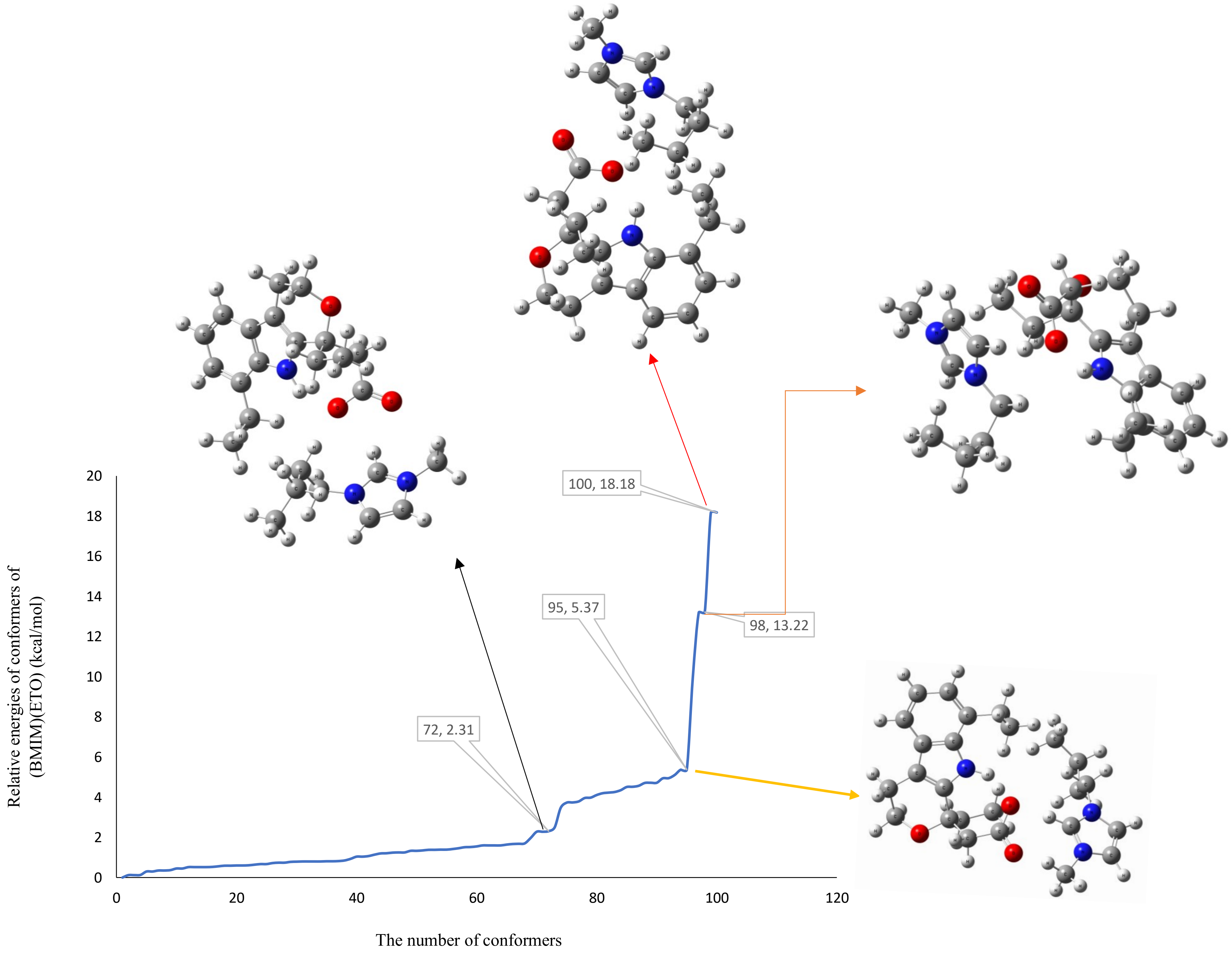

Figure S7. Relative energies of the conformers of (BMIM) (ETO) with respect to the most stable conformer

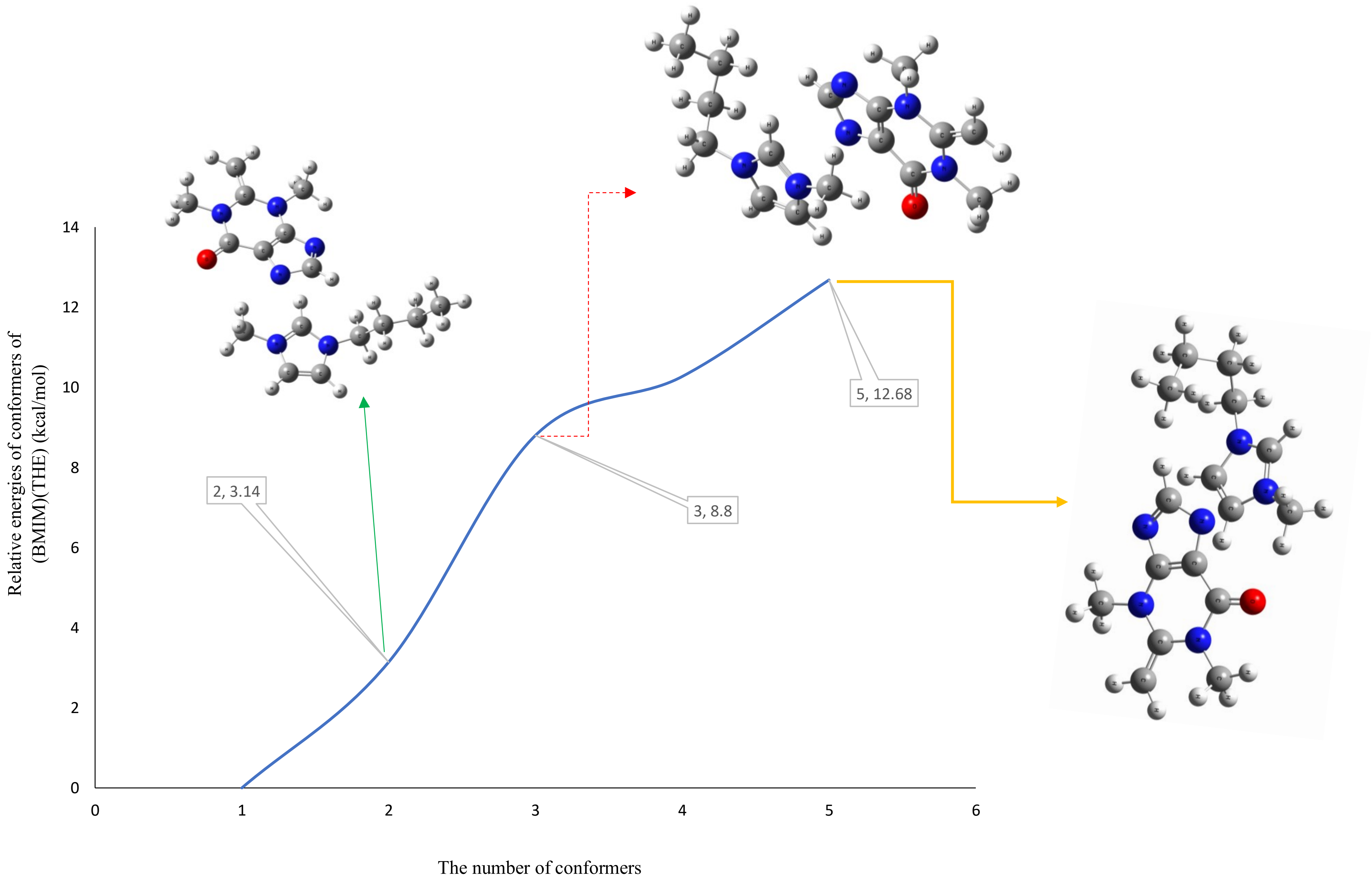

Figure S8. Relative energies of the conformers of (BMIM) (THE) with respect to the most stable conformer
