# Supplementary material for: Design of ionic liquids containing glucose and choline as drug carriers, finding the link between QM and MD studies
Source: Sci Rep. 2022 Dec 19;12:21941. doi: 10.1038/s41598-022-25963-z (PMC9763358; doi:10.1038/s41598-022-25963-z)
Supplement: Supplementary file 2 — Supplementary Figures. [file 41598_2022_25963_MOESM2_ESM.pdf]

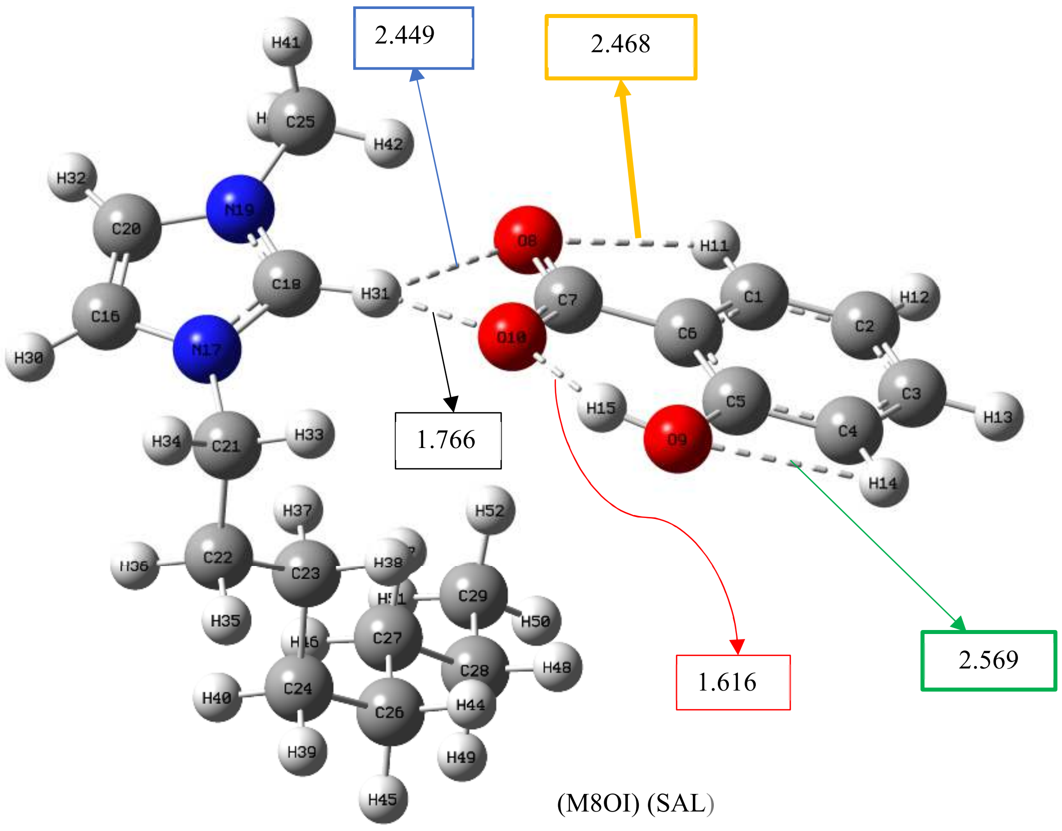

Figure 9. Optimized geomety of the lowest-energy conformer of 3-methyl-1-octyl-1H-imidazol-3-ium- 2-hydroxybenzoate (M8OI) (SAL) calculated at the B3LYP/6-311++G(d,p) level.

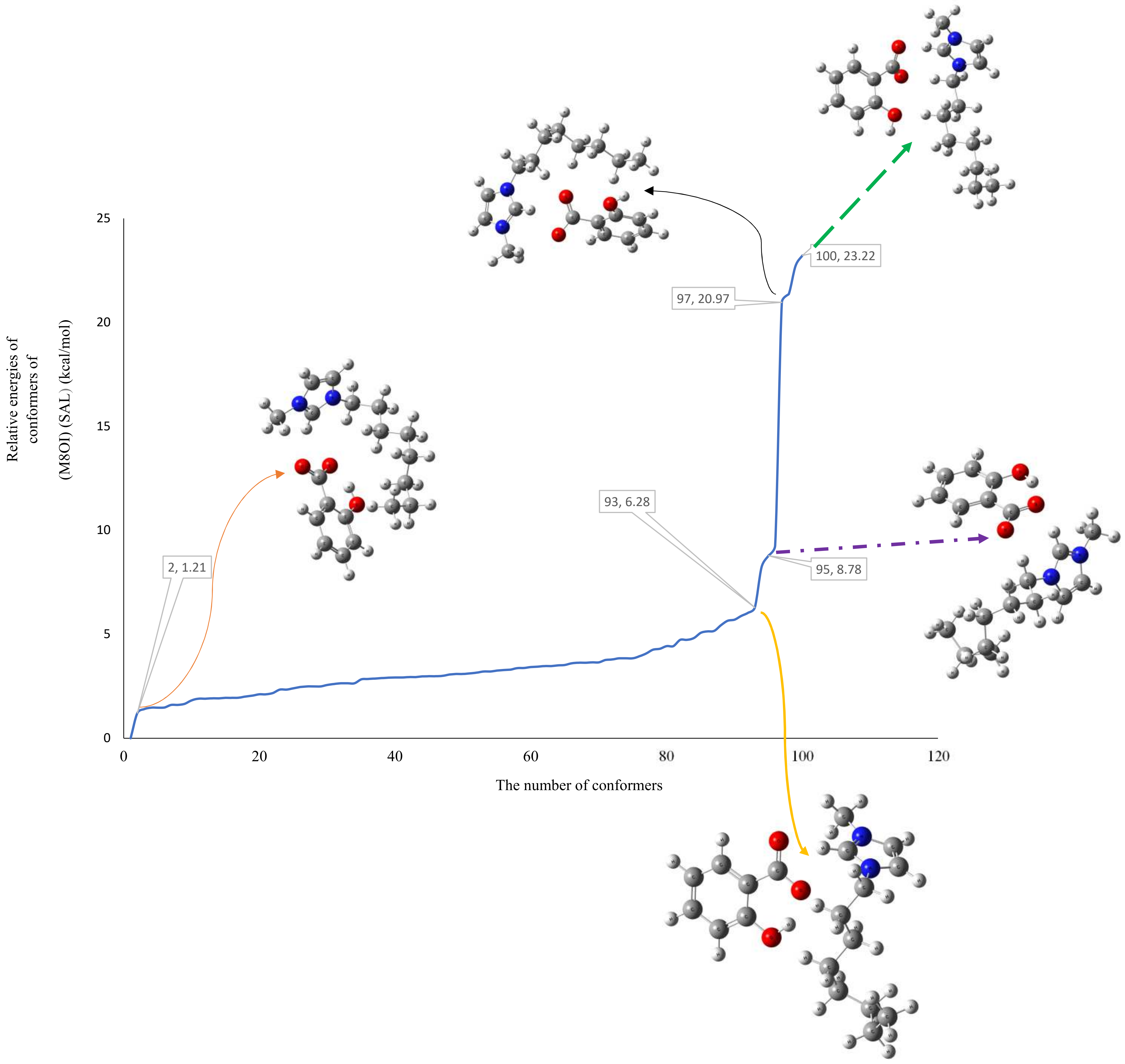

Figure S10. Relative energies of the conformers of 3-methyl-1-octyl-1H-imidazol-3-ium- 2-hydroxybenzoate (M8OI) (SAL) with respect to the most stable conformer.
