# Supplementary material for: Design of ionic liquids containing glucose and choline as drug carriers, finding the link between QM and MD studies
Source: Sci Rep. 2022 Dec 19;12:21941. doi: 10.1038/s41598-022-25963-z (PMC9763358; doi:10.1038/s41598-022-25963-z)
Supplement: Supplementary file 3 — Supplementary Figures. [file 41598_2022_25963_MOESM3_ESM.pdf]

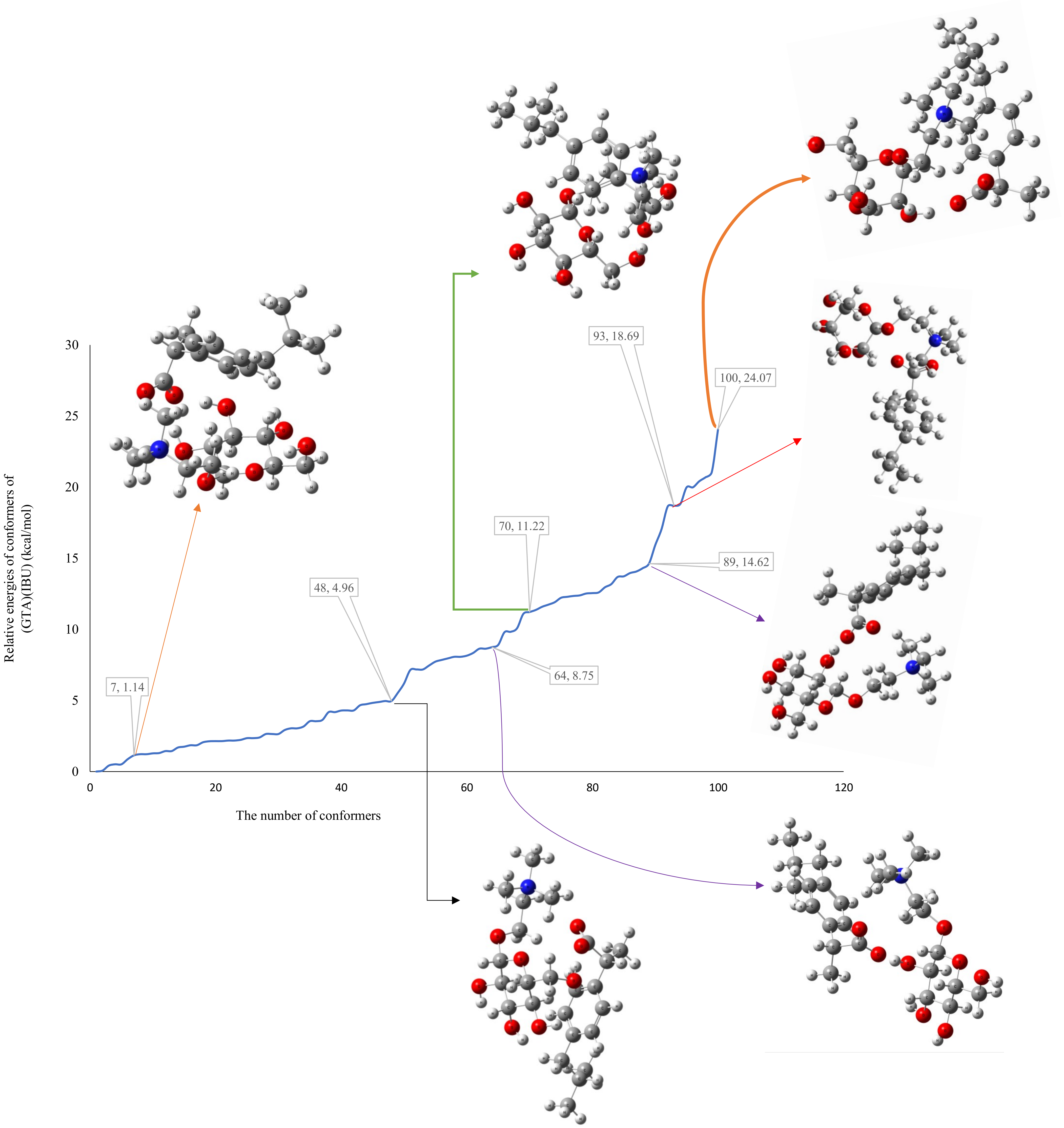

Figure S11. Relative energies of the conformers of (GTA) (IBU) with respect to the most stable conformer

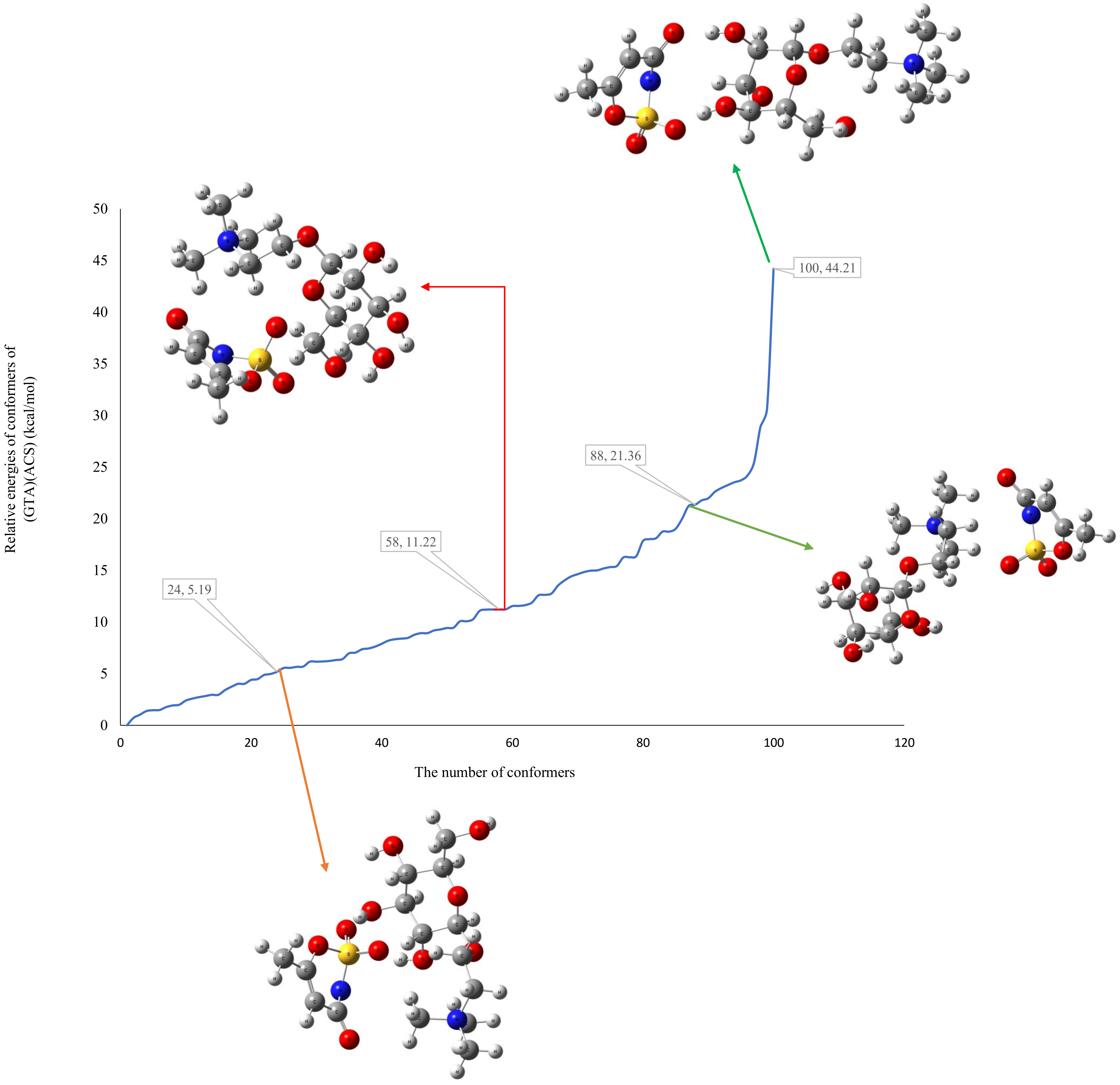

Figure S12. Relative energies of the conformers of (GTA) (ACS) with respect to the most stable conformer

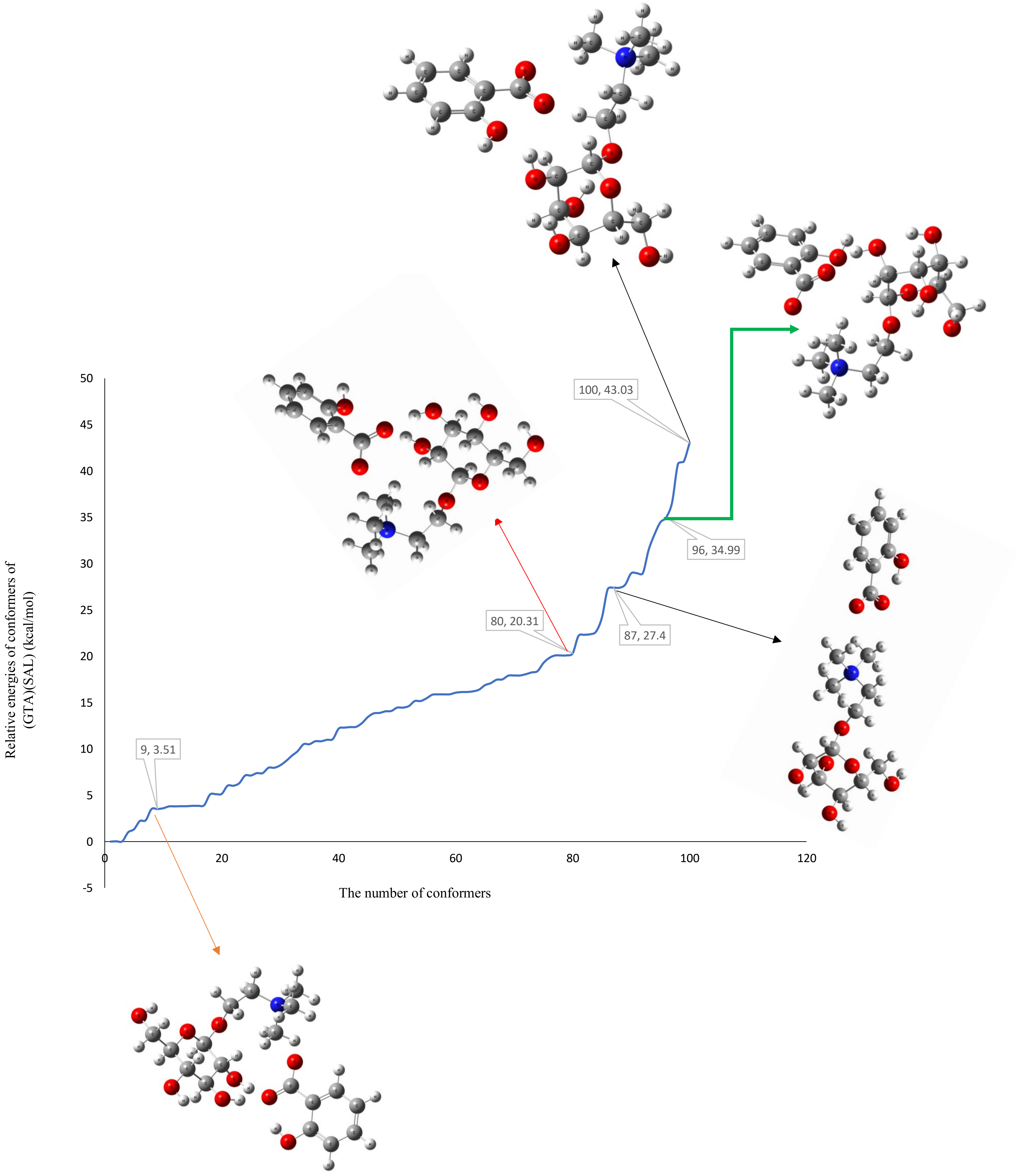

Figure S13. Relative energies of the conformers of (GTA) (SAL) with respect to the most stable conformer

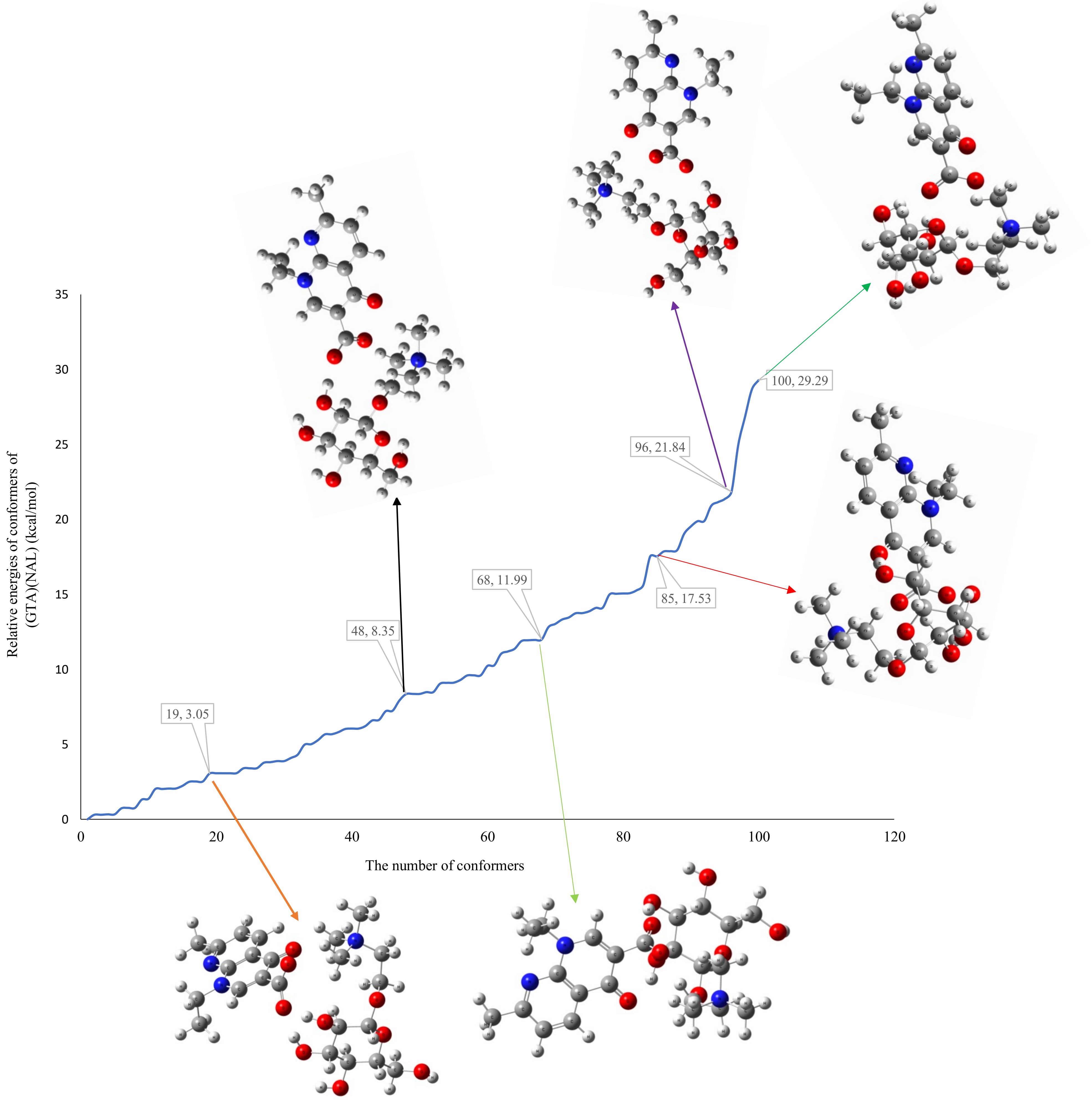

Figure S14. Relative energies of the conformers of (GTA) (NAL) with respect to the most stable conformer

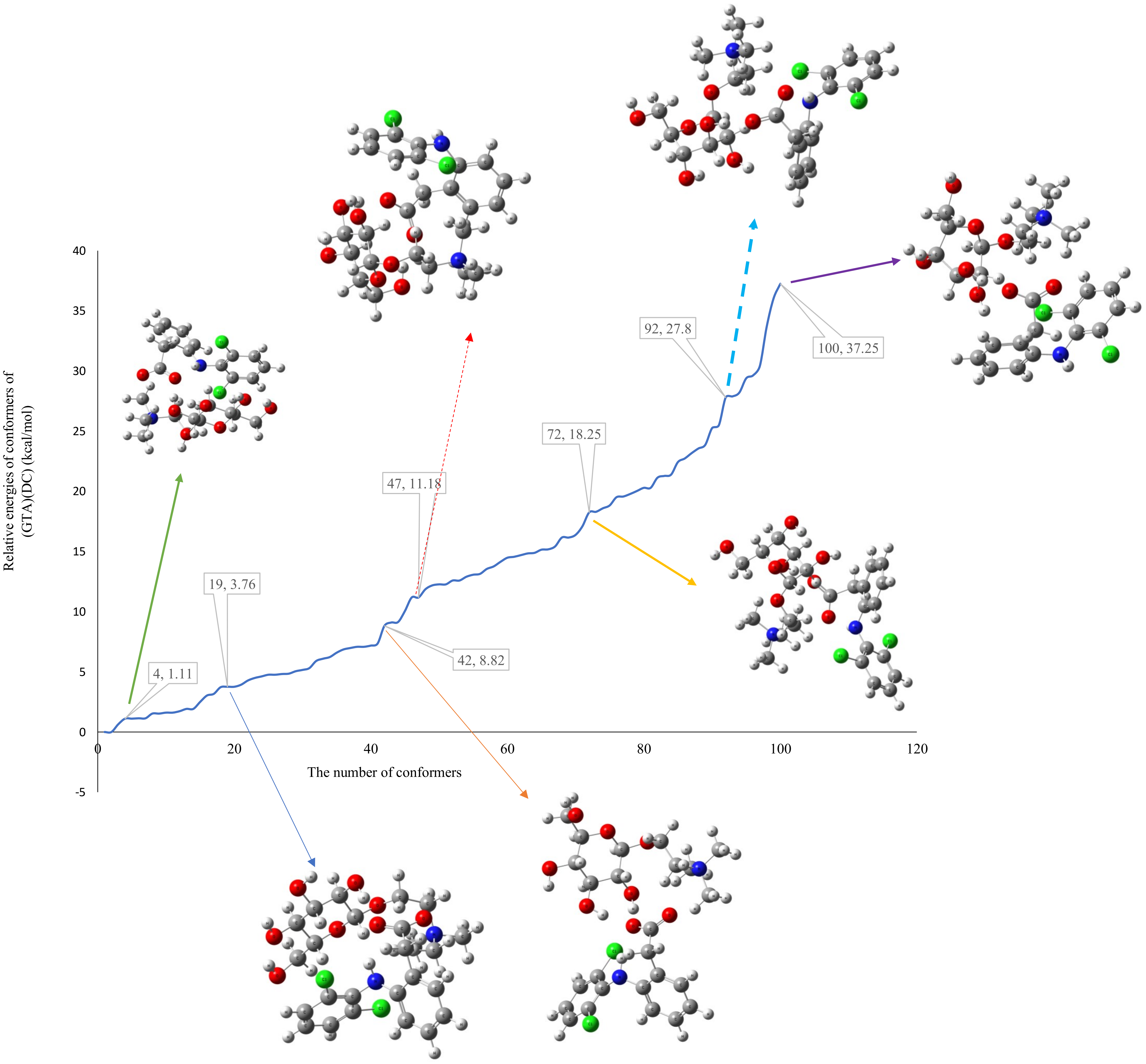

Figure S15. Relative energies of the conformers of (GTA) (DC) with respect to the most stable conformer

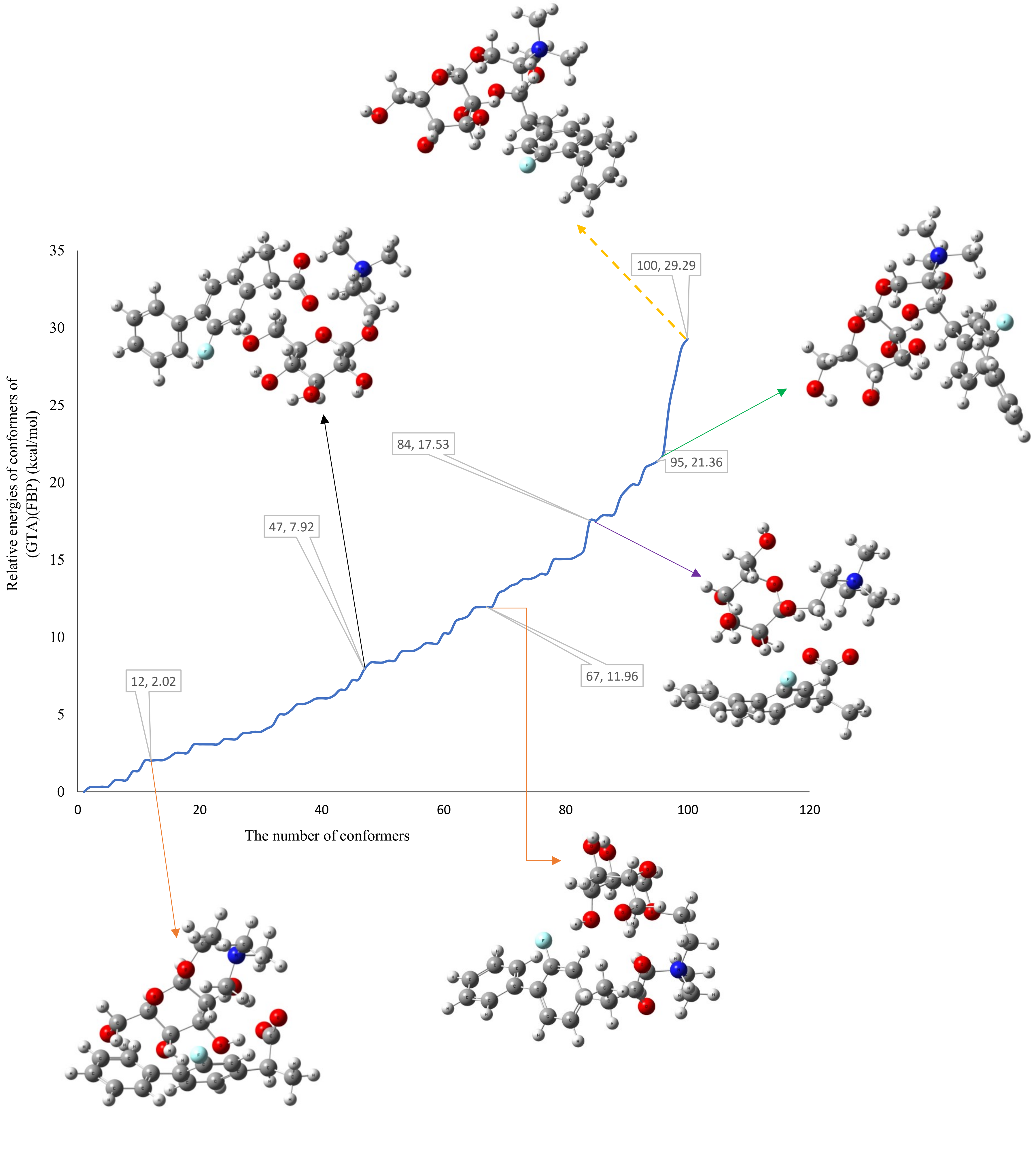

Figure S16. Relative energies of the conformers of (GTA) (FBP) with respect to the most stable conformer
