# Supplementary material for: Design of ionic liquids containing glucose and choline as drug carriers, finding the link between QM and MD studies
Source: Sci Rep. 2022 Dec 19;12:21941. doi: 10.1038/s41598-022-25963-z (PMC9763358; doi:10.1038/s41598-022-25963-z)
Supplement: Supplementary file 4 — Supplementary Figures. [file 41598_2022_25963_MOESM4_ESM.pdf]

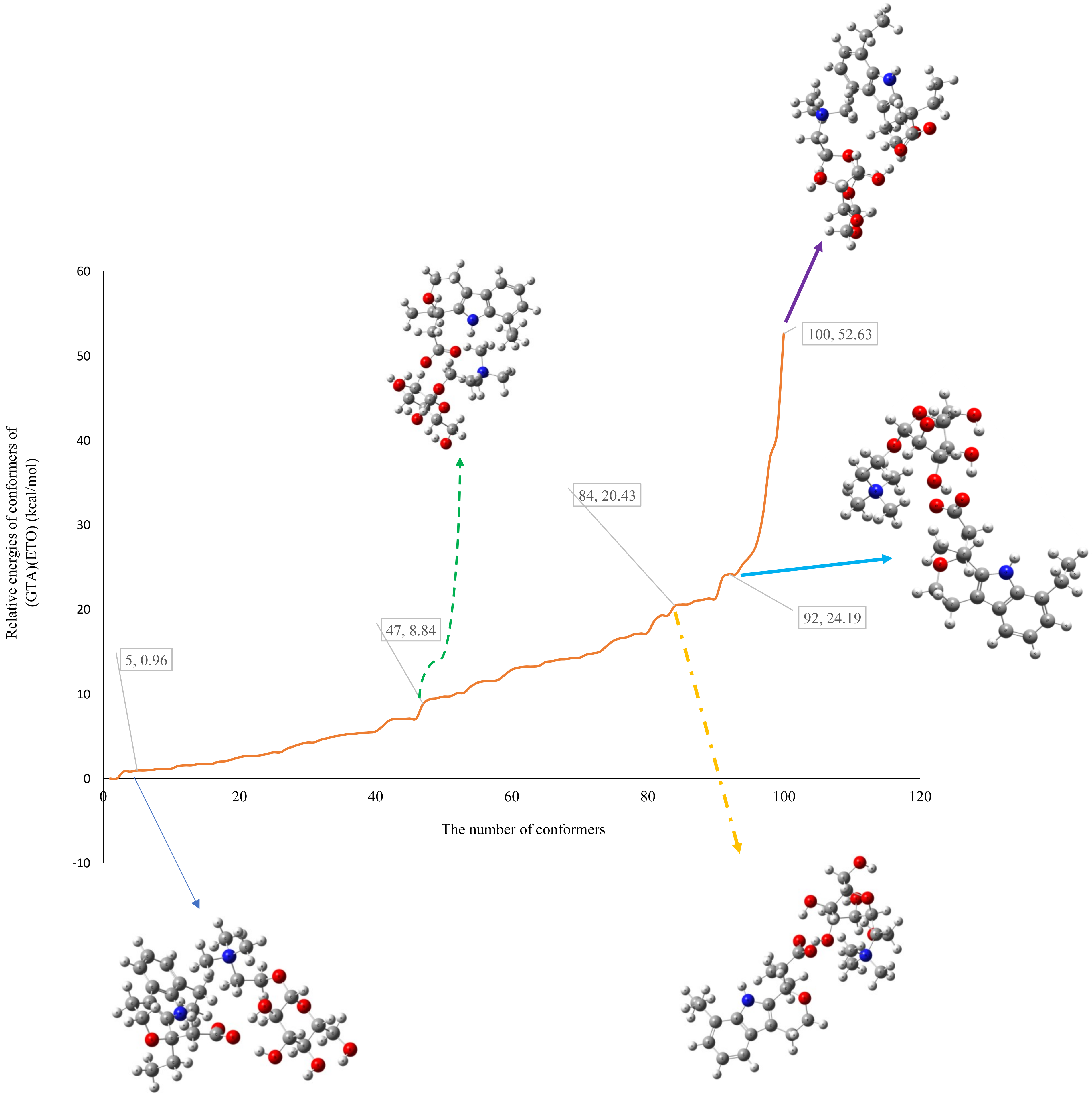

Figure S17. Relative energies of the conformers of (GTA) (ETO) with respect to the most stable conformer

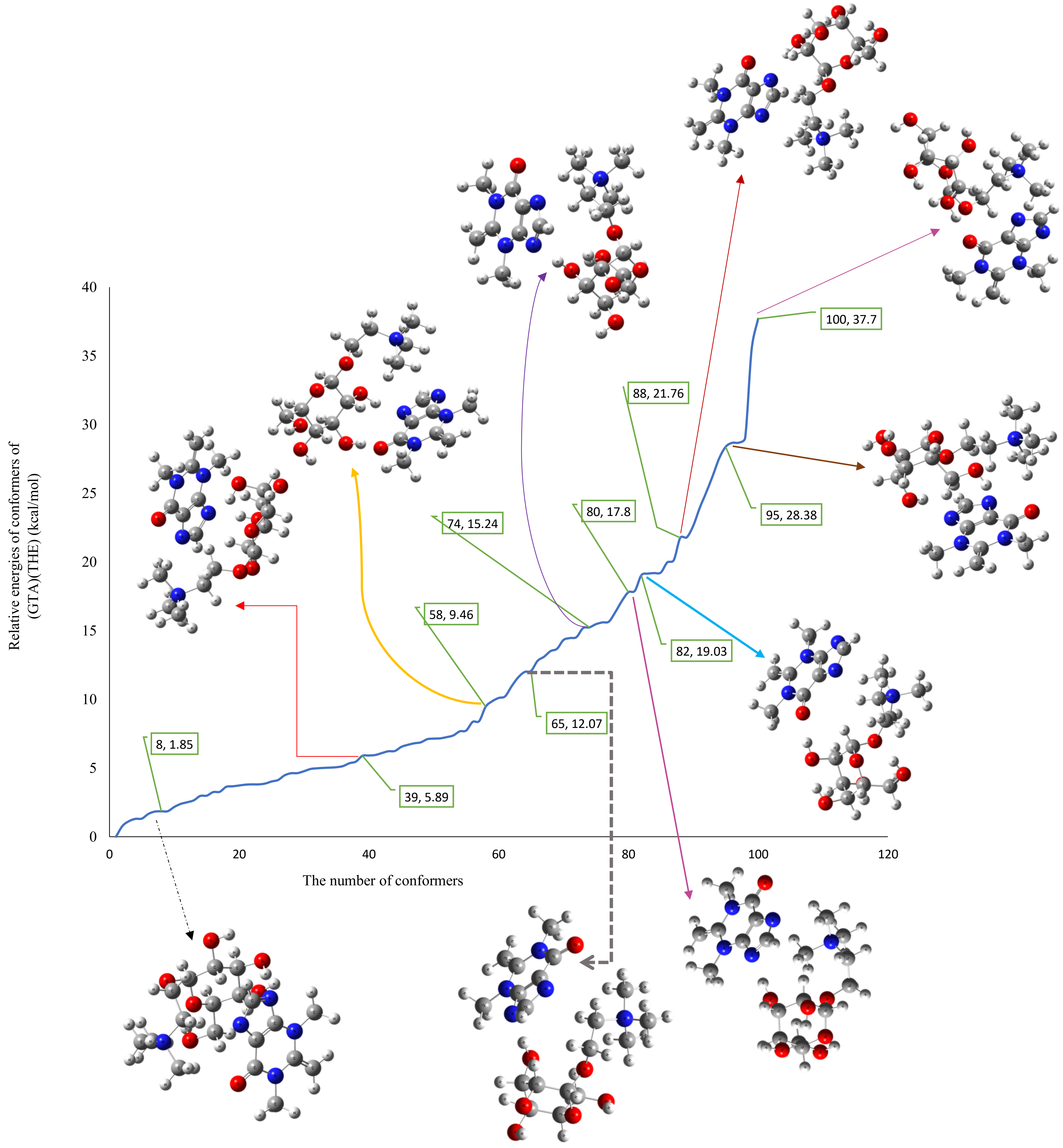

Figure S18. Relative energies of the conformers of (GTA) (THE) with respect to the most stable conformer
