# Supplementary material for: Design of ionic liquids containing glucose and choline as drug carriers, finding the link between QM and MD studies
Source: Sci Rep. 2022 Dec 19;12:21941. doi: 10.1038/s41598-022-25963-z (PMC9763358; doi:10.1038/s41598-022-25963-z)
Supplement: Supplementary file 5 — Supplementary Information 1. [file 41598_2022_25963_MOESM5_ESM.pdf]

**Table S1.** The geometrical parameters of BMIM-based ILs

| Name of structure | Structure | Type of molecular distances | Atoms in the contacts | Distances (Å) |
|---|---|---|---|---|
| 1-Butyl-3-methylimidazolium -2-(4-isobutylphenyl) propanoate | 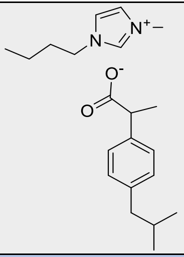 | Intermolecular (between cation and anion) | O35 ……….H12 | 2.459 |
| | | Intermolecular (between cation and anion) | O36 ……….H12 | 1.67 |
| 1-Butyl-3-methylimidazolium-6-methyl-4-oxo-4H-1,2,3-oxathiazin-3-ide 2,2-dioxide | 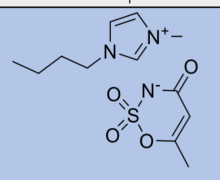 | Intermolecular (between cation and anion) | O8 ……….H26 | 2.563 |
| 1-Butyl-3-methylimidazolium-2-hydroxybenzoate | 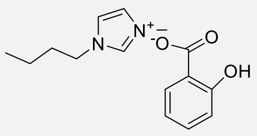 | Intermolecular (between cation and anion) | O33 ……….H12 | 2.410 |
| | | Intermolecular (between cation and anion) | O34 ……….H12 | 1.766 |
| | | Intramolecular | O34 ……….H40 | 1.616 |
| | | Intramolecular | O33 ……….H36 | 2.469 |
| | | Intramolecular | O35 ……….H39 | 2.569 |
| 1-Butyl-3-methylimidazolium-1-ethyl-7-methyl-4-oxo-1,4-dihydro-1,8-naphthyridine-3-carboxylate | 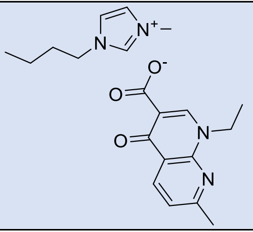 | Intermolecular (between cation and anion) | O41 ……….H12 | 2.666 |
| | | Intermolecular (between cation and anion) | O42 ……….H12 | 1.713 |
| 1-Butyl-3-methylimidazolium-2-(2-((2,6-dichlorophenyl) amino) phenyl) acetate | 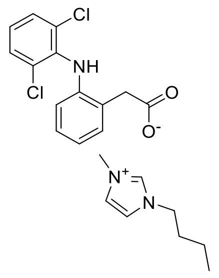 | Intermolecular (between cation and anion) | O10 ……….H41 | 2.478 |
| | | Intermolecular (between cation and anion) | O11 ……….H41 | 1.748 |
| 1-Butyl-3-methylimidazolium-2-(2-fluoro-[1,1'-biphenyl]-4-yl) propanoate | 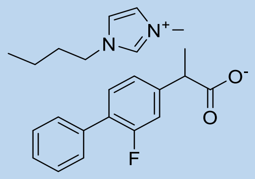 | Intermolecular (between cation and anion) | O16 ……….H42 | 1.716 |
| | | Intermolecular (between cation and anion) | O17 ……….H42 | 2.43 |
| 1-Butyl-3-methylimidazolium-2-(1,8-diethyl-1,3,4,9-tetrahydropyrano[3,4-b] indol-1-yl) acetate | 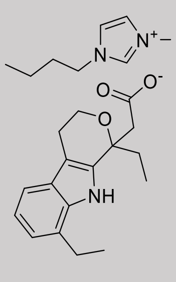 | Intermolecular (between cation and anion) | O20 ……….H53 | 1.786 |
| | | Intermolecular (between cation and anion) | O21 ……….H53 | 2.402 |
| | | Intramolecular | O20 ……….H25 | 1.849 |
| 1-Butyl-3-methylimidazolium-1,3-dimethyl-2-methylene-6-oxo-1,2,3,6-tetrahydropurin-7-ide | 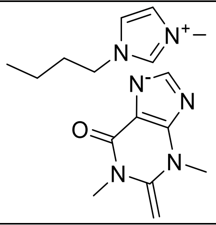 | Intermolecular (between cation and anion) | N9 ……….H34 | 2.163 |
| | | Intermolecular (between cation and anion) | O11 ……….H34 | 1.826 |

**Table S1.** (Continued)

| Name of structure | Structure | Type of molecular distances | Atoms in the contacts | Distances (Å) |
|---|---|---|---|---|
| 3-methyl-1-octyl-1H-imidazol-3-ium- 2-hydroxybenzoate | 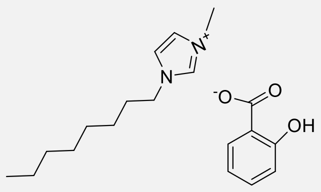 | Intermolecular (between cation and anion) | O8 ……….H31 | 2.449 |
| | | Intermolecular (between cation and anion) | O10 ……….H31 | 1.766 |
| | | Intramolecular | O10 ……….H15 | 1.616 |
| | | Intramolecular | O8 ……….H11 | 2.449 |
| | | Intramolecular | O9 ……….H14 | 2.569 |

3-methyl-1-octyl-1H-imidazol-3-ium- 2-hydroxybenzoate was added to Table S1 for comparison with 1-Butyl-3-methylimidazolium-2-hydroxybenzoate

**Table S2.** The geometrical parameters of N,N,N-trimethyl-2-(((2R,3R,4S,5S,6R)-3,4,5-trihydroxy-6-(hydroxymethyl)tetrahydro-2H-pyran-2-yl)oxy)ethan-1-aminium (GTA) -based ILs

| Name of structure | Structure | Type of molecular distances | Atoms in the contacts | Distances (Å) |
|---|---|---|---|---|
| N,N,N-trimethyl-2-(((2R,3R,4S,5S,6R)-3,4,5-trihydroxy-6-(hydroxymethyl)tetrahydro-2H-pyran-2-yl)oxy)ethan-1-aminium -2-(4-isobutylphenyl) propanoate | 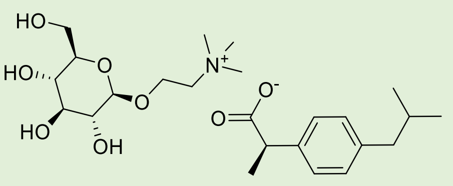 | Intermolecular (between cation and anion) | O11 ……….H63 | 2.187 |
| | | Intermolecular (between cation and anion) | O11 ……….H64 | 2.302 |
| | | Intermolecular (between cation and anion) | O11 ……….H70 | 2.125 |
| | | Intermolecular (between cation and anion) | O10 ……….H74 | 1.659 |
| | | Intermolecular (between cation and anion) | O10 ……….H60 | 1.907 |
| | | Intramolecular | O42 ……….H59 | 2.366 |
| | | Intramolecular | O37 ……….H58 | 2.362 |
| | | Intramolecular | O49 ……….H66 | 2.346 |
| N,N,N-trimethyl-2-(((2R,3R,4S,5S,6R)-3,4,5-trihydroxy-6-(hydroxymethyl)tetrahydro-2H-pyran-2-yl)oxy)ethan-1-aminium - 6-methyl-4-oxo-4H-1,2,3-oxathiazin-3-ide 2,2-dioxide | 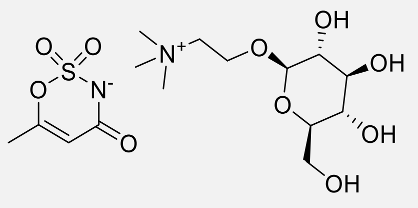 | Intermolecular (between cation and anion) | O7 ……….H49 | 1.827 |
| | | Intermolecular (between cation and anion) | O7 ……….H46 | 2.426 |
| | | Intermolecular (between cation and anion) | O7 ……….H51 | 2.153 |
| | | Intermolecular (between cation and anion) | N5 ……….H43 | 2.335 |
| | | Intermolecular (between cation and anion) | O9 ……….H42 | 1.827 |
| | | Intramolecular | O24……….H56 | 2.468 |
| | | Intramolecular | O24……….H41 | 2.467 |
| | | Intramolecular | O19……….H40 | 2.336 |
| N,N,N-trimethyl-2-(((2R,3R,4S,5S,6R)-3,4,5-trihydroxy-6-(hydroxymethyl)tetrahydro-2H-pyran-2-yl)oxy)ethan-1-aminium -2-hydroxybenzoate | 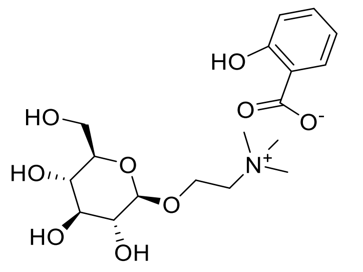 | Intermolecular (between cation and anion) | O50……….H42 | 1.754 |
| | | Intermolecular (between cation and anion) | O50……….H38 | 2.085 |
| | | Intermolecular (between cation and anion) | O51……….H30 | 2.233 |
| | | Intermolecular (between cation and anion) | O51……….H33 | 2.021 |
| | | Intramolecular | O18……….H28 | 2.397 |
| | | Intramolecular | O10……….H27 | 2.411 |
| | | Intramolecular | O5……….H26 | 2.372 |
| | | Intramolecular | O51……….H57 | 1.637 |
| N,N,N-trimethyl-2-(((2R,3R,4S,5S,6R)-3,4,5-trihydroxy-6-(hydroxymethyl)tetrahydro-2H-pyran-2-yl)oxy)ethan-1-aminium -1-ethyl-7-methyl-4-oxo-1,4-dihydro-1,8-naphthyridine-3-carboxylate | 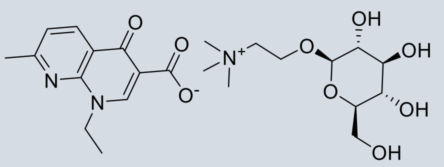 | Intermolecular (between cation and anion) | O17……….H66 | 2.142 |
| | | Intermolecular (between cation and anion) | O17……….H63 | 2.179 |
| | | Intermolecular (between cation and anion) | O17……….H59 | 2.297 |
| | | Intermolecular (between cation and anion) | O11……….H66 | 2.653 |
| | | Intermolecular (between cation and anion) | O16……….H70 | 1.743 |
| | | Intermolecular (between cation and anion) | O16……….H56 | 1.838 |
| | | Intramolecular | O38……….H55 | 2.35 |
| | | Intramolecular | O33……….H54 | 2.35 |

**Table S2.** (Continued)

| Name of structure | Structure | Type of molecular distances | Atoms in the contacts | Distances (Å) |
|---|---|---|---|---|
| N,N,N-trimethyl-2-(((2R,3R,4S,5S,6R)-3,4,5-trihydroxy-6-(hydroxymethyl)tetrahydro-2H-pyran-2-yl)oxy)ethan-1-aminium - 2-(2-((2,6-dichlorophenyl) amino) phenyl) acetate | 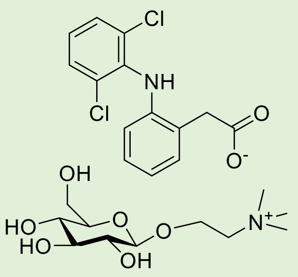 | Intermolecular (between cation and anion) | O10……….H70 | 2.340 |
| | | Intermolecular (between cation and anion) | O10……….H57 | 2.394 |
| | | Intermolecular (between cation and anion) | O11……….H71 | 1.616 |
| | | Intermolecular (between cation and anion) | O47……….H57 | 2.624 |
| | | Intermolecular (between cation and anion) | O11……….H57 | 2.117 |
| | | Intramolecular | O39……….H56 | 2.378 |
| | | Intramolecular | O34……….H55 | 2.372 |
| | | Intramolecular | O46……….H65 | 2.415 |
| | | Intramolecular | O11……….H24 | 2.01 |
| N,N,N-trimethyl-2-(((2R,3R,4S,5S,6R)-3,4,5-trihydroxy-6-(hydroxymethyl)tetrahydro-2H-pyran-2-yl)oxy)ethan-1-aminium -2-(2-fluoro-[1,1'-biphenyl]-4-yl) propanoate | 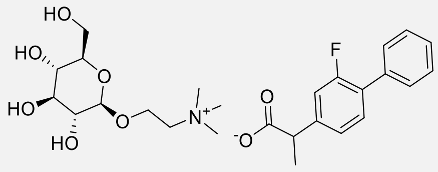 | Intermolecular (between cation and anion) | O17……….H58 | 1.876 |
| | | Intermolecular (between cation and anion) | O17……….H72 | 1.682 |
| | | Intermolecular (between cation and anion) | O16……….H71 | 1.956 |
| | | Intermolecular (between cation and anion) | O16……….H59 | 2.412 |
| | | Intermolecular (between cation and anion) | O48……….H66 | 2.14 |
| | | Intramolecular | O40……….H57 | 2.336 |
| | | Intramolecular | O35……….H56 | 2.367 |
| | | Intramolecular | O48……….H58 | 2.673 |
| N,N,N-trimethyl-2-(((2R,3R,4S,5S,6R)-3,4,5-trihydroxy-6-(hydroxymethyl)tetrahydro-2H-pyran-2-yl)oxy)ethan-1-aminium -2-(1,8-diethyl-1,3,4,9-tetrahydropyrano[3,4-b] indol-1-yl) acetate | 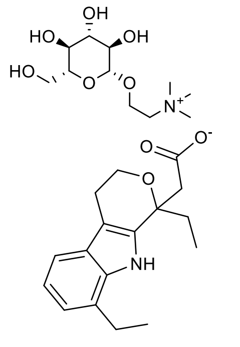 | Intermolecular (between cation and anion) | O20……….H83 | 1.660 |
| | | Intermolecular (between cation and anion) | O20……….H74 | 2.283 |
| | | Intermolecular (between cation and anion) | O21……….H69 | 1.809 |
| | | Intramolecular | O59……….H81 | 2.246 |
| | | Intramolecular | O50……….H67 | 2.043 |
| | | Intramolecular | O51……….H68 | 2.312 |
| | | Intramolecular | O59……….H81 | 2.246 |
| N,N,N-trimethyl-2-(((2R,3R,4S,5S,6R)-3,4,5-trihydroxy-6-(hydroxymethyl)tetrahydro-2H-pyran-2-yl)oxy)ethan-1-aminium -1,3-dimethyl-2-methylene-6-oxo-1,2,3,6-tetrahydropurin-7-ide | 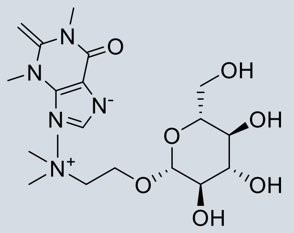 | Intermolecular (between cation and anion) | O11……….H57 | 2.295 |
| | | Intermolecular (between cation and anion) | N9……….H57 | 2.493 |
| | | Intermolecular (between cation and anion) | N7……….H50 | 1.934 |
| | | Intramolecular | O27……….H48 | 2.398 |
| | | Intramolecular | O32……….H49 | 2.383 |
| | | Intramolecular | O32……….H64 | 2.504 |
| | | Intramolecular | O39……….H61 | 2.275 |
| | | Intramolecular | O40……….H56 | 2.552 |

**Table S3.** The average intermolecular distances of ionic liquids (Å) according to the QM and MD studies.

| Name of ionic liquids | Abbreviation | The average of intermolecular distances between anion and cation according to the calculations at the B3LYP/6-311++G(d,p) level (Å) | The average of intermolecular distances between anion and cation according to the results from MD studies (Å) |
|---|---|---|---|
| 1-Butyl-3-methylimidazolium 2-hydroxybenzoate | (BMIM)(SAL) | 2.088 | 2.56 |
| 1-Butyl-3-methylimidazolium-6-methyl-4-oxo-4H-1,2,3-oxathiazin-3-ide 2,2-dioxide | (BMIM)(ACS) | 2.563 | 2.745 |
| 1-Butyl-3-methylimidazolium -2-(4-isobutylphenyl)propanoate | (BMIM)(IBU) | 2.065 | 2.65 |
| 1-Butyl-3-methylimidazolium-2-(2-fluoro-[1,1'-biphenyl]-4-yl)propanoate | (BMIM)(FBP) | 2.073 | 2.598 |
| 1-Butyl-3-methylimidazolium-1-ethyl-7-methyl-4-oxo-1,4-dihydro-1,8-naphthyridine-3-carboxylate | (BMIM)(NAL) | 2.189 | 2.63 |
| N,N,N-trimethyl-2-(((2R,3R,4S,5S,6R)-3,4,5-trihydroxy-6-(hydroxymethyl)tetrahydro-2H-pyran-2-yl)oxy)ethan-1-aminium -2-hydroxybenzoate | (GTA) (SAL) | 2.023 | 2.667 |
| N,N,N-trimethyl-2-(((2R,3R,4S,5S,6R)-3,4,5-trihydroxy-6-(hydroxymethyl)tetrahydro-2H-pyran-2-yl)oxy)ethan-1-aminium - 6-methyl-4-oxo-4H-1,2,3-oxathiazin-3-ide 2,2-dioxide | (GTA)(ACS) | 2.114 | 2.724 |
| N,N,N-trimethyl-2-(((2R,3R,4S,5S,6R)-3,4,5-trihydroxy-6-(hydroxymethyl)tetrahydro-2H-pyran-2-yl)oxy)ethan-1-aminium -2-(4-isobutylphenyl) propanoate | (GTA) (IBU) | 2.036 | 2.385 |
| N,N,N-trimethyl-2-(((2R,3R,4S,5S,6R)-3,4,5-trihydroxy-6-(hydroxymethyl)tetrahydro-2H-pyran-2-yl)oxy)ethan-1-aminium -2-(2-fluoro-[1,1'-biphenyl]-4-yl) propanoate | (GTA)(FBP) | 1.982 | 2.261 |
| N,N,N-trimethyl-2-(((2R,3R,4S,5S,6R)-3,4,5-trihydroxy-6-(hydroxymethyl)tetrahydro-2H-pyran-2-yl)oxy)ethan-1-aminium -1-ethyl-7-methyl-4-oxo-1,4-dihydro-1,8-naphthyridine-3-carboxylate | (GTA) (NAL) | 2.142 | 2.756 |

**Table S4.** The comparison between $\Delta G_{binding}$ (kcal/mol) values calculated by two various methods for BMIM-based ILs and GTA-based ILs.

| Entry | Name of ionic liquids | Abbreviation | $\Delta G_{binding}$ (kcal/mol)) (QM) | $\Delta G_{binding}$ (kcal/mol)) (MD) |
|---|---|---|---|---|
| 1 | 1-Butyl-3-methylimidazolium-2-(2-fluoro-[1,1'-biphenyl]-4-yl) propanoate | (BMIM)(FBP) | -78.4 | -68.36 |
| 2 | N,N,N-trimethyl-2-(((2R,3R,4S,5S,6R)-3,4,5-trihydroxy-6-(hydroxymethyl)tetrahydro-2H-pyran-2-yl)oxy)ethan-1-aminium -2-(2-fluoro-[1,1'-biphenyl]-4-yl)propanoate | (GTA)(FBP) | -75.3 | -57.32 |
| 3 | 1-Butyl-3-methylimidazolium-1-ethyl-7-methyl-4-oxo-1,4-dihydro-1,8-naphthyridine-3-carboxylate | (BMIM)(NAL) | -84.3 | -88.4 |
| 4 | N,N,N-trimethyl-2-(((2R,3R,4S,5S,6R)-3,4,5-trihydroxy-6-(hydroxymethyl)tetrahydro-2H-pyran-2-yl)oxy)ethan-1-aminium -1-ethyl-7-methyl-4-oxo-1,4-dihydro-1,8-naphthyridine-3-carboxylate | (GTA)(NAL) | -86.3 | -70.57 |
| 5 | 1-Butyl-3-methylimidazolium 2-hydroxybenzoate | (BMIM)(SAL) | -74.7 | -103.66 |
| 6 | N,N,N-trimethyl-2-(((2R,3R,4S,5S,6R)-3,4,5-trihydroxy-6-(hydroxymethyl)tetrahydro-2H-pyran-2-yl)oxy)ethan-1-aminium-2-hydroxybenzoate | (GTA)(SAL) | -72.3 | -59.72 |
| 7 | 1-Butyl-3-methylimidazolium -2-(4-isobutylphenyl) propanoate | (BMIM)(IBU) | -79.2 | -65.73 |
| 8 | N,N,N-trimethyl-2-(((2R,3R,4S,5S,6R)-3,4,5-trihydroxy-6-(hydroxymethyl)tetrahydro-2H-pyran-2-yl)oxy)ethan-1-aminium -2-(4-isobutylphenyl)propanoate | (GTA) (IBU) | -81.0 | -59.3 |
| 9 | 1-Butyl-3-methylimidazolium-6-methyl-4-oxo-4H-1,2,3-oxathiazin-3-ide 2,2-dioxide | (BMIM)(ACS) | -67.1 | -9.29 |
| 10 | N,N,N-trimethyl-2-(((2R,3R,4S,5S,6R)-3,4,5-trihydroxy-6-(hydroxymethyl)tetrahydro-2H-pyran-2-yl)oxy)ethan-1-aminium- 6-methyl-4-oxo-4H-1,2,3-oxathiazin-3-ide 2,2-dioxide | (GTA)(ACS) | -67.8 | -4.03 |

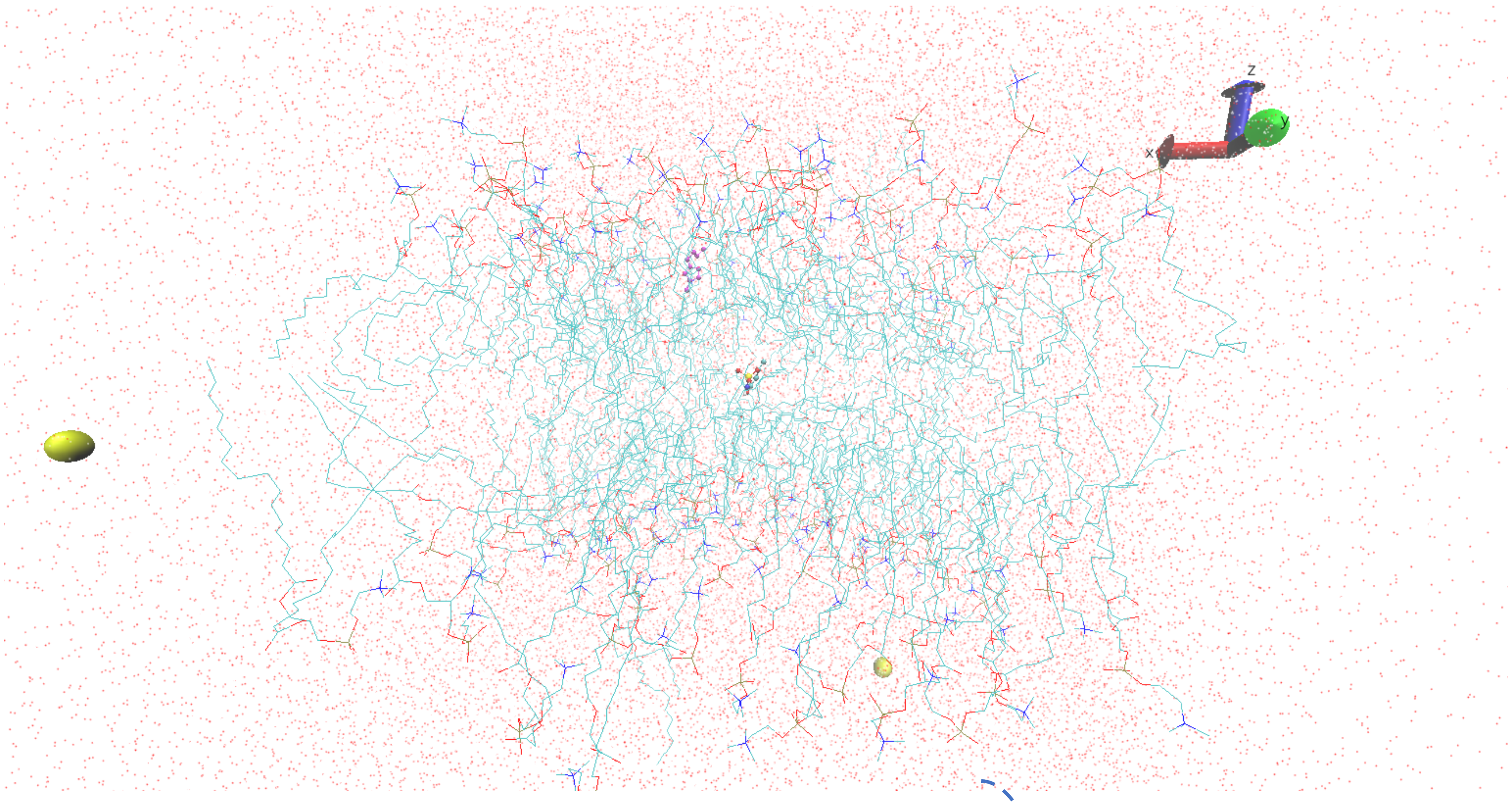


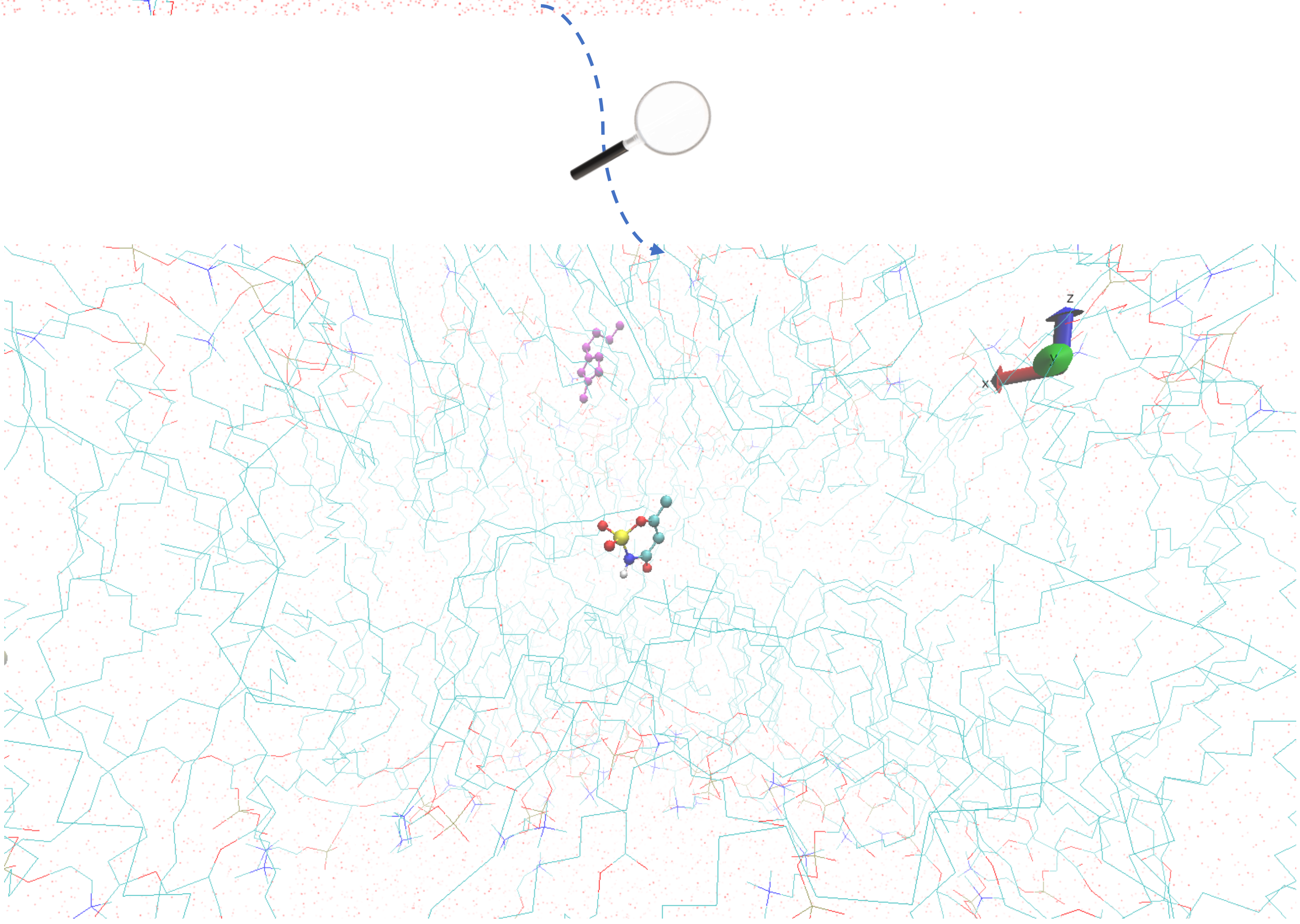


**Figure S19.** In the MD simulation of (BMIM)(ACS) in the membrane structure, the cation has been shown in pink colour and by the ball and stick model. Anion (ACS) has been given by the ball and stick model. The membrane structure has been displayed by line style, and chloride ions are yellow. Molecules of water (solvent) can be seen in red colour and are shown by dots.

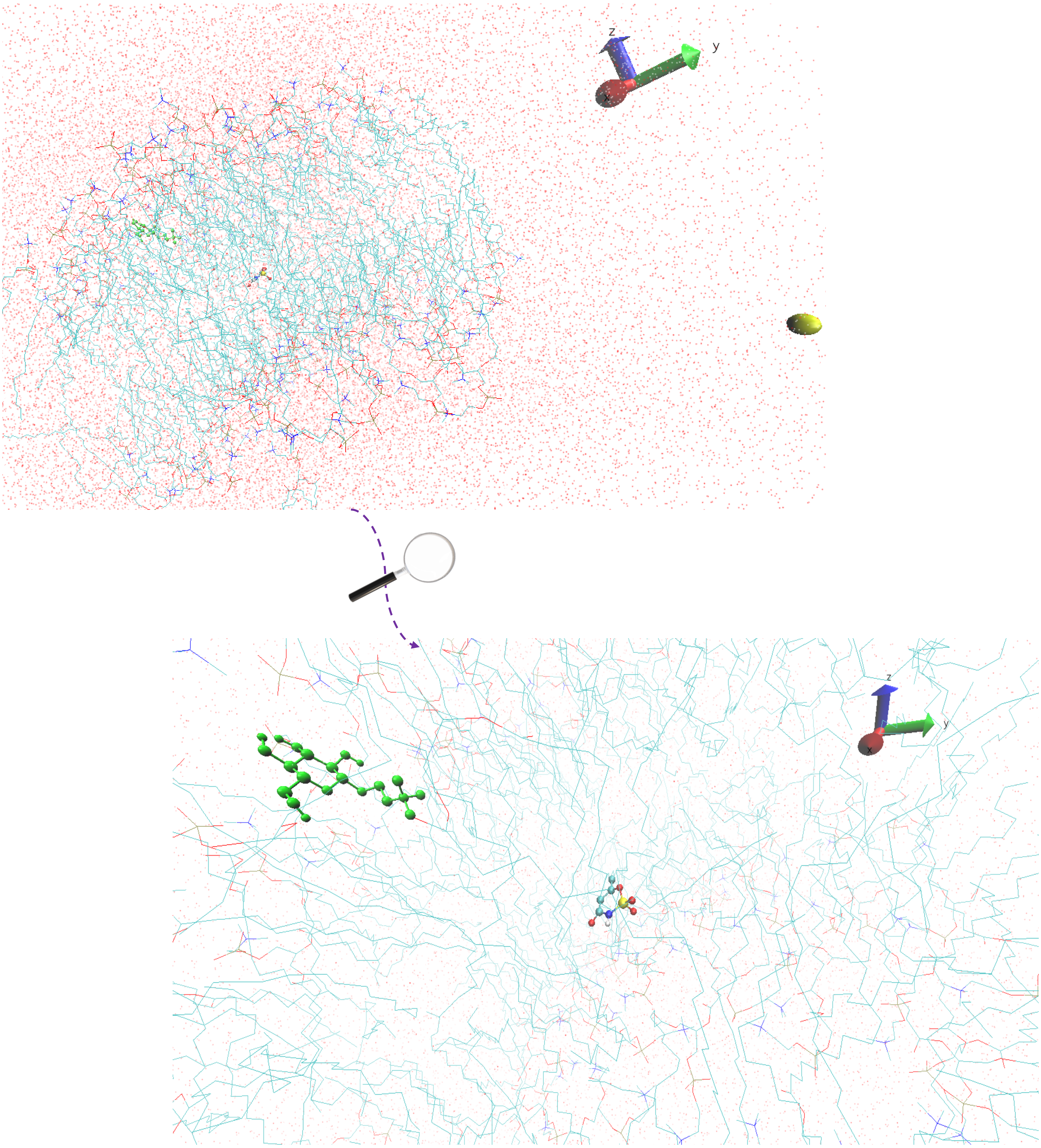


**Figure S20.** In the MD simulation of (GTA)(ACS) in the membrane structure, the cation has been shown in green colour and by the ball and stick model. Anion (ACS) has been given by the ball and stick model. The membrane structure has been displayed by line style, and chloride ions are yellow. Molecules of water (solvent) can be seen in red colour and are shown by dots.

**Table S5.** The computed equilibrium constants related to the formation of BMIM-based ionic liquids and GTA based by use of equation 6

| Entry | Abbreviation of ILs | $\Delta G_{binding}$ (kcal/mol)) (QM) | K (from QM studies) | $\Delta G_{binding}$ (kcal/mol)) (MD) | K (from MD studies) |
|---|---|---|---|---|---|
| 1 | (BMIM)(FBP) | -78.4 | $3.2\times10^{57}$ | -68.36 | $1.4\times10^{50}$ |
| 2 | (GTA)(FBP) | -75.3 | $1.7\times10^{55}$ | -57.32 | $1.1\times10^{42}$ |
| 3 | (BMIM)(NAL) | -84.3 | $6.8\times10^{61}$ | -88.4 | $6.9\times10^{64}$ |
| 4 | (GTA)(NAL) | -86.3 | $2.0\times10^{63}$ | -70.57 | $5.7\times10^{51}$ |
| 5 | (BMIM)(SAL) | -74.7 | $6.1\times10^{54}$ | -103.66 | $1.1\times10^{76}$ |
| 6 | (GTA)(SAL) | -72.3 | $1.1\times10^{53}$ | -59.72 | $6.3\times10^{43}$ |
| 7 | (BMIM)(IBU) | -79.2 | $1.2\times10^{58}$ | -65.73 | $1.6\times10^{48}$ |
| 8 | (GTA) (IBU) | -81 | $2.6\times10^{59}$ | -59.3 | $3.1\times10^{43}$ |
| 9 | (BMIM)(ACS) | -67.1 | $1.6\times10^{49}$ | -9.29 | $6.5\times10^{6}$ |
| 10 | (GTA)(ACS) | -67.8 | $5.3\times10^{49}$ | -4.03 | $9\times10^{2}$ |

**Table S6.** $\Delta E_{int}$ and $\Delta E_{CEC}$ (kcal/mol) values of the known ILs including: (a) (BMIM)($BF_4$), (b) (BMIM)Br, (c) (BMIM)Cl, (d) (BMIM)(TFSI), and (e) (BMIM)($PF_6$) calculated at B3LYP/6-311++ G (d, p) level alongside with their experimental melting points ºC.

| Name of structures | Abbreviation | $E_{SCF}$ (Hartree/Particle) | $\Delta E_{int}$ (kcal/mol) | $\Delta E_{CEC}$ (kcal/mol) | Melting point, ºC |
|---|---|---|---|---|---|
| **tetrafluoroborate** | ($BF_4$) | -424.6616 | | | |
| **1-butyl-3-methylimidazolium** | (BMIM) | -423.05161 | | | |
| **chloride** | Cl | -460.30231 | | | |
| **1-butyl-3-methylimidazolium chloride** | (BMIM) Cl | -883.49464 | -88.3 | 88.3 | 89 |
| **1-butyl-3-methylimidazolium tetrafluoroborate** | (BMIM) (BF4) | -847.84118 | -80.3 | 80.3 | -82 |
| **bromide** | Br | -423.05161 | | | |
| **1-butyl-3-methylimidazolium bromide** | (BMIM) Br | -2997.42117 | -83.6 | 83.6 | 60 |
| **hexafluorophosphate** | ($PF_6$) | -940.87213 | | | |
| **1-butyl-3-methylimidazolium hexafluorophosphate** | (BMIM) ($PF_6$) | -1364.04423 | -75.6 | 75.6 | 10 |
| **trifluoromethyl sulfonate** | (TF) | -961.69669 | | | |
| **1-butyl-3-methylimidazolium trifluoromethyl sulfonate** | (BMIM)(TF) | -1384.87053 | -76.7 | 76.7 | -15 |
| **bis (trifluoromethyl sulfonyl) amide** | TFSI | -1827.54373 | | | |
| **1-butyl-3-methylimidazolium bis (trifluoromethyl sulfonyl) amide** | (BMIM)(TFSI) | -2250.70975 | -71.8 | 71.8 | -8 |

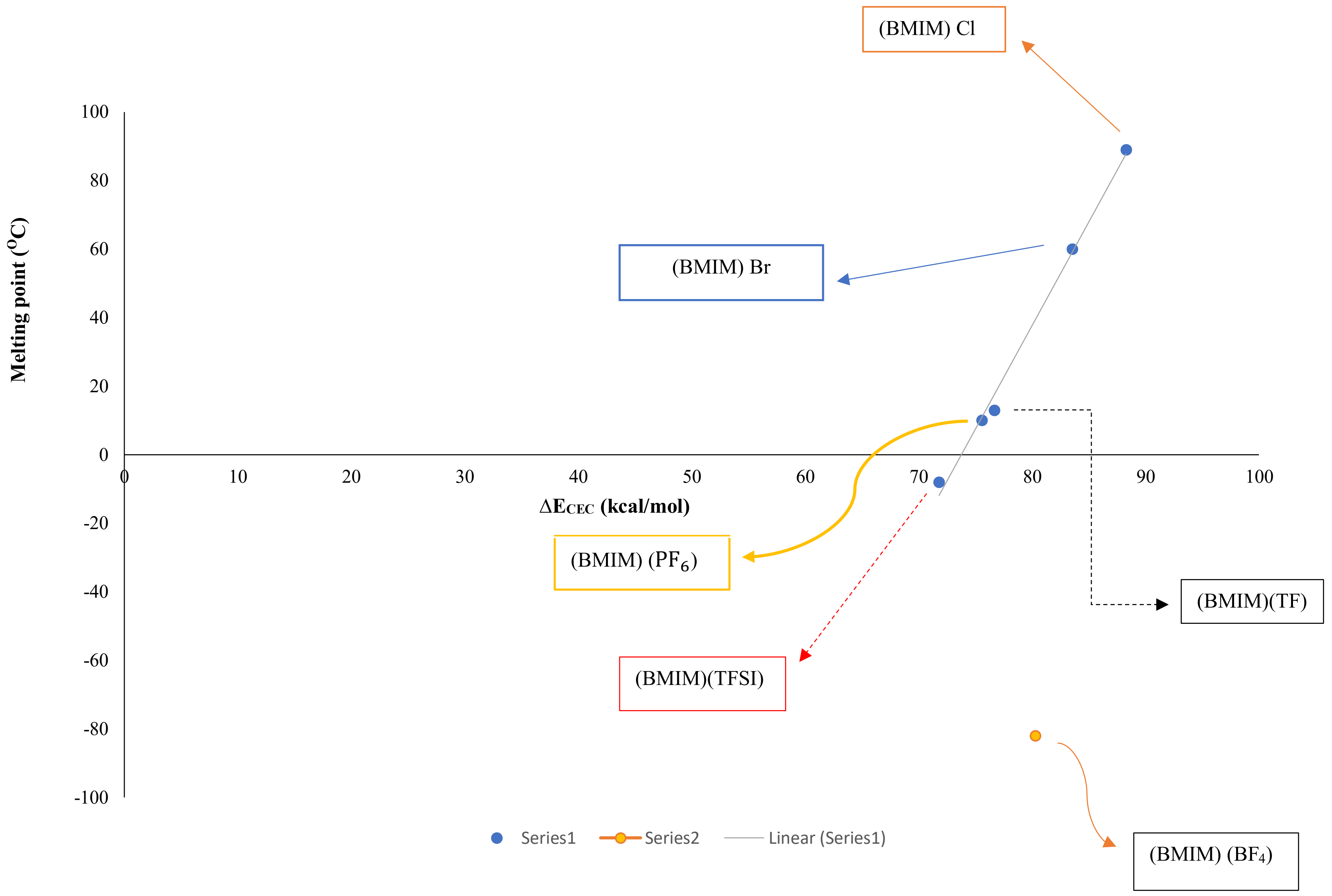


**Figure S21.** Linear relationship between theoretically calculated $\Delta E_{CEC}$ (kcal /mol) of some known BMIM-based ionic liquids and their experimentally measured melting points ºC.
