# Supplementary material for: Design of ionic liquids containing glucose and choline as drug carriers, finding the link between QM and MD studies
Source: Sci Rep. 2022 Dec 19;12:21941. doi: 10.1038/s41598-022-25963-z (PMC9763358; doi:10.1038/s41598-022-25963-z)
Supplement: Supplementary file 6 — Supplementary Information 2. [file 41598_2022_25963_MOESM6_ESM.pdf]

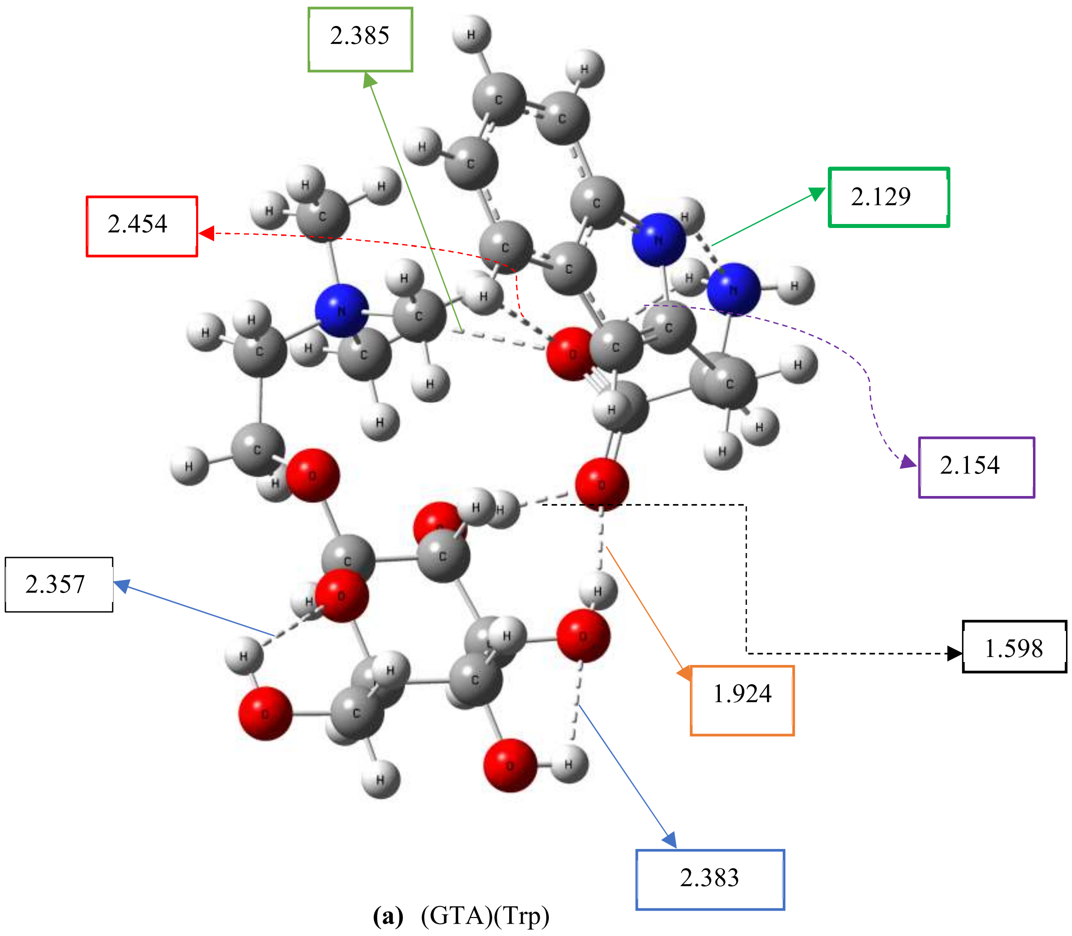

(a) (GTA)(Trp)

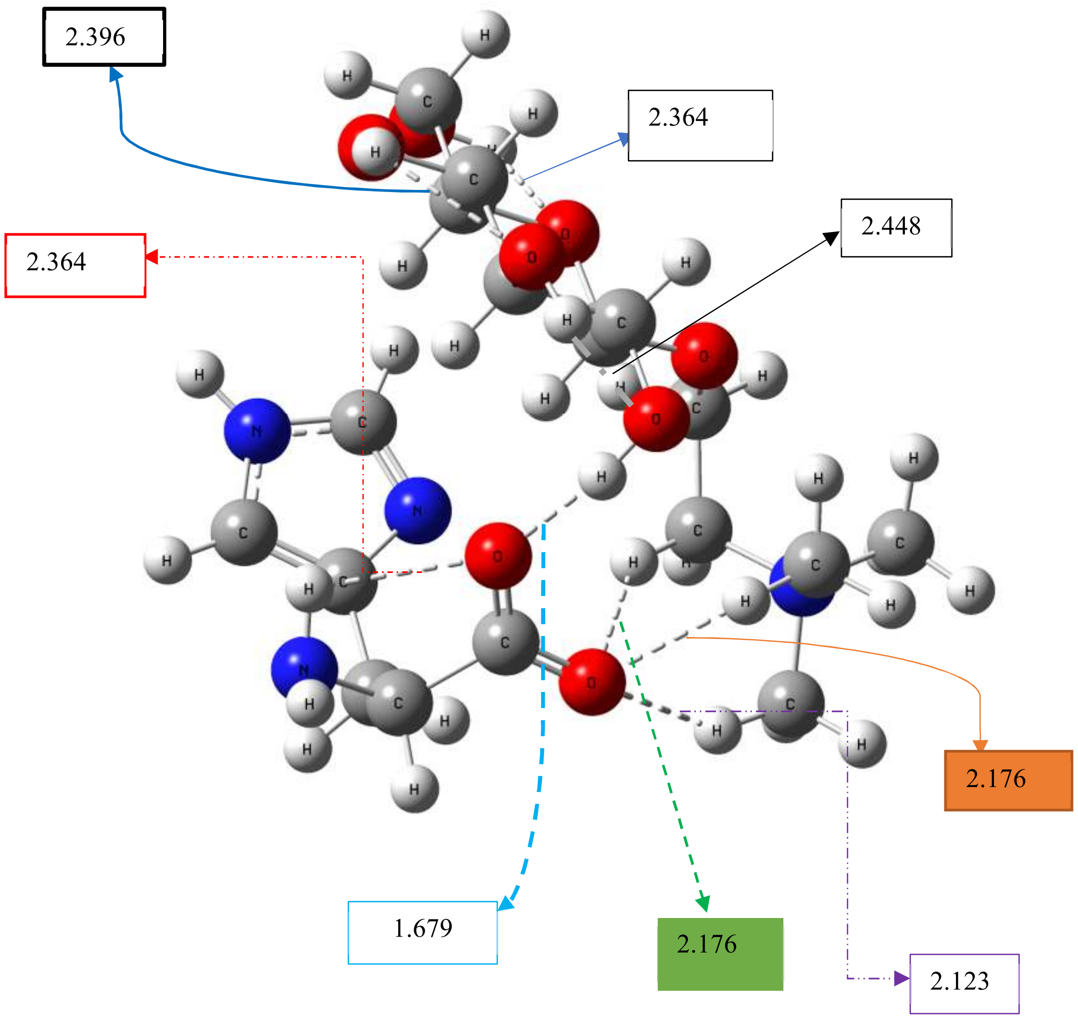

(b) (GTA)(His)

Figure S22. Optimized geometries of the lowest-energy conformers of GTA-based ILs including; (GTA)(Trp), (GTA)(His) and (GTA)(Tyr) calculated at the B3LYP/6-311++G(d,p) level.

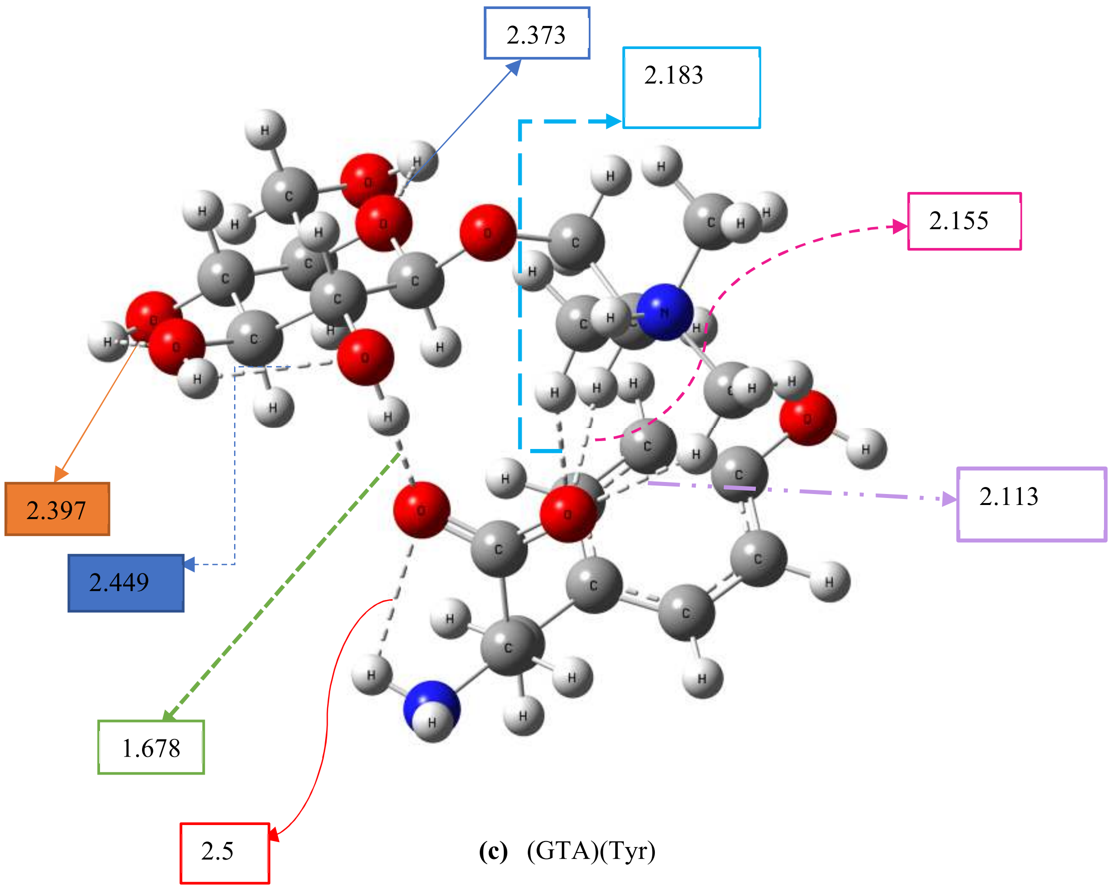

(c) (GTA)(Tyr)

Figure S22. Optimized geometries of the lowest-energy conformers of GTA-based ILs including; (GTA)(Trp), (GTA)(His) and (GTA)(Tyr) calculated at the B3LYP/6-311++G(d,p) level.

Table S7. ΔE_{int} and ΔE_{CEC} of the ionic liquids including; (GTA)(Trp), (GTA)(His) and (GTA)(Tyr) calculated at the B3LYP/6-311++G(d,p) Level by our research group and their experimentally reported decomposition temperatures (°C) [21].

Entry	Name of structures	Structure	Codes of structures	E_{SCF} (Hartree per Particle)	ΔE_{int} (kcal. mol^{-1})	ΔE_{CEC} (kcal. mol^{-1})	T_d (°C)
1	N,N,N-trimethyl-2-(((2R,3R,4S,5S,6R)-3,4,5-trihydroxy-6-(hydroxymethyl)tetrahydro-2H-pyran-2-yl)oxy)ethan-1-aminium	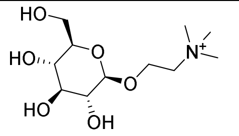	(GTA)	-939.3519			
2	L-tryptophanate	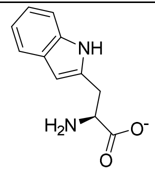	(Trp)	-685.8247			
3	L-histidinate	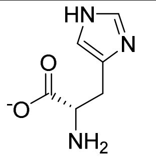	(His)	-548.2313			
4	L-tyrosinate	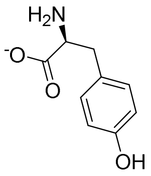	(Tyr)	-629.4704			
5	N,N,N-trimethyl-2-(((2R,3R,4S,5S,6R)-3,4,5-trihydroxy-6-(hydroxymethyl)tetrahydro-2H-pyran-2-yl)oxy)ethan-1-aminium - L-tryptophanate	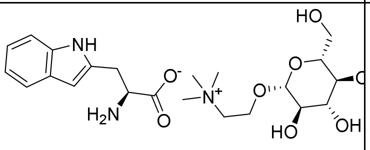	(GTA)(Trp)	-1625.3075	-82.2	82.2	211
6	N,N,N-trimethyl-2-(((2R,3R,4S,5S,6R)-3,4,5-trihydroxy-6-(hydroxymethyl)tetrahydro-2H-pyran-2-yl)oxy)ethan-1-aminium - L-histidinate	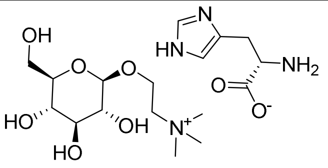	(GTA)(His)	-1487.7358	-95.8	95.8	207
7	N,N,N-trimethyl-2-(((2R,3R,4S,5S,6R)-3,4,5-trihydroxy-6-(hydroxymethyl)tetrahydro-2H-pyran-2-yl)oxy)ethan-1-aminium - L-tyrosinate	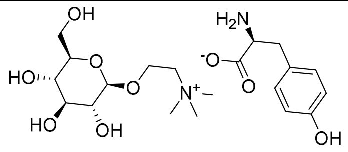	(GTA)(Tyr)	-1568.9679	-91.4	91.4	209

Table S8. Toxicity results for similar chemicals to the structure of BMIM and BMIM, calculated by Toxicity Estimation Software Tool (TEST).

Entry	CAS	Name of the structures	Structure	Similarity Coefficient	Experimental value -Log_{10}(mol/kg)	Predicted value -Log_{10}(mol/kg)
1	80432-08-2 (test chemical)	1-Butyl-3-methylimidazolium	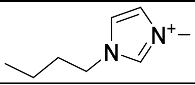	1.00	N/A	1.97
2	2764-72-9	6,7-dihydrodipyrido[1,2-a:2',1'-c]pyrazine-5,8-diium	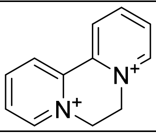	0.87	2.90	2.54
3	49866-87-7	1,2-dimethyl-3,5-diphenyl-1H-pyrazol-2-ium	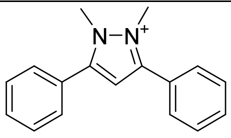	0.76	2.73	2.50
4	2121-12-2	2-hydroxy-4,5-dihydro-[1,3]dioxolo[4,5-j]pyrrolo[3,2,1-de]phenanthridin-6-ium	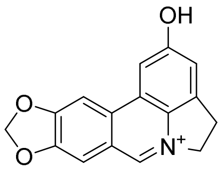	0.60	3.47	3.42
5	14504-15-5	4-(2-amino-2-oxoethyl)-3-benzyl-1,2,3-oxadiazol-3-ium-5-olate	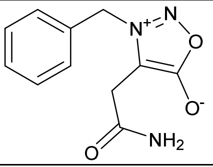	0.60	1.72	1.95
6	86701-22-6	3-{2-[2-Ethoxy-1-propen-1-yl]-1,3-benzoxazol-3-ium-3-yl}-1-propanesulfonate	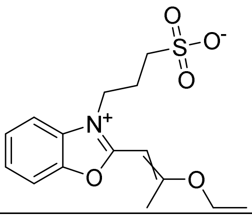	0.55	1.47	1.83
7	535-83-1	1-methylpyridin-1-ium-3-carboxylate	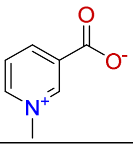	0.51	1.44	1.14

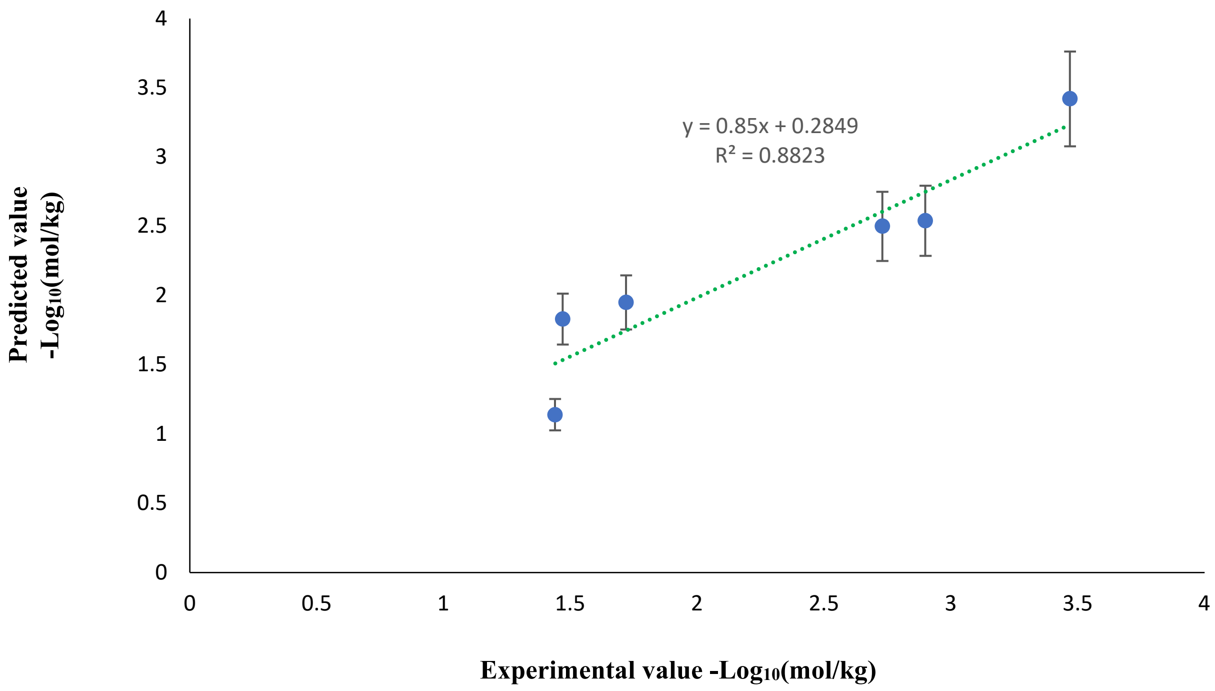

Figure S23. The predicted value of -Log Toxicity (mol/kg) by TEST for similar chemicals to the structure of BMIM against the experimentally reported -Log Toxicity (mol/kg) of these structures.

Table S9. Predicted Oral rat LD_{50} for BMIM from Hierarchical clustering method.

Endpoint	Experimental value	Predicted value	Prediction interval
Oral rat LD_{50} $-Log_{10}$(mol/kg)	N/A	1.97	$1.46 \leq Tox \leq 2.48$
Oral rat LD_{50} mg/kg	N/A	1499.22	$463.61 \leq Tox \leq 4848.12$

Table S10. Toxicity results for similar chemicals to the structure of GTA and GTA, calculated by Toxicity Estimation Software Tool (TEST).

Entry	CAS	Name of the structures	Structure	Similarity Coefficient	Experimental value -Log_{10}(mol/kg)	Predicted value -Log_{10}(mol/kg)
**1**		N-[2-(D-glucopyranosyl) ethyl]-N, N, N-trimethylammonium	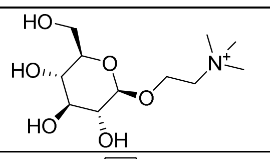	1.00	N/A	1.73
**2**	14007-49-9	4-amino-N-ethyl-N,N-dimethyl-4-oxo-3,3-diphenylbutan-1-aminium	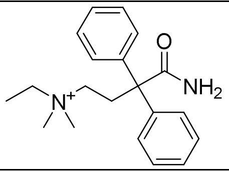	0.81	2.36	2.37
**3**	13473-38-6	1-ethyl-3-(2-hydroxy-2,2-diphenylacetoxy)-1-methylpiperidin-1-ium	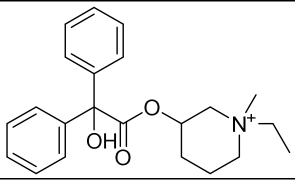	0.71	2.62	2.69
**4**	15503-86-3	(5R,6S,9a^{1}R,14aR, Z)-3-ethylidene-6-hydroxy-6-(hydroxymethyl)-5-methyl-2,7-dioxo-2,3,4,5,6,7,9,9a^{1},11,13,14,14a-dodecahydro-12H-[1,6]dioxacyclododecino[2,3,4-gh]pyrrolizine 12-oxide	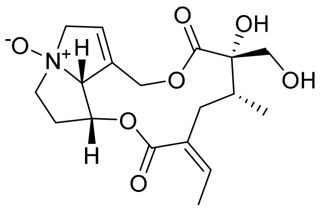	0.69	3.88	3.87
**5**	58066-85-6	hexadecyl (2-(trimethylammonio)ethyl) phosphate	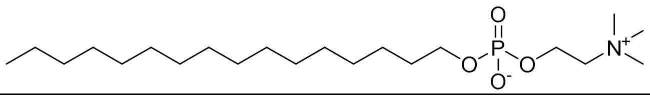	0.68	3.22	3.23
**6**	987-78-0	2-((((((2R,3S,4R,5R)-5-(4-amino-2-oxopyrimidin-1(2H)-yl)-3,4-dihydroxytetrahydrofuran-2-yl)methoxy)(hydroxy)phosphoryl)oxy)oxidophosphoryl)oxy)-N,N,N-trimethylethan-1-aminium	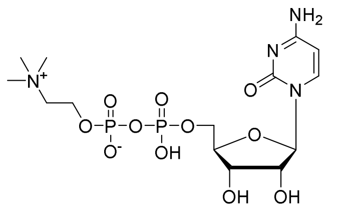	0.67	1.42	1.48
**7**	2747-31-1	N,N-dimethyl-4-(phenyldiazenyl)aniline oxide	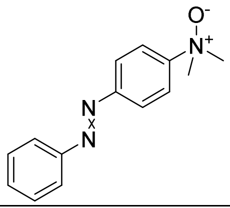	0.67	2.04	2.57
**8**	4317-14-0	3-(10,11-dihydro-5H-dibenzo[a,d][7]annulen-5-ylidene)-N,N-dimethylpropan-1-amine oxide	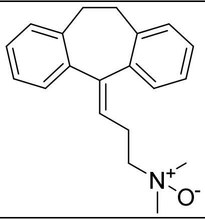	0.66	2.21	2.85
**9**	2207-85-4	5-(3-(dimethylamino)propyl)-10,11-dihydro-5H-dibenzo[b,f]azepine 5-oxide	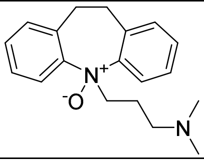	0.66	3.46	3.17
**10**	83519-04-4	3-(hexadecylthio)-2-(methoxymethyl)propyl (2-(trimethylammonio)ethyl) phosphate	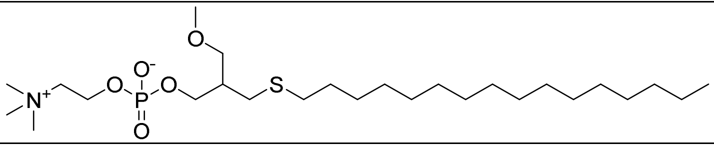	0.64	3.32	3.22
**11**	146764-26-3	2-(1-methylpiperidin-1-ium-1-yl)ethyl octadecyl phosphate	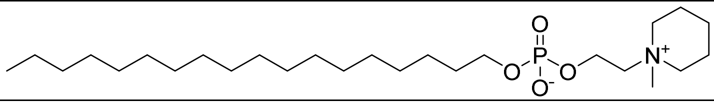	0.63	2.88	2.86

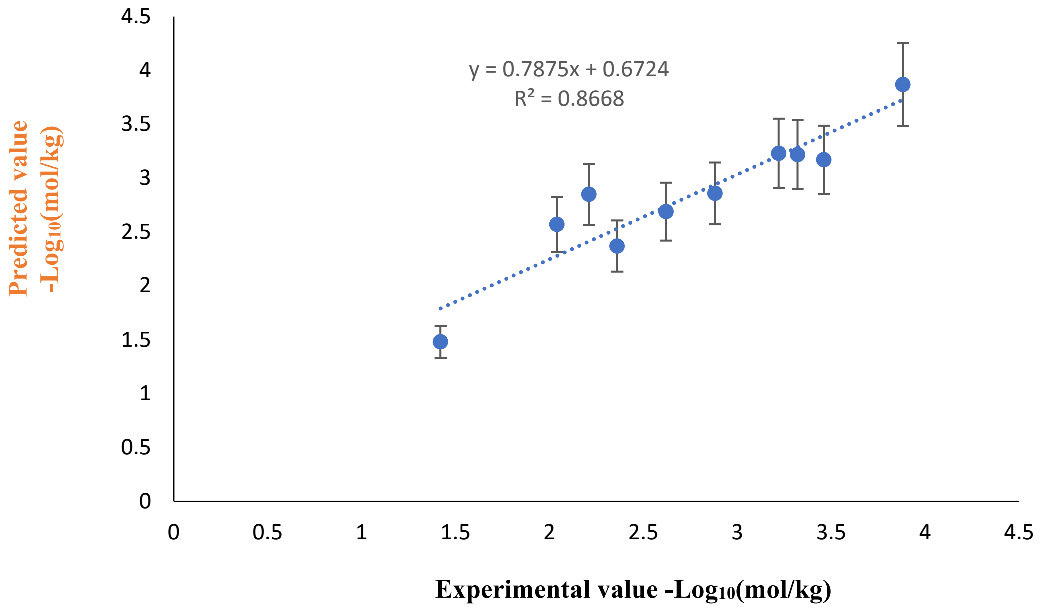

Figure S24. The predicted value of -Log Toxicity (mol/kg) by TEST for similar chemicals to the structure of GTA against the experimentally reported -Log Toxicity (mol/kg) of these structures.

Table S11. Predicted Oral rat LD_{50} for GTA from Hierarchical clustering method.

Endpoint	Experimental value	Predicted value	Prediction interval
Oral rat LD_{50} $-Log_{10}$(mol/kg)	N/A	1.73	$1.16 \leq Tox \leq 2.30$
Oral rat LD_{50} mg/kg	N/A	4955.34	$1342.82 \leq Tox \leq 18286.38$
